# Genome-wide construction of a series of designed segmental aneuploids in *Saccharomyces cerevisiae*

**DOI:** 10.1038/srep12510

**Published:** 2015-07-30

**Authors:** Waranya Natesuntorn, Kotaro Iwami, Yuki Matsubara, Yu Sasano, Minetaka Sugiyama, Yoshinobu Kaneko, Satoshi Harashima

**Affiliations:** 1 Department of Biotechnology, Graduate School of Engineering, Osaka University, 2-1 Yamadaoka, Suita, Osaka, 565-0871, Japan

## Abstract

Segmental aneuploidy can play an important role in environmental adaptation. However, study of segmental aneuploids is severely hampered by the difficulty of creating them in a designed fashion. Here, we describe a PCR-mediated chromosome duplication (PCDup) technology that enables the generation of segmental aneuploidy at any desired chromosomal region in *Saccharomyces cerevisiae*. We constructed multiple strains harboring 100 kb to 200 kb segmental duplications covering the whole of the *S. cerevisiae* genome. Interestingly, some segmental aneuploidies confer stress tolerance, such as to high temperature, ethanol and strong acids, while others induce cell lethality and stress sensitivity, presumably as result of the simultaneous increases in dosages of multiple genes. We suggest that our PCDup technology will accelerate studies into the phenotypic changes resulting from alteration of gene dosage balance of multiple genes and will provide new insights into the adaptive molecular mechanisms in the genome in segmental aneuploidy-derived human diseases.

The development and application of high-throughput genome analysis methods, such as comparative genomic hybridization and next-generation sequencing^1^, have made it relatively easy to identify and analyze most types of novel genetic change not only at the chromosomal but also at the sub-chromosomal level. However, not all chromosomal changes are amenable to analysis by these new approaches. Although high-throughput genome analysis can detect chromosome copy number variation including segmental aneuploidy, it cannot distinguish among types of segmental duplication, such as tandem duplications, duplications inserted into an independent chromosome or generation of independent chromosome. Segmental duplications involving large chromosomal regions are associated with both adverse and beneficial effects in different organisms^2^,^3^,^4^,^5^,^6^,^7^,^8^,^9^,^10^,^11^,^12^,^13^,^14^,^15^,^16^,^17^,^18^,^19^,^20^,^21^,^22^ and result from various types of spontaneous chromosomal mutation, such as tandem intra-chromosomal duplication, inter-chromosomal duplication by translocation, supernumerary chromosomes (structurally abnormal extra chromosomes) and episomal (ring) chromosomes^23^. In this report, we use the term “segmental duplication” to refer to amplification of a particular chromosomal region and “segmental aneuploidy” to refer to a duplication in which the chromosomal region is present as an independent chromosome.

In yeast, partial chromosomal duplications may offer an evolutionary advantage through enabling adaptation to particular stresses in the environment^8^,^9^,^20^,^22^. For example, segmental aneuploids are occasionally found in industrial yeast strains such as those used for fermentation of wine and beer^4^,^21^. In *Candida albicans*, a pathogenic yeast, fluconazole resistance is the result of duplication of the left arm of chromosome V^2,3,5^ that contains *ERG11* encoding a target of fluconazole and *TAC1* encoding a transcription regulator of the ABC transporter. However, segmental duplications are generally associated with detrimental effects in multicellular organisms. For example, in maize, segmental duplication causes morphological abnormalities^18^, while in humans, segmental duplication resulting from supernumerary chromosomes are associated with tumor development and many diseases^6^,^7^,^10^,^12^,^13^,^14^,^17^,^19^. Similarly, although Down syndrome in humans is usually due to trisomy 21, it can also result from partial (segmental) aneuploidy of chromosome 21^11^. These various examples illustrate the impact of segmental duplication on phenotype in unicellular and multicellular organisms.

To date, very few organisms have been exploited for segmental aneuploidy research; some studies have been performed in *S. cerevisiae*^16^, Drosophila^24^, maize^18^ and mouse^25^. In contrast to multicellular organisms, a wide range of genetic tools are available to manipulate the *S. cerevisiae* genome and, therefore, *S. cerevisiae* may be the best available model organism for studying segmental aneuploidies. Several methods can be used to duplicate whole chromosomes in yeast, such as treatment with antibiotics that cause chromosome segregation errors^26^, chromosome transfer based on drug selection^27^, disruption of genes involved in chromosome segregation fidelity^28^, induced nondisjunction of specific chromosomes using a conditional centromere^29^, and the progeny produced by meiotic division in polyploids^30^. However, methods for studying segmental aneuploids are much more limited. Most of the information from yeast regarding the relationship of segmental aneuploidy and phenotype is derived from high-throughput analysis of karyotypic changes in natural populations^4^,^9^,^22^ or laboratory-generated strains^31^. In these populations and strains, it is unclear whether the observed phenotypic changes are a direct consequence of segmental aneuploidy and, additionally, it is difficult to delimit the region potentially responsible for any phenotypic changes. Since the available methods are unsuitable for constructing segmental duplications of specific chromosomal regions, we initiated the present study to develop a methodology with this property.

Here, we describe the development of a simple new technology, which we term PCR-mediated chromosome duplication (PCDup), that can be used in budding yeast to duplicate any desired chromosomal region as an independent chromosome. PCDup is able to duplicate regions with lengths from 50 kb to 300 kb. In the present study, we use PCDup to produce a series of approximately 200 kb segmental duplications that cover most of the genome of *S. cerevisiae*. Interestingly, we observed that some chromosomal regions cannot be duplicated; the implications of this result are considered later. Segmental duplication of some chromosomal regions produces enhanced resistance phenotypes or growth defects when cells are grown under stress. We believe that this novel genome engineering technology for generating an additional chromosome consisting of a defined genomic region will not only be valuable for deciphering genome function but also for breeding yeast strains with desirable stress resistance characteristics.

## Results

### PCR-mediated chromosome duplication (PCDup) technology

**Figure 1:**
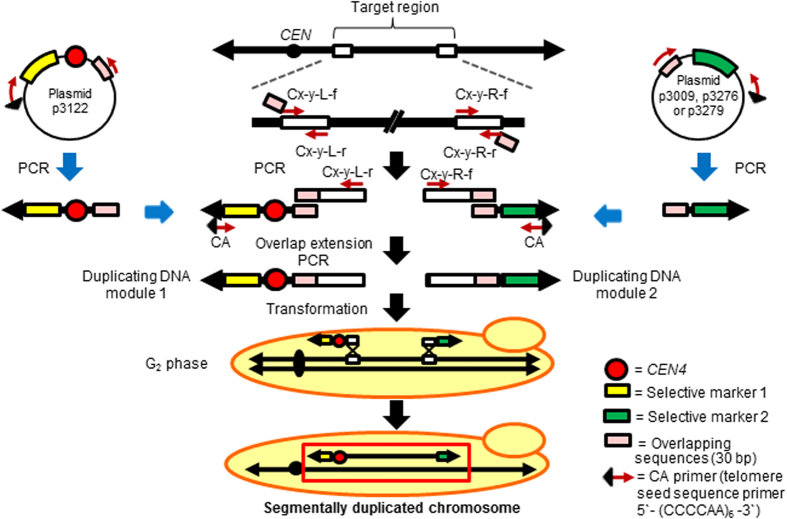
Procedure for construction of a segmentally duplicated chromosome by the PCDup method. Two target DNA fragments with nucleotide sequences corresponding to the left and right ends of the target region (400 bp) were amplified by PCR using genomic DNA as a template and the primers Cx-y-L-f and Cx-y-L-r or Cx-y-R-f and Cx-y-R-r (where x represents chromosome number, y represents chromosome region, L represents left end of sequence of the target region, R represents right end of sequence of the target region, f represents forward primer, and r represents reverse primer). The primer sequences of Cx-y-L-f, Cx-y-L-r, Cx-y-R-f and Cx-y-R-r varied with the target chromosomal region and are listed in Supplementary Table 7. A fragment containing *CEN4* and selective marker 1 cassette and a fragment containing the selective marker 2 cassette were amplified from the plasmid template using loxP-cas and a CA primer (Tables 1, 2 and 4). Next, one target fragment was combined with the *CEN4* and selective marker 1 cassette, and the other target fragment was combined with the selective marker 2 cassette by overlap extension PCR to form two duplicating modules, designated “duplicating DNA module 1” and “duplicating DNA module 2”. The amplified modules were introduced into yeast cells by conventional transformation. The two introduced modules are designed to integrate at the two target sites of the same chromosome by homologous recombination, resulting in duplication of the selected chromosomal region.

An outline of the PCDup method is illustrated in Fig. 1. A detailed description of the preparation of the two types of duplicating DNA module is given in the Methods section. PCDup sought to emulate the characteristics of natural chromosomes in the derived chromosome: stability and the ability to segregate into daughter cells due to the presence of telomeres at both ends of the chromosome, a single centromere, and an autonomously replicating sequence (ARS). To ensure that the chromosomes newly created by PCDup have these characteristics, we prepared a duplicating DNA module containing telomere seed sequences and an additional centromere (duplicating DNA module 1; Fig. 1) and a second duplicating DNA module containing only telomere seed sequences (duplicating module 2; Fig. 1). Since an ARS is expected to be present in every ~40 kb region throughout a natural chromosome^32^, we did not normally add any additional ARSs to the duplicating modules. (However, if a target region was known to be deficient for ARSs, then it would be essential to prepare a duplicating module with such sequences.) If the target region is the terminal part of the chromosome, only one duplicating module is needed to generate a segmentally duplicated chromosome.

The duplicating DNA modules were introduced into yeast cells by conventional transformation. The selected chromosome region was duplicated following simultaneous integration of the two introduced DNA modules into each of the two target sites on the chromosome by homologous recombination. Transformants were identified by culture on a selective medium. The karyotype of the transformants was analyzed using pulsed-field gel electrophoresis (PFGE) and subsequent Southern blot analysis to confirm that the targeted chromosomal region had been duplicated.

### Performance of PCDup

To test the performance of the PCDup method, we first sought to duplicate three chromosomal regions that were selected arbitrarily (Table 1): a 50 kb region of chromosome I, a 145 kb region of chromosome II and a 100 kb region of chromosome X. Our analyses showed that desired duplication was achieved for each of the three regions with a proportion from 10% to 30% (Table 1) based upon the number of transformants having desired karyotype per number of transformants analyzed. This initial experiment therefore confirmed that the PCDup method could duplicate arbitrarily selected chromosomal regions.

### Size of the duplicated region

**Figure 2:**
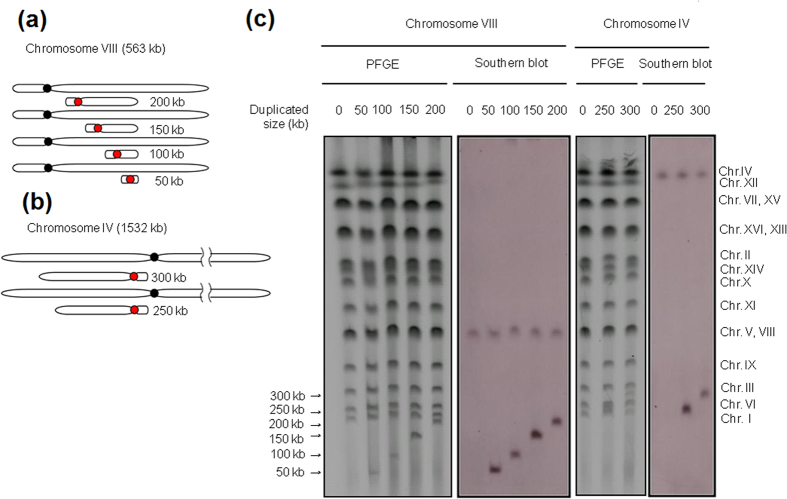
Determination of the maximum size of segmentally duplicated chromosomes by the PCDup method. Segmentally duplicated regions of varying lengths were designed for chromosome VIII (**a**) and chromosome IV (**b**). The probe was prepared by PCR amplification of a 400 bp internal sequence of the target region (red circle represents *CEN4*). (**c**) PFGE and Southern blot analysis of the karyotypes of the 50 kb, 100 kb, 150 kb and 200 kb Chr. VIII segmental aneuploid strains, and the 250 kb and 300 kb Chr. IV segmental aneuploid strains.

Next, we sought to determine the upper size limit of duplicated regions by PCDup. To this end, we constructed a series of segmentally duplicated chromosomes of increasing size (50 kb, 100 kb, 150 kb and 200 kb of chromosome VIII, and 250 kb, 300 kb, 350 kb and 400 kb of chromosome IV) (Table 1). We found that the method reliably duplicated 50, 100, 150, 200 and 300 kb chromosomal regions but not 350 or 400 kb regions. Thus, we concluded that approximately 300 kb was the maximum size of region that PCDup was able to duplicate routinely (Fig. 2 and Table 1). The possible reasons for this size limitation are discussed later.

### Genome-wide construction of segmental duplications by PCDup

**Figure 3:**
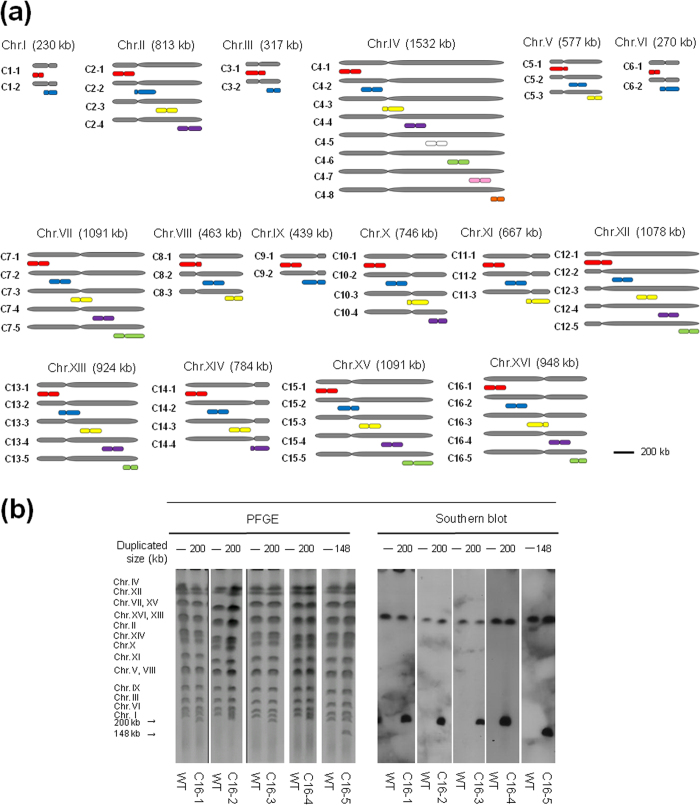
Systematic segmental duplication of chromosomes I to XVI. (**a**) Schematic illustration of a complete set of 62 segmental aneuploid strains covering the whole genome of *S. cerevisiae*. Each chromosome was divided into approximately 200 kb regions and we attempted to duplicate these using the PCDup method. (**b**) PFGE and Southern blot analysis of the karyotypes of segmental aneuploids of chromosome XVI; this chromosome is 948 kb in length and was divided into four regions of 200 kb region and a 148 kb region (designated as C16-1, C16-2, C16-3, C16-4 and C16-5, respectively, from the left end of the chromosome).

Following the confirmation of the reliability of the method and the limitation on the size of the duplicated segment, we attempted to construct a complete library of approximately 200 kb fragments that covered the whole *S. cerevisiae* genome. On the basis of nucleotide sequence information in the *Saccharomyces* Genome Database (SGD) (http://www.yeastgenome.org), we designed primers to amplify duplicating DNA modules that could be used for duplication of approximately 200 kb chromosomal regions of each chromosome in a systematic manner (Fig. 3a). We designated strains with a segmental duplication of a chromosome region as ScDup(Cx-y): Sc represents *S. cerevisiae*; Dup represents duplication; and (Cx-y) indicates chromosome number (Cx) and region (-y). We modified the duplication procedure for the three smallest chromosomes: chromosome I (230 kb), we generated a 100 kb region and a 130 kb region; chromosome III (317 kb), we generated a 158 kb region and a 159 kb region; and for chromosome VI (271 kb), we generated a 100 kb region and a 171 kb region. The chromosomal region containing the ribosomal DNA cluster (ca. 1500 kb) on chromosome XII was not included in this study. The nucleotide positions of each duplicated region and other details are presented in Table 2.

Analyses of the duplicated regions revealed that 53 out of 62 designated regions were duplicated with desired karyotype with a proportion of 3% to 100% of analyzed transformants (Table 2 and Supplementary Fig. 1). The proportion of desired karyotype in analyzed transformants from 31 terminal regions (54% ± 0.24 s.d.) was higher than those from 22 internal chromosomal regions (19% ± 0.23 s.d.). This difference likely reflected the fact that only one homologous recombination event was required for duplication of the terminal regions. Confirmation of the karyotypes of the segmental aneuploids was performed using PFGE and Southern blots; representative data from these analyses for chromosome XVI are shown in Fig. 3b. All of the karyotyping analyses showed the presence of the expected karyotype. A small number of the designated regions did not yield duplicated products: C4-2, C4-4, C4-5, C4-7, C6-1, C7-4, C8-2, C11-2 and C14-2 (Table 2). The possible reason of these results was further analysed in final part of result sections.

### Stability of newly generated chromosomes

To investigate whether the segmental duplicated chromosomes were stable during cell culture, we evaluated the mitotic stability of the strains in comparison to YCp50, a strain carrying a yeast centromere plasmid. We found that YCp50 displayed 85% mitotic stability, whereas the segmentally duplicated chromosomes exhibited almost 100% mitotic stability. These findings indicate that chromosomes derived by PCDup and ranging from 100 kb to 290 kb can be stably maintained (Table 2).

### Effect of stress on growth of segmental aneuploids

Our analysis above showed that strains with segmental aneuploidies were mitotically stable under normal culture conditions. We next examined their growth in stressful environments as this might provide insights into the function of the duplicated region. First, we compared the growth of the 53 segmental aneuploid strains and the parental strain in liquid SC medium. Only one strain, ScDup(C15-4), showed a significant difference in growth when cultured at 30 °C for 24 hours; growth in this strain was slower than the parental strain (Supplementary Fig. 2).

**Figure 4:**
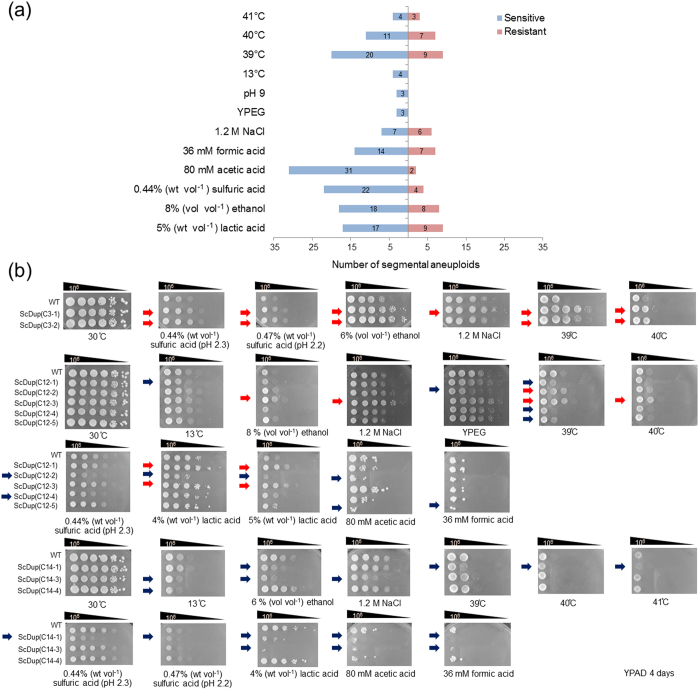
Phenotypic assays of segmental aneuploid strains. (**a**) The numbers of segmental aneuploids that showed increased sensitivity or resistance to each stress condition. Blue bar represents sensitive phenotype and red bar represents resistant phenotype. (**b**) Representative examples of cells grown under different stresses. The growth of segmental aneuploids for chromosomes III, XII and XIV are shown. Ten-fold serial dilutions of segmental aneuploid strains for chromosomes III, XII and XIV were subjected to the indicated stress for 3–4 days. Red arrow represents stress resistant phenotype. Blue arrow represents stress sensitive phenotype.

We then investigated the effects of growing the strains under different challenging conditions: serial dilution assays involving lactic acid (4%, 5% and 6% wt vol^−1^), ethanol (6%, 8% and 10% vol vol^−1^), sulfuric acid (0.41%, 0.44%, 0.47% wt vol^−1^); 80 mM acetic acid, 36 mM formic acid, or 3% glycerol as the carbon source; alkaline pH (pH 9); 1.2 M NaCl; high temperatures (39 °C, 40 °C and 41 °C); and low temperature (13 °C). All but two strains, ScDup(C7-1) and ScDup(C16-3), showed the same colony formation ability as the parental strain when incubated in YPAD at 30 °C (without stress conditions) for 4 days (Supplementary Fig. 3); these two strains displayed slightly slower growth than the parental strain when incubated for 1 day (Supplementary Fig. 3g and 3p) although they showed normal growth when incubated for 4 days (Supplementary Fig. 3). However, we identified differences when we compared the growth of the segmental aneuploid strains with their parental wild-type strain under different stress conditions (Supplementary Fig. 3a–p and Supplementary Tables 1 and 2). The number of strains classified as sensitive or resistant to each stress condition is shown in Fig. 4a and representative examples of spot assays under these stress conditions for 10 segmental aneuploid strains showing sensitive or resistance phenotypes are presented in Fig. 4b. The results for the spot assays from all 53 segmental aneuploid strains and each of the 18 stress conditions are given in Supplementary Fig. 3. The strains that showed significantly more sensitive or resistant phenotypes compared to the parental strain are identified in Table 3. Our analyses indicated that all segmental aneuploid strains except for ScDup(C10-4) showed a different pattern of response to at least one tested stress compared to the parental strain. Although most of the segmental aneuploidy strains showed stress sensitivity, interestingly, only a few showed increased tolerance of thermal stress, high concentrations of ethanol, acidic conditions or osmotic stress (Table 3, Supplementary Fig. 3). We found that segmental aneuploid strains such as ScDup(C2-3), ScDup(C3-1), ScDup(C3-2), ScDup(C5-3), SCDup(C7-5), ScDup(C12-3), ScDup(C15-2), ScDup(C15-3), ScDup(C16-2) and ScDup(C16-4) showed increased tolerance to multiple stresses. Based on SGD database, we searched genes among those located on these duplicated regions that are required for those stress resistance and found that those chromosomal regions contained several specific genes that may be concerned with resistance against each stress. We also noted that some genes might have conferred tolerance to more than one particular stress (See details in discussion section). Therefore, duplication of specific chromosomal region**s** might offer a means for cells to survive under unfavourable conditions.

### Association of phenotypic changes with segmental aneuploidy

**Figure 5:**
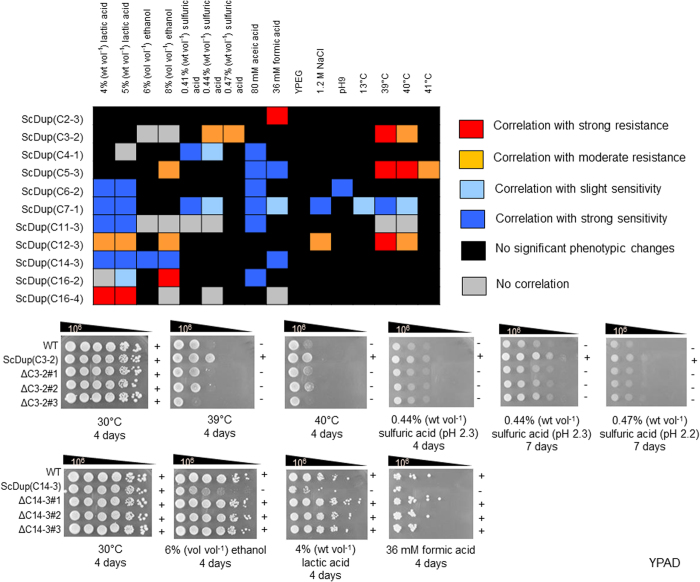
Relationship between segmental duplication of a particular region and phenotype. Effect of loss of the segmentally duplicated chromosome on phenotype. The correlation of phenotypic changes in aneuploids and the presence of a duplicated region is illustrated: red squares, orange squares, light blue and dark blue squares indicate correlation with strongly resistant phenotype, moderately resistant phenotype, slightly sensitive phenotype and strongly sensitive phenotype, respectively. Gray squares represents no correlation of observed phenotype and duplicated chromosome. Black square indicate stress conditions that were not tested as the segmental aneuploid did not show significant growth or other changes compared to the parental strain at the initial phenotypic examination step. Spot assays of ScDup(C3-2), ScDup(C14-3) and their derivatives are shown as representative examples. “+” and “−” indicate resistant and sensitive phenotypes, respectively.

To confirm that the changes in phenotype in segmental aneuploids were the result of the duplicated chromosomal segments, we investigated whether removing the additional chromosome caused a reversion to the parental phenotype (Fig. 5, Supplementary Figs 4 and 5). We arbitrarily selected 11 segmental aneuploids, ScDup(C2-3), ScDup(C3-2), ScDup(C4-1), ScDup(C5-3), ScDup(C6-2), ScDup(C7-1), ScDup(C11-3), ScDup(C12-3), ScDup(C14-3), ScDup(C16-2), and ScDup(C16-4), and subjected them to stress assays after removal of the duplicated chromosome. A total of 60 assays were performed with these modified strains and, in 47 cases, removal of the duplicated chromosome resulted in reversion to the parental phenotype. In these segmental aneuploid strains, therefore, the phenotypic changes were caused by the presence of the duplicated region. However, in some assays involving ScDup(C3-2), ScDup(C4-1), ScDup(C11-3), ScDup(C16-2), and ScDup(16-4) (13 of the 60 tests), it was clear that removal of the additional chromosome did not result in reversion to the parental phenotype indicating that the phenotypes of these segmental aneuploid strains did not show a clear association with the presence of the duplicated region (Fig. 5). Thus, in some cases, the phenotypes may not be due to the segmentally duplicated chromosome.

In the 53 segmental aneuploids constructed in this study, we noted that only 5 duplicated regions, C3-1, C3-2, C5-3, C12-3 and C15-3, harbored genes based on published data of single-gene overexpression, which confer sensitivity or resistance to a tested stress^33^,^34^,^35^,^36^,^37^,^38^ (see Discussion section). Therefore, the phenotypic changes in these segmental aneuploids could be interpreted as being the result of increased expression of particular genes. Interestingly, however, although the strains harboring the other 48 duplicated regions displayed phenotypic changes to stress, the duplicated regions did not contain genes whose overexpression caused the respective change to the tested stress. This suggests that for these 48 regions, an increased dosage of multiple genes might be responsible for the change of phenotype.

### Unidentified genes or gene-pairs prevent duplication

Interestingly, nine of the designated 62 regions of approximately 200 kb could not be duplicated, namely, C4-2, C4-4, C4-5, C4-7, C6-1, C7-4, C8-2, C11-2 and C14-2. To explore the reason for this effect, we attempted to duplicate these regions after dividing each into 50 kb sub-regions. For C4-5 and C7-4, all 50 kb sub-regions could be duplicated, suggesting that interaction of multiple genes on different 50 kb regions might have prevented duplication of the intact 200 kb regions. However, for the remaining seven regions, it was not possible to duplicate one of the four 50 kb sub-regions although the other sub-regions were duplicated. We designated these 50 kb unduplicated regions as C4-2-S4, C4-4-S2, C4-7-S4, C6-1-S2, C8-2-S3, C11-2-S2, and C14-2-S4 (Table 4). Based on SGD database, with the exception of C6-1-S2, the 50 kb unduplicated regions did not contain an ARS. It is possible that the duplicating modules did not recombine with its target region but freely replicated in the cell because the duplicating modules in this experiment were prepared by incorporating *H4ARS* with *CgHIS3* and telomere seed sequences. Therefore, we investigated whether a duplicating module with an additional *H4ARS* could recombine with the target site; we attempted to generate C7-4-S4 duplicates that contain an ARS using duplicating modules with *H4ARS*. We found that C7-4-S4 could be duplicated even when using duplicating modules with *H4ARS* which means that the duplicating module recombined with the target region despite of the presence of ARS element. Next, we attempted to construct strains with duplication of a 100 kb sub-region, consisted of the 50 kb duplicatable region harboring the resident *ARS* and the adjacent 50 kb unduplicatable region without an *ARS*. These 100 kb sub-regions, designated C4-2-(S3+S4), C4-4-(S2+S3), C4-7-(S3+S4), C8-2-(S3+S4), C11-2-(S1+S2) and C14-2-(S3+S4), could not be duplicated, suggesting that the 50 kb unduplicatable sub-region inhibited duplication of the 100 kb sub-region (Table 4). These results could be explained if the 50 kb unduplicatable region contained a gene or gene-pairs that induce cell lethality when they are duplicated.

## Discussion

Here, we developed a novel technology, termed PCDup, for engineering the yeast genome to generate cells with segmental aneuploidy through a single transformation step. These cells harbor a normal haploid genome and an extra chromosome consisting of a specific chromosomal region at a designated site. Using this technology, duplication of chromosomal regions up to 300 kb could be generated efficiently. In this study, we used our new technology to produce a set of approximately 200 kb overlapping duplicated regions that covered most of the 16 chromosomes of *S. cerevisiae*. We carried out an initial investigation of the phenotypic changes resulting from each of these segmental aneuploidies. A small number of regions in the genome could not be duplicated possibly because they contained genes or gene pairs that cause cell lethality when they are duplicated. It should be noted that methodology similar to PCDup has not previously been developed in any other organism.

**Figure 6:**
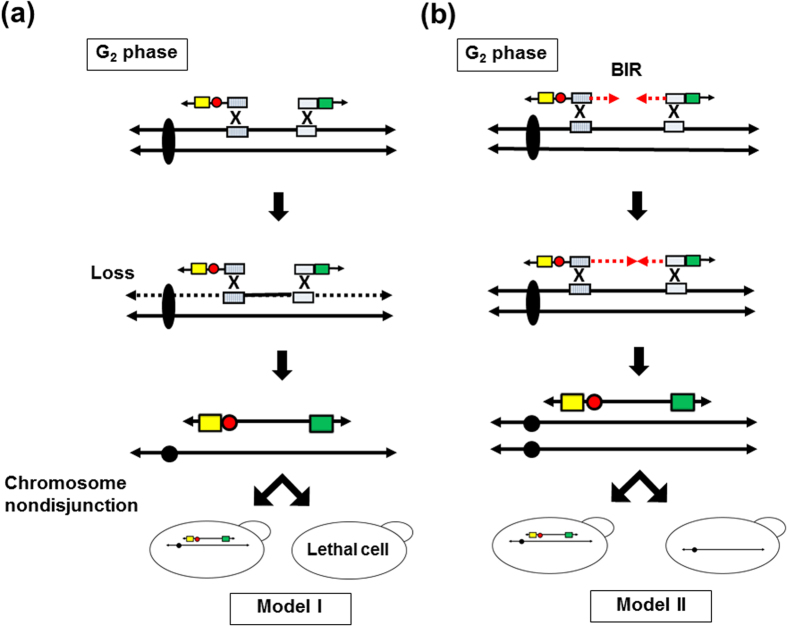
Possible mechanisms for generation of segmentally duplicated chromosomes. In model I, each of the two duplicating modules is assumed to recombine with two target regions on the same sister chromatid. The target region is then generated as a new chromosome. Sequences outside the target region are lost during mitotic cell division due to the lack of centromere or telomere. If chromosome nondisjunction happens, either the daughter cell or mother cell is expected to have both the targeted natural chromosome and the newly generated segmentally duplicated chromosome, while the remaining cell loses its chromosome. Model II is based on the BIR mechanism. In this model, the duplicating module is expected to invade the target chromosome and initiate DNA synthesis from the homologous site of one duplicating module to the homologous site of the other duplicating module. This action generates the segmentally duplicated chromosome.

Two possible mechanisms might explain how segmentally duplicated chromosomes are generated by PCDup. In the first model (Fig. 6a), the duplicating modules recombine with each of their target sites. The regions outside the target area are lost due to the lack of a centromere or telomere. The duplicated chromosome is then generated. Our results indicated that 300 kb was an upper limit to the size of the chromosome region that could be duplicated. This effect may be related to the fact that the rate of chromosome nondisjunction is correlated with linear chromosome length^39^. Therefore, in the first model (Fig. 6a), chromosome nondisjunction would be expected to occur more frequently for smaller derived chromosomes. The upper size limitation of chromosome duplication here of approximately 300 kb might be determined by the low likelihood of nondisjunction of these de novo chromosomes.

The second possible mechanism (Fig. 6b) is based on the Break Induced Replication (BIR) model^40^,^41^. The distance between two homologous sites is one of the parameters of the recombination execution checkpoint (REC) that regulates the choice of homologous recombination pathway during double strand break (DSB) repair (gene conversion, single-strand annealing or BIR). The signals for the initiation of new DNA synthesis between DSB ends are lost when the distance between two homologous sites increases. If the distance is greater than 5 kb, the mode of gap repair shifts from gene conversion to BIR^42^. The frequency of BIR depends on the length of template. When the distance is large, complete BIR synthesis is likely limited by the requirement in chromatin remodeling for migration of the D-loop and initiation of lagging strand synthesis^43^. Morrow *et al*. claimed that they could observe duplication events generated by the “break copy” mechanism of up to 365 kb^40^. Therefore, another explanation for the upper size limit of segmentally duplicated chromosomes here is a defect in completion of DNA synthesis due to the increased distance between homologous sites (Fig. 6b)^43^.

Interestingly, we found that only the C4-2-S4 region, of the seven 50 kb sub-regions that could not be duplicated, did not contain any gene that might cause cell lethality when it is duplicated. We suggest that the influence of two or multiple genes in the C4-2-S4 sub-region prevented duplication of the 200 kb region. In the other 6 sub-regions, we suggest the presence of genes that caused a decrease in cell viability upon duplication. For example, the C6-1-S2 region carries *TUB2* and it has been shown that additional copies of *TUB2* cause cell lethality^44^. Likewise, the C4-4-S2, C4-7-S4, C8-2-S3, C11-2-S2 and C14-2-S4 sub-regions harbor one to four genes that cause cell lethality^45^,^46^, toxicity^47^, or abnormal cell-cycle progression^48^,^49^ when overexpressed (Supplementary Table 3). Although, these genes may be the cause of severe cell growth defects, there is other evidence that argues against this conclusion. In the reports showing adverse effects, these genes were overexpressed under the control of a strong inducible *GAL1* promoter and/or expressed in multi-copies. However, in the segmental aneuploid strains here, the genes are regulated by the endogenous promoter with two or three copies at most. Moreover, Makanae *et al*.^50^ catalogued the lowest number of copies of each *S. cerevisiae* gene that caused cell lethality when expressed under the native promoter. On the basis of their data, we examined the genetic contents of the unduplicatable regions and found that none of the 50 kb sub-regions contained genes that have a severe defect on cell growth when present as two or three copies (Supplementary Table 4). Therefore, we conclude that combinatorial duplication of two or more genes in these sub-regions might be responsible for cell lethality, which prevents duplication of the regions.

In many organisms, aneuploidy is associated with defects in growth and the extent of this detrimental effect is proportional to the number of extra genes present in the aneuploid cells^51^. Yeast is generally more tolerant of aneuploidy compared to multicellular organisms. Since all but one of the segmental aneuploid strains did not show any effect on growth when cultured in liquid SC medium at 30 °C for 24 hours, then it appears that the additional genes did not influence proliferation. This conclusion is supported by the results of a previous study^27^ in which it was found that cells with whole chromosome aneuploidies generally show a delay in cell division that is proportional to the number of genes located on the additional chromosome, although disomy for chromosome I (230 kb) does not cause a proliferation delay relative to the euploid genome. The sizes of the segmentally duplicated chromosomes constructed in this study were in the range 100 to 290 kb, and comparable to chromosome I. We suspect, therefore, that the segmental aneuploid strains in this study would not show severe growth defects under non-stressful conditions compared to the parental strain, as their gene dosage imbalance would be similar to or less than that of aneuploidy for chromosome I. However, the growth delay in ScDup(C15-4) might have resulted from the presence of genes whose over-expression interferes with cell proliferation.

The phenotypic changes seen in aneuploids are due to the increased copy numbers of either single genes or multiples of genes^2^,^3^,^5^,^9^,^20^,^26^,^30^. We suggest that most of the phenotypic changes found here were caused by multiple-gene effects rather than by single genes (Supplementary Fig. 3 and Supplementary Table 5). This suggestion is based on the fact that only a few of the duplicated regions that conferred sensitivity or resistance to environmental stresses actually contained single genes whose overexpression might cause such phenotypic alteration. These latter exceptions were *SAT4*^34^ on C3-1 region and *RSA3*^33^ on C12-3 region that confer high salt tolerance, *SPT15*^35^ on C5-3 and *RSA3*^33^ on C12-3 region that confer ethanol resistance, and *LRE1*^37^ on C3-1, *HCM1*^38^ on C3-2 and *LSP1*^36^ on C15-3 that confer thermotolerance. Moreover, we noted that several segmental aneuploids revealed tolerance to multiple stresses (Table 3 and Fig. 4) and by scrutinizing SGD database, we found that some of the duplicated regions contains more than one gene that play a role in resistance to those stresses. For example, ScDup(C12-3) exhibited resistance to ethanol, high salt concentration, lactic acid and high temperature and we found that the duplicated region harbors several specific genes that are essential for tolerance to those stresses as genes whose deletion causes increased susceptibility to each stress. Based upon this information, we recognized that several genes seem to be responsible for resistance to more than one particular stress. For example, *VPS34* is required for resistance to high salt, high lactic acid and high temperature, *VPS63* is essential for tolerance against high ethanol, high lactic acid and high temperature, *LCB5, LIP2, MSS51, QRI5* and *SWI6* are responsible for ethanol resistance and thermotolerance. *YLR194C* is required for ethanol stress and high salt stress resistance. *DCS1* and *MAP1* are essential for high salt and thermal stress tolerance. *BUR2* and *YPT6* are responsible for resistance to lactic acid and heat stress. These facts suggested that multiple stress resistance observed in those segmental aneuploids might be conferred by the combination of increased dosage of several numbers of individual genes that are required for each particular stress resistance and duplication of gene that is responsible for multiple stress tolerance. However, since increased low dosages (from one copy to two copies) of a single specific gene located in those duplicated regions is not reported to cause multiple phenotypic alterations that were observed in this study, we think that duplication of only single specific gene is unlikely to cause those observed phenotypic changes but rather suggest that the combined effect that could result from simultaneously increased dosage of multiple genes in duplicated region conferred those observed sensitivity and resistance. Upon these observations, it should be emphasized that generating segmental aneuploidy with desired manner could be beneficial approach to study the consequence of change in dosage of multiple genes within contiguous region and to identify possible underlying genes involved in such phenotypic alterations.

In 11 arbitrarily selected strains, removal of the duplicated chromosome resulted in reversion to the parental phenotype in the majority of cases when subjected to a stress (47 out of 60 assays; Fig. 5). However, in a few cases, the phenotypes of the segmental aneuploid strains did not appear to be correlated with the duplicated chromosome. We envisage two possible explanations for this effect. First, the duplicated chromosome in the derivative strain might have recombined with the intact chromosome at a homologous or ectopic site and generated a chromosome rearrangement, such as translocation, which would make any linkage between phenotypic change and the segmentally duplicated chromosome unclear. Second, unknown mutations might have occurred by chance in the segmental aneuploid; however, the possibility that a combined effect of the presence of a segmentally duplicated region and unknown mutations is responsible for the phenotype cannot be excluded.

We noted that some segmental aneuploid strains, such as ScDup(C2-3), ScDup(C3-2), ScDup(C5-3), ScDup(C12-3), ScDup(C16-2) and ScDup(16-4) (Fig. 5 and Supplementary Fig. 4), enhanced simultaneous tolerance to several types of stress. If this proves to be a consistent feature of the strains generated by PCDup, then this could be exploited as a breeding tool to generate superior strains that have desirable industrial phenotypes. It has been reported that segmental duplication may play an important role in the emergence of stress resistance in yeasts growing in unfavorable environments^9^,^20^,^22^. Through integration of the information on spontaneous genome rearrangements in natural and laboratory populations of yeast, i.e., the precisely induced segmental duplication constructed by PCDup technology in the latter populations, we will be able to improve our understanding of the biological significance of segmental duplication as an adaptive mechanism in the evolution of the *S. cerevisiae* genome. Where duplication of particular whole chromosomes produces phenotypic changes, then PCDup technology could be used to identify the exact region that generates the specific phenotype. It should be emphasized that our new collection of *S. cerevisiae* haploid yeast strains with controlled duplication of specific chromosomal regions will be a valuable resource for studying the association of segmental aneuploidy with particular traits. These strains should help to accelerate research on gene dosage balance and the effects of simultaneously increased dosages of multiple genes.

Many genetic disorders and cancers in humans are associated with segmental duplication^6^,^7^,^10^,^11^,^12^,^13^,^14^,^17^,^19^. However, the relationship between these specific segmental duplications and their phenotypic consequences are not fully understood. Our development of a technology to generate specific segmental aneuploids in a model organism is a starting point to explore gene(s) or genomic regions that are responsible for pathogenesis and diseases in higher organisms including humans. As demonstrated in this study, segmental aneuploidy occasionally improves the tolerance of cells to stress. This observation suggests that aneuploidy or segmental aneuploidy might enable cancer cells to adapt to extreme conditions^52^. Information on segmental aneuploidy obtained from our yeast model may improve our basic understanding of the molecular mechanisms of segmental aneuploidy-derived human diseases and cancer.

In conclusion, the PCDup method is a simple, efficient, rapid, and economic genetic tool for generating segmental aneuploidy at any selected region of a chromosome in *S. cerevisiae*. It can be used as a technique not only for studying genome function but also breeding novel strains with desired properties for industrial purposes.

## Methods

### Yeast strains and plasmids

*Saccharomyces cerevisiae* strain BY4742 [*MAT*α *his3∆1 leu2∆0 lys2∆0 ura3∆0*] was used as the parental strain for the construction of segmental aneuploid strains. The plasmids used in this study are listed in Supplementary Table 6. Yeast cells were grown at 30 °C in YPAD medium [5% (wt vol^−1^) YPD (Difco^TM^) and 0.04% (wt vol^−1^) adenine (Wako)] or synthetic complete (SC) medium^53^. *E. coli* strains were grown at 37 °C in LB medium (Sigma) with or without 75 μg ml^−1^ ampicillin (Wako). Plasmid DNA was isolated from *Escherichia coli* strains according to the alkaline lysis method^54^.

### PCR-mediated chromosome duplication method (PCDup)

The primers used in this study are listed in Supplementary Tables 7 to 9. The *Saccharomyces* Genome Database (http://www.yeastgenome.org) was used to select the target region for duplication and to design primers. The two DNA modules required for PCDup were prepared by two rounds of PCR. In the first round of PCR, loxP-cas and CA primers were used to amplify a DNA fragment from plasmid template (Supplementary Table 6). Two DNA cassettes were amplified from the plasmids: one contained the telomere seed sequences, selectable marker and *CEN4* (fragment 1); the other contained the telomere seed sequences and a second selectable marker (fragment 2). In parallel, two DNA fragments (400 bp; fragments 3 and 4) with nucleotide sequences corresponding to the left and right ends of the target region were amplified from genomic DNA of strain BY4742. One pair of primers designated Cx-y-L-f and Cx-y-L-r and a second pair designated Cx-y-R-f and Cx-y-R-r were used to amplify DNA fragments at the left and right ends of the target region, respectively (Supplementary Table 7; x represents chromosome number, y represents chromosome region, L represents left end of target region, R represents right end of target region, f represents forward primer, and r represents reverse primer). The Cx-y-L-f and Cx-y-L-r primers contained 20 bp sequences that respectively corresponded to the 5′ and 3′ ends of the fragment at the left end of the target region; the Cx-y-R-f and Cx-y-R-r primers likewise contained 20 bp sequences corresponding to the 5′ and 3′ ends of the fragment at the right end. In addition, the Cx-y-L-r and Cx-y-R-f primers also contained 30 bp annealing sequences complementary to the DNA fragment amplified from the plasmid to further amplify the duplicating module in the next step of PCR. After the first round of PCR, the 4 PCR products (fragments 1–4) were gel-purified using a Wizard SV Gel and PCR Clean-up System (Promega).

Next, overlap extension PCR was performed to amplify the two duplicating DNA modules: one target fragment (fragment 3 or 4) was combined with a marker cassette (fragment 1 or 2) by overlap extension PCR using primers Cx-y-L-r and CA, or primers Cx-y-R-f and CA. After amplification, the two PCR products were ethanol-precipitated.

The first round of PCR was performed using 1.0 U *Ex Taq* DNA Polymerase (Takara), approximately 50 ng of DNA template and 0.1 μM of each primer in a final volume of 50 μl. The overlap extension PCR was performed using a final volume of 100 μl containing an equal amount of PCR product from the plasmid and genomic DNA, 2.0 U *Ex Taq* DNA Polymerase (Takara) and 1 μM of each primer. The following PCR cycle was used: 94 °C for 5 min; 30 cycles of 94 °C for 30 seconds, 55 °C for 30 seconds, and extension step at 72 °C for an appropriate time; and 72 °C for 7 min. All PCR amplifications were carried out on a Gene Amp PCR System 9700 (Applied Biosystems).

### Yeast transformation

Yeast cells were transformed according to the method of Gietz and Schiestl^55^. For selection of yeast transformants, cells were cultured on SC medium without leucine, or without leucine and histidine, or without leucine and uracil at 30 °C for 4 days.

### Karyotype analysis by PFGE and Southern blot analysis

PFGE and Southern blot analysis were performed according to Sugiyama *et al*. (2005)^56^. Chromosomes were separated on 1% (wt vol^−1^) pulsed-field gel electrophoresis gels in 0.5× TBE (Tris-borate-EDTA) buffer at 14 °C using the CHEF DRIII® System (Bio-Rad Laboratories), with a 60 s pulse for 15 hours, followed by a 90 s pulse for 9 hours, at 6 V cm^−1^. The specific probes for Southern blot analysis were amplified by the primers listed in Supplementary Tables 10–12.

### Mitotic stability of segmentally duplicated chromosomes

Yeast cells were cultured in 5 ml of YPAD medium at 30 °C overnight and the optical density was then measured at 660 nm (OD_660_). Cell cultures were transferred into 5 ml of fresh YPAD media at an initial OD_660_ of 0.1. After incubation at 30 °C for 24 hours, cell culture was measured at OD_660_ and the culture was diluted to a concentration of 1 × 10^3^ cells ml^−1^. About 100–200 cells were spread on each of three YPAD plates and incubated at 30 °C for 24 hours, before being replicated onto YPAD and selective media plates. After incubation at 30 °C for 24 hours, colony numbers on the plates were counted and % mitotic stability was calculated by the following equation:


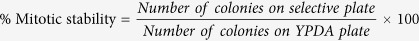


### Phenotypic analysis under stress conditions

Yeast cells were cultured in appropriate selective media overnight at 30 °C. Next day, aliquots of the cell cultures were transferred into fresh selective media and incubated at 30 °C until the culture reached the log phase. The cells were then harvested, re-suspended in sterile water, diluted to a concentration of 0.25 × 10^6^ cells μl^−1^ and further serially diluted by 1:10. After that, 4 μl aliquots of each cell dilution was spotted onto different plates: YPAD medium supplemented with 4% (wt vol^−1^), 5% (wt vol^−1^) and 6% (wt vol^−1^) lactic acid (pH 2.8, pH 2.7 and pH 2.6, respectively), 4% (vol vol^−1^), 6% (vol vol^−1^) and 8% (vol vol^−1^) ethanol, 0.41% (wt vol^−1^), 0.44% (wt vol^−1^) and 0.47% (wt vol^−1^) sulfuric acid (pH 2.4, pH 2.3 and pH 2.2, respectively), 36 mM formic acid (pH 4.0), 80 mM acetic acid (pH 4.2), 1.2 M NaCl, pH 9 (adjusted by NaOH) and YPA (1% (wt vol^−1^) yeast extract, 2% (wt vol^−1^) bacto peptone and 0.04% (wt vol^−1^) adenine) with 3% (vol vol^−1^) glycerol (YPEG). The plates were incubated at 30 °C. For the temperature stress experiment, cells were incubated on YPAD medium at 13 °C, 30 °C, 39 °C, 40 °C and 41 °C. All plates were incubated for 3–4 days and photographed. Three replicates were carried out for each experiment.

### Elimination of the segmentally duplicated chromosome

Yeast strains were cultured in YPAD medium at 39 °C for 24 hours and then transferred into fresh medium at an initial OD_660_ of 0.1 followed by culture at 30 °C for 24 hours. Approximately 100–200 cells from each cell culture were spread on ten plates of YPAD medium. After incubation at 30 °C for 48 hours, the cells were replica plated onto YPAD and appropriate selective media to observe chromosome loss. Colonies that failed to grow on selective media lacking leucine and/or histidine were expected to be those with loss of the segmentally duplicated chromosome during mitotic growth. After confirmation of loss of the segmentally duplicated chromosome by PFGE, serial dilution spot assays were performed to investigate the phenotypes of the segmental aneuploids and the derived strains with loss of the segmentally duplicated chromosome.

## Additional Information

**How to cite this article**: Natesuntorn, W. *et al*. Genome-wide construction of a series of designed segmental aneuploids in *Saccharomyces cerevisiae*. *Sci. Rep*. **5**, 12510; doi: 10.1038/srep12510 (2015).

## Supplementary Material

Supplementary Information

## Figures and Tables

**Table 1:** Characteristics of segmental aneuploids of chromosomes I, II, IV, VIII and X.

Duplicated region^a^	Duplication length (kb)	Plasmid template^b^	Transformants (n)	Proportion of desired karyotype^c^	% Mitotic stability
Chr. I 37,504–87,735	50	p3122, p3276	55	30.00% (3/10)	100%
Chr. II 360,775–505,293	145	p3122, p3276	11	10.00% (1/10)	100%
Chr. IV 148,203–401,638	250	p3009, p3122	31	7.69% (1/13)	99%
Chr. IV 97,475–401,638	300	p3009, p3122	44	6.25% (1/16)	100%
Chr. IV 50,000–401,638	350	p3009, p3122	39	0.00% (0/39)	ND^d^
Chr. IV 198,996–600,688	400	p3009, p3122	11	0.00% (0/11)	ND^d^
Chr. VIII 294,748– 346,028	50	p3122, p3276	18	21.43% (2/14)	100%
Chr. VIII 247,693–346,028	100	p3122, p3276	34	10.00% (1/10)	100%
Chr. VIII 192,203–346,028	150	p3122, p3276	32	10.00% (1/10)	100%
Chr. VIII 145,656–346,028	200	p3122, p3276	6	33.33% (2/6)	100%
Chr. X 225,115–326,063	100	p3122, p3276	18	20.00% (2/10)	100%
YCp50 (7.8 kb)	–	YCp50	NC^d^	NC^d^	85%

^a^Chr. N x-y: Chr. N represents chromosome number, x represents first nucleotide number of chromosomal region and y represents last nucleotide number of chromosomal region.

^b^p3009 was used to amplify the *CgHIS3* cassette, p3122 was used to amplify the *CEN4-CgLEU2* cassette, p3276 was used to amplify the *URA3* cassette, p3279 was used to amplify the *CgHIS3-H4ARS* cassette and YCp50 was a *URA3* centromeric plasmid whose length was 7.8 kb.

^c^Proportion of desired karyotype in analyzed transformants (number of segmental aneuploids/number of candidate transformants that were analyzed for karyotype).

^d^ND means no data. NC means not checked.

**Table 2:** Characteristics of a complete collection of overlapping segmental aneuploids of chromosomes I to XVI.

Region	Strain name	Duplicated region^a^	Plasmid template^b^	Duplication length (kb)	Number of genes	Transformants (n)	Proportion of desired karyotype^c^	% Mitotic stability
C1-1	ScDup(C1-1)	Chr. I 1–100,705	p3122	100	65	7	71% (5/7)	98%
C1-2	ScDup(C1-2)	Chr. I 99,603–230,218	p3008	130	85	16	50% (8/16)	99%
C2-1	ScDup(C2-1)	Chr. II 1–202,750	p3122	200	137	70	67% (6/9)	98.57%
C2-2	ScDup(C2-2)	Chr. II 201,029–401,862	p3008, p3009	200	128	6	17% (1/6)	100%
C2-3	ScDup(C2-3)	Chr. II 400,204–600,988	p3009, p3122	200	124	25	5% (1/22)	99.79%
C2-4	ScDup(C2-4)	Chr. II 599,536–813,184	p3122	213	142	29	100% (9/9)	100%
C3-1	ScDup(C3-1)	Chr. III 1–158,020	p3008	158	139	4	75% (3/4)	99%
C3-2	ScDup(C3-2)	Chr. III 157,543–316,620	p3122	159	110	5	20% (1/5)	100%
C4-1	ScDup(C4-1)	Chr. IV 1–200,732	p3122	200	119	56	78% (7/9)	97.70%
C4-2#	ScDup(C4-2)	Chr. IV 198,996–401,638	p3009, p3122	200	128	219	0% (0/219)	ND
C4-3	ScDup(C4-3)	Chr. IV 399,987–600,688	p3008, p3009	200	140	5	20% (1/5)	100%
C4-4#	ScDup(C4-4)	Chr. IV 599,793–795,723	p3009, p3122	200	114	134	0% (0/134)	ND
C4-5#	ScDup(C4-5)	Chr. IV 795,193–1,000,877	p3009, p3122	200	133	22	0% (0/22)	ND
C4-6	ScDup(C4-6)	Chr. IV 999,134–1,199,697	p3009, p3122	200	121	13	8% (1/13)	99.56%
C4-7#	ScDup(C4-7)	Chr. IV 1,198,183–1,402,247	p3009, p3122	200	134	27	0%(0/27)	ND
C4-8	ScDup(C4-8)	Chr. IV 1,400,770–1,531,933	p3122	130	89	41	89% (8/9)	100%
C5-1	ScDup(C5-1)	Chr. V 1–199,519	p3008	200	146	17	80% (8/10)	100%
C5-2	ScDup(C5-2)	Chr. V 197,812–400,060	p3009, p3122	200	143	5	20% (1/5)	99.77%
C5-3	ScDup(C5-3)	Chr. V 398,496–576,874	p3122	177	127	5	20% (1/5)	100%
C6-1#	ScDup(C6-1)	Chr. VI 1–98,498	p3122	100	57	24	0% (0/24)	ND
C6-2	ScDup(C6-2)	Chr. VI 98,213–270,161	p3008	171	128	8	50% (4/8)	100%
C7-1	ScDup(C7-1)	Chr. VII 1–201,147	p3122	200	125	14	57% (8/14)	100%
C7-2	ScDup(C7-2)	Chr. VII 199,564–398,642	p3009, p3122	200	128	3	67% (2/3)	97.45%
C7-3	ScDup(C7-3)	Chr. VII 397,621–599,626	p3008, p3009	200	154	15	7% (1/15)	100%
C7-4#	ScDup(C7-4)	Chr. VII 598,443–801,057	p3009, p3122	200	133	156	0% (0/156)	ND
C7-5	ScDup(C7-5)	Chr. VII 799,553–1,090,940	p3122	290	181	10	60% (6/10)	100%
C8-1	ScDup(C8-1)	Chr. VIII 1–202,241	p3008	200	146	22	44% (4/9)	100%
C8-2#	ScDup(C8-2)	Chr. VIII 203,559–401,907	p3009, p3122	200	140	72	0% (0/72)	ND
C8-3	ScDup(C8-3)	Chr. VIII 400,443–562,643	p3122	160	99	27	44% (4/9)	100%
C9-1	ScDup(C9-1)	Chr. IX 1–203,042	p3122	200	116	31	78% (7/9)	99.68%
C9-2	ScDup(C9-2)	Chr. IX 201,284–439,888	p3008	240	175	11	56% (5/9)	100%
C10-1	ScDup(C10-1)	Chr. X 1–195,892	p3122	200	131	7	29% (2/7)	100%
C10-2	ScDup(C10-2)	Chr. X 195,298–403,454	p3009, p3122	200	130	18	11% (2/18)	100%
C10-3	ScDup(C10-3)	Chr. X 401,881–599,357	p3008, p3009	200	142	6	17% (1/6)	100%
C10-4	ScDup(C10-4)	Chr. X 597,731–745,751	p3122	150	87	12	67% (8/12)	100%
C11-1	ScDup(C11-1)	Chr. XI 1–201,168	p3009, p3122	200	116	6	50% (3/6)	100%
C11-2#	ScDup(C11-2)	Chr. XI 199,892–399,750	p3009, p3122	200	133	202	0% (0/100)	ND
C11-3	ScDup(C11-3)	Chr. XI 397,819–666,816	p3008	267	153	58	90% (9/10)	100%
C12-1	ScDup(C12-1)	Chr. XII 1–251,980	p3008	250	146	20	10% (2/20)	99.89%
C12-2	ScDup(C12-2)	Chr. XII 250,272–450,039	p3009, p3122	200	117	9	11% (1/9)	100%
C12-3	ScDup(C12-3)	Chr. XII 490,862–692,029	p3009, p3122	200	140	11	9% (1/11)	100%
C12-4	ScDup(C12-4)	Chr. XII 690,555–885,764	p3009, p3122	200	139	34	10% (1/10)	99.03%
C12-5	ScDup(C12-5)	Chr. XII 884,258–1,078,177	p3122	200	115	73	70% (7/10)	100%
C13-1	ScDup(C13-1)	Chr. XIII 1–204,690	p3122	200	130	5	20% (1/5)	100%
C13-2	ScDup(C13-2)	Chr. XIII 203,398–402,207	p3008, p3009	200	141	1	100% (1/1)	99.04%
C13-3	ScDup(C13-3)	Chr. XIII 400,538–600,143	p3009, p3122	200	133	33	3% (1/29)	82.02%
C13-4	ScDup(C13-4)	Chr. XIII 598,338–798,915	p3009, p3122	200	120	11	9% (1/11)	100%
C13-5	ScDup(C13-5)	Chr. XIII 797,512–924,441	p3122	120	83	29	60% (6/10)	98.91%
C14-1	ScDup(C14-1)	Chr. XIV 1–200,971	p3122	200	122	21	43% (9/21)	96.67%
C14-2#	ScDup(C14-2)	Chr. XIV 199,575–403,514	p3009, p3122	200	132	152	0% (0/152)	ND
C14-3	ScDup(C14-3)	Chr. XIV 401,690–598,530	p3009, p3122	200	130	29	3% (1/29)	99.08%
C14-4	ScDup(C14-4)	Chr. XIV 597,394–784,333	p3008	184	118	7	14% (1/7)	100%
C15-1	ScDup(C15-1)	Chr. XV 1–201,315	p3122	200	125	20	56% (5/9)	99.87%
C15-2	ScDup(C15-2)	Chr. XV 199,377–401,104	p3008, p3009	200	135	17	6% (1/17)	100%
C15-3	ScDup(C15-3)	Chr. XV 399,345–603,357	p3009, p3122	200	128	16	6% (1/16)	99%
C15-4	ScDup(C15-4)	Chr. XV 601,731–801,721	p3009, p3122	200	134	9	11% (1/9)	84.76%
C15-5	ScDup(C15-5)	Chr. XV 799,959–1,091,289	p3122	290	176	67	56% (5/9)	99.45%
C16-1	ScDup(C16-1)	Chr. XVI 1–198,780	p3122	200	124	39	44% (4/9)	99.71%
C16-2	ScDup(C16-2)	Chr. XVI 198,090–399,110	p3009, p3122	200	116	6	17% (1/6)	100%
C16-3	ScDup(C16-3)	Chr. XVI 397,495–597,301	p3008, p3009	200	124	8	13% (1/8)	100%
C16-4	ScDup(C16-4)	Chr. XVI 595,746–799,875	p3009, p3122	200	136	6	17% (1/6)	99.76%
C16-5	ScDup(C16-5)	Chr. XVI 798,248–948,066	p3122	148	112	46	26% (5/19)	100%
YCp50 (7.8 kb)				–				85%

^a^Chr. N x-y: Chr. N represents chromosome number, x represents first nucleotide number of chromosomal region and y represents last nucleotide number of chromosomal region.

^b^p3009 was used to amplify the *CgHIS3* cassette, p3122 was used to amplify the *CEN4-CgLEU2* cassette, p3008 was used to amplify the *CgLEU2* cassette and YCp50 was a *URA3* centromeric plasmid whose length was 7.8 kb.

^c^Proportion of desired karyotype in analyzed transformants (number of segmental aneuploids/number of candidate transformants that were analyzed for karyotype)

^#^means region that could not be duplicated.

ND means not determined

**Table 3:** Segmental aneuploid strains that showed moderate or strong changes in phenotype against the tested stresses.

Stress conditions	Sensitive phenotype	Resistant Phenotype
4% and 5% (wt vol^−1^) lactic acid	ScDup(C1-1), ScDup(C2-4), ScDup(C14-1), ScDup(C14-3), ScDup(C16-1), ScDup(C16-2), ScDup(C16-3) and ScDup(C16-5)	ScDup(C4-1), ScDup(C12-1) and ScDup(C12-3)
8% (vol vol^−1^) ethanol	ScDup(C1-2), ScDup(C11-3), ScDup(C13-2), ScDup(C14-1), ScDup(C14-3) and ScDup(C16-3)	ScDup(C5-3), ScDup(C7-5), ScDup(C15-2), ScDup(C16-2) and ScDup(C16-4)
0.44% (wt vol^−1^) sulfuric acid (pH 2.3)	ScDup(C2-4), ScDup(C4-8), ScDup(C14-1), ScDup(C13-3) and ScDup(C16-3)	ScDup(C1-1), ScDup(C3-1), ScDup(C3-2)
80 mM acetic acid	ScDup(C7-1) and ScDup(C10-2)	ScDup(C2-3) and ScDup(C4-8)
36 mM formic acid	ScDup(C10-2), ScDup(C14-1) and ScDup(C14-3)	ScDup(C2-3)
1.2 M NaCl	ScDup(C7-1)	ScDup(2-3), ScDup(3-1), ScDup(7-5), ScDup(12-3), ScDup(13-1) and ScDup(16-4)
3% glycerol	ScDup(C2-2), ScDup(C10-2) and ScDup(C12-2)	–
pH 9	ScDup(C4-6), ScDup(C4-8) and ScDup(C6-2)	–
39 °C, 40 °C	ScDup(C1-2), ScDup(C4-3), ScDup(C4-8), ScDup(C5-1), ScDup(C5-2), ScDup(C7-1), ScDup(C9-1), ScDup(C9-2), ScDup(11-3), ScDup(C14-1) and ScDup(C16-5)	ScDup(C3-1), ScDup(C3-2), ScDup(C5-3), ScDup(C7-5) ScDup(C10-2), ScDup(C12-3), and ScDup(15-2)
13 °C	ScDup(C2-4), ScDup(C7-1), ScDup(C12-1), ScDup(C14-3) and ScDup(C14-4)	–

**Table 4:** Characteristics of duplication of sub-regions in unduplicated regions.

Region	Sub-region	Strain name	Chromosome location^a^	Plasmid template^b^	Duplication length (kb)	Number of genes	Transformants (n)	Proportion of desired karyotype^c^
C4-2	S1	ScDup(C4-2-S1)	Chr. IV 198,996-252,217	p3009, p3122	50	31	41	21% (3/14)
C4-2	S2	ScDup(C4-2-S2)	Chr. IV 250,614-301,020	p3009, p3122	50	33	50	14% (2/14)
C4-2	S3	ScDup(C4-2-S3)	Chr. IV 300,644-352,049	p3009, p3122	50	34	42	7% (1/14)
C4-2	S4	ScDup(C4-2-S4)	Chr. IV 350,404-401,638	p3122, p3279	50	30	1280	0% (0/52)
C4-2	S3+S4	ScDup(C4-2-(S3+S4))	Chr. IV 300,644-401,638	p3009, p3122	100	64	4	0% (0/4)
C4-4	S1	ScDup(C4-4-S1)	Chr. IV 599,793-652,548	p3009, p3122	50	34	58	2% (1/58)
C4-4	S2	ScDup(C4-4-S2)	Chr. IV 652,530-700,502	p3122, p3279	50	30	1067	0% (0/42)
C4-4	S3	ScDup(C4-4-S3)	Chr. IV 699,320-751,746	p3009, p3122	50	25	65	7% (1/14)
C4-4	S4	ScDup(C4-4-S4)	Chr. IV 750,633-795,723	p3009, p3122	50	25	22	18% (4/22)
C4-4	S2+S3	ScDup(C4-4-(S2+S3))	Chr. IV 652,530-751,746	p3009, p3122	100	55	17	0% (0/17)
C4-5	S1	ScDup(C4-5-S1)	Chr. IV 795,193-845,861	p3009, p3122	50	31	82	27% (4/15)
C4-5	S2	ScDup(C4-5-S2)	Chr. IV 844,952- 900,006	p3009, p3122	50	34	91	3% (1/30)
C4-5	S3	ScDup(C4-5-S3)	Chr. IV 898,551-951,323	p3009, p3122	50	33	56	13% (2/15)
C4-5	S4	ScDup(C4-5-S4)	Chr. IV 949,563-1,000,877	p3122, p3279	50	36	123	1% (1/104)
C4-7	S1	ScDup(C4-7-S1)	Chr. IV 1,198,183-1,250,760	p3009, p3122	50	38	15	8% (1/13)
C4-7	S2	ScDup(C4-7-S2)	Chr. IV 1,249,137-1,299,139	p3009, p3122	50	32	12	16% (2/12)
C4-7	S3	ScDup(C4-7-S3)	Chr. IV 1,297,392-1,350,890	p3009, p3122	50	31	39	14% (2/14)
C4-7	S4	ScDup(C4-7-S4)	Chr. IV 1,349,318-1,402,247	p3122, p3279	50	33	822	0% (0/42)
C4-7	S3+S4	ScDup(C4-7-(S3+S4))	Chr. IV 1,297,392-1,402,247	p3009, p3122	100	64	27	0% (0/27)
C6-1	S1	ScDup(C6-1-S1)	Chr. VI 1-48,730	p3122	50	30	8	63% (5/8)
C6-1	S2	ScDup(C6-1-S2)	Chr. VI 47,761-98,498	p3009, p3122	50	27	24	0% (0/24)
C7-4	S1	ScDup(C7-4-S1)	Chr. VII 598,443-651,547	p3122, p3279	50	34	901	2% (1/56)
C7-4	S2	ScDup(C7-4-S2)	Chr. VII 650,314-701,698	p3009, p3122	50	25	39	7% (1/14)
C7-4	S3	ScDup(C7-4-S3)	Chr. VII 701,628-754,816	p3009, p3122	50	39	15	7% (1/15)
C7-4	S4	ScDup(C7-4-S4)	Chr. VII 753,704-801,057	p3009, p3122	50	35	65	21% (3/14)
C7-4	S4	ScDup(C7-4-S4_2)	Chr. VII 753,704-801,057	p3122, p3279	50	35	200	4% (1/28)
C8-2	S1	ScDup(C8-2-S1)	Chr. VIII 203,559-250,652	p3009, p3122	50	46	84	7% (1/14)
C8-2	S2	ScDup(C8-2-S2)	Chr. VIII 250,081-302,950	p3009, p3122	50	27	82	7% (1/14)
C8-2	S3	ScDup(C8-2-S3)	Chr. VIII 301,788-350,205	p3122, p3279	50	21	1500	0% (0/41)
C8-2	S4	ScDup(C8-2-S4)	Chr. VIII 348,556-401,907	p3009, p3122	50	46	109	7% (1/14)
C8-2	S3+S4	ScDup(C8-2-(S3+S4))	Chr. VIII 301,788-401,907	p3009, p3122	100	67	23	0% (0/23)
C11-2	S1	ScDup(C11-2-S1)	Chr. XI 199,892-246,288	p3009, p3122	50	31	7	14% (1/7)
C11-2	S2	ScDup(C11-2-S2)	Chr. XI 245,144-300,075	p3122, p3279	50	35	961	0% (0/28)
C11-2	S3	ScDup(C11-2-S3)	Chr. XI 298,583-350,129	p3009, p3122	50	35	36	21% (3/14)
C11-2	S4	ScDup(C11-2-S4)	Chr. XI 348,413-399,750	p3009, p3122	50	32	3	33% (1/3)
C11-2	S1+S2	ScDup(C11-2-(S1+S2))	Chr. XI 199,892-300,075	p3009, p3122	100	66	81	0% (0/28)
C14-2	S1	ScDup(C14-2-S1)	Chr. XIV 199,575-251,006	p3009, p3122	50	31	2	100% (2/2)
C14-2	S2	ScDup(C14-2-S2)	Chr. XIV 250,863-302,108	p3009, p3122	50	33	8	13% (1/8)
C14-2	S3	ScDup(C14-2-S3)	Chr. XIV 301,698-349,197	p3009, p3122	50	31	19	5% (1/19)
C14-2	S4	ScDup(C14-2-S4)	Chr. XIV 349,012-403,514	p3122, p3279	50	37	154	0% (0/75)
C14-2	S3+S4	ScDup(C14-2-(S3+S4))	Chr. XIV 301,698-403,514	p3009, p3122	100	68	17	0% (0/17)

^a^Chr. N x-y: Chr. N represents chromosome number, x represents first nucleotide number of chromosomal region and y represents last nucleotide number of chromosomal region.

^b^p3009 was used to amplify the *CgHIS3* cassette, p3122 was used to amplify the *CEN4-CgLEU2* cassette, p3279 was used to amplify the *CgHIS3-H4ARS* cassette

^c^Proportion of desired karyotype in analyzed transformants (number of segmental aneuploids/number of candidate transformants that were analyzed for karyotype).
