# Supplementary material for: Genome-wide construction of a series of designed segmental aneuploids in Saccharomyces cerevisiae
Source: Sci Rep. 2015 Jul 30;5:12510. doi: 10.1038/srep12510 (PMC4519793; doi:10.1038/srep12510)
Supplement: Supplementary Information [file srep12510-s1.pdf]

Supplementary Information for

Genome-wide construction of a series of designed segmental aneuploids in *Saccharomyces cerevisiae*

Waranya Natesuntorn, Kotaro Iwami, Yuki Matsubara, Yu Sasano, Minetaka Sugiyama, Yoshinobu Kaneko and Satoshi Harashima

<u>Table of contents</u>

Supplementary Figure 1 Karyotypic analysis of segmental aneuploids for chromosomes I to VI…..………………..……………………………………………………….…..…3

Supplementary Figure 2 Growth profiles of segmental aneuploid strains in SC medium at 30°C for 24 hours……..…………………..…………………………........…………7

Supplementary Figure 3 Phenotypic assays of segmental aneuploid strains for chromosomes I to XVI …………………………….……………..………….......…8

Supplementary Figure 4 Analysis of the relationship between segmental duplication and phenotype using a chromosome loss strategy …….……………………...……….....17

Supplementary Figure 5 PFGE analysis of segmental aneuploid strains and derivative strains that had lost the duplicated chromosome ….………………………......…21

Supplementary Table 1 Growth of segmental aneuploids for chromosomes I to XVI under various stresses…...……….…………………….…………………….…..........22

Supplementary Table 2 Stress sensitive and resistant phenotypes of segmental aneuploids for chromosomes I to XVI ……………………..……….........……..…25

Supplementary Table 3 Genes located in 50 kb unduplicated sub-regions whose overexpression is associated with cell lethality or abnormalities in cell cycle progression or the actin skeleton…..…………………….……….……………………..…27

Supplementary Table 4 Genes whose upper copy number limit is less than 3 …..…....29

Supplementary Table 5 Genes located in duplicated chromosome regions whose overexpression cause sensitive or resistant phenotypes ……….………..…........…..30

Supplementary Table 6 Plasmids used in this study…………………………….……...31

Supplementary Table 7 Primers used for construction of segmental chromosome duplications of chromosomes I to XVI ……………………………………...…...32

Supplementary Table 8 Primers used for construction of segmental chromosome duplications of sub-regions of unduplicated regions ……………………..………..…..37

Supplementary Table 9 Primers used for estimation of the maximum length of segmental chromosome duplication………………………………….………..…….41

Supplementary Table 10 Primers used to amplify probes for detection of segmental chromosome duplications of chromosomes I to XVI ……..…………………….…….43

Supplementary Table 11 Primers used to amplify probes for detection of segmental chromosome duplication of sub-regions……………………..………………..….46

Supplementary Table 12 Primers used to amplify probes for estimation of the maximum length of segmental chromosome duplication……………………………….......48

References……………………………………………………………….……....49

Supplementary Figure 1. Karyotypic analysis of segmental aneuploids for chromosomes I to XVI. PFGE analysis was performed followed by Southern blot analysis using a probe consisting of nucleotide sequences that corresponded to the target region.

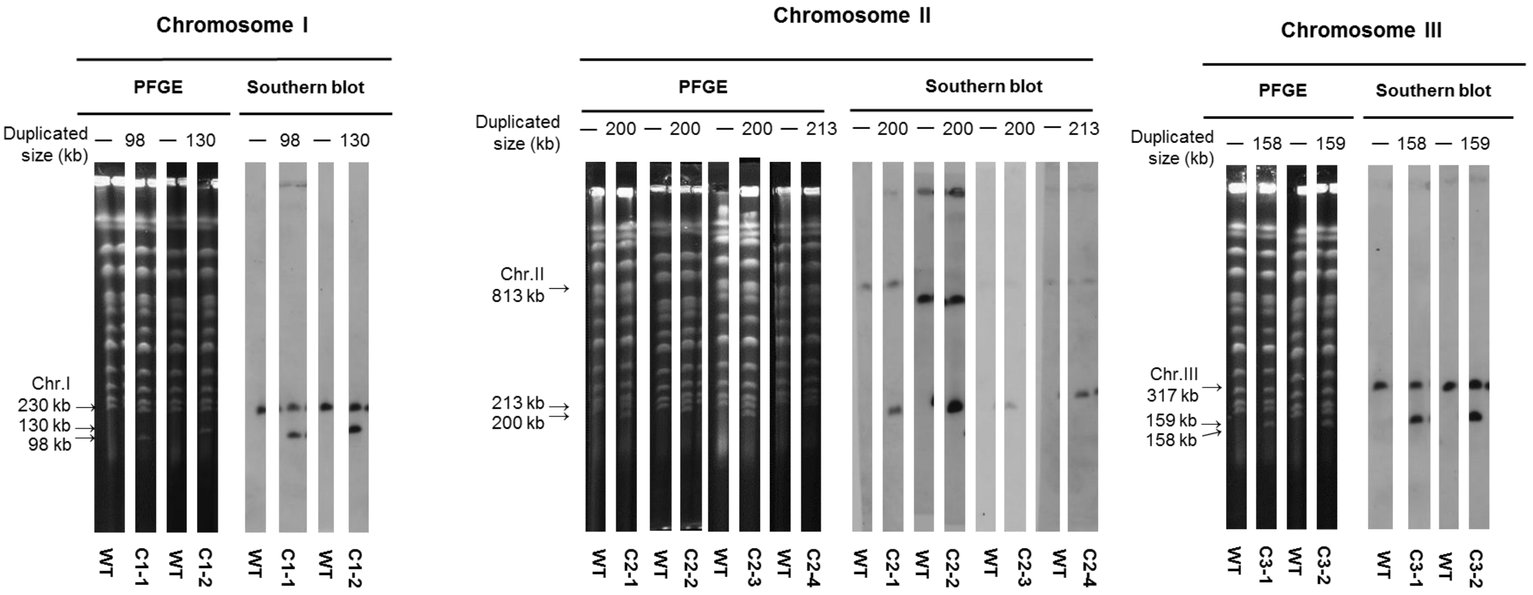

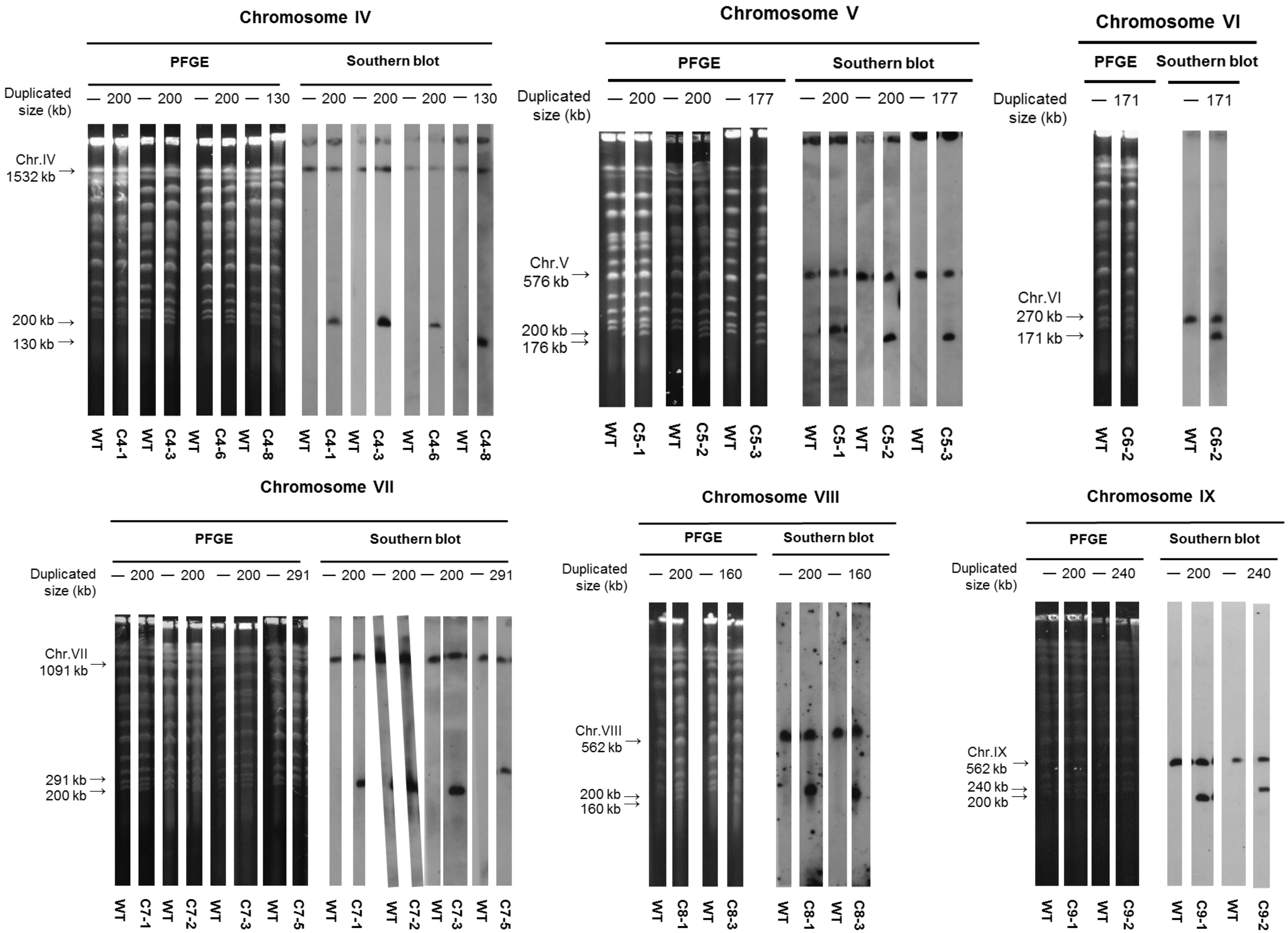
Chromosome IV
PFGE
Southern blot
Duplicated size (kb)
— 200 — 200 — 200 — 130 — 200 — 200 — 200 — 130
Chr.IV 1532 kb
200 kb
130 kb
WT C4-1 WT C4-3 WT C4-6 WT C4-8 WT C4-1 WT C4-3 WT C4-6 WT C4-8
Chromosome V
PFGE
Southern blot
Duplicated size (kb)
— 200 — 200 — 177 — 200 — 200 — 177
Chr.V 576 kb
200 kb
176 kb
WT C5-1 WT C5-2 WT C5-3 WT C5-1 WT C5-2 WT C5-3
Chromosome VI
PFGE
Southern blot
Duplicated size (kb)
— 171 — 171
Chr.VI 270 kb
171 kb
WT C6-2 WT C6-2
Chromosome VII
PFGE
Southern blot
Duplicated size (kb)
— 200 — 200 — 200 — 291 — 200 — 200 — 200 — 291
Chr.VII 1091 kb
291 kb
200 kb
WT C7-1 WT C7-2 WT C7-3 WT C7-5 WT C7-1 WT C7-2 WT C7-3 WT C7-5
Chromosome VIII
PFGE
Southern blot
Duplicated size (kb)
— 200 — 160 — 200 — 160
Chr.VIII 562 kb
200 kb
160 kb
WT C8-1 WT C8-3 WT C8-1 WT C8-3
Chromosome IX
PFGE
Southern blot
Duplicated size (kb)
— 200 — 240 — 200 — 240
Chr.IX 562 kb
240 kb
200 kb
WT C9-1 WT C9-2 WT C9-1 WT C9-2

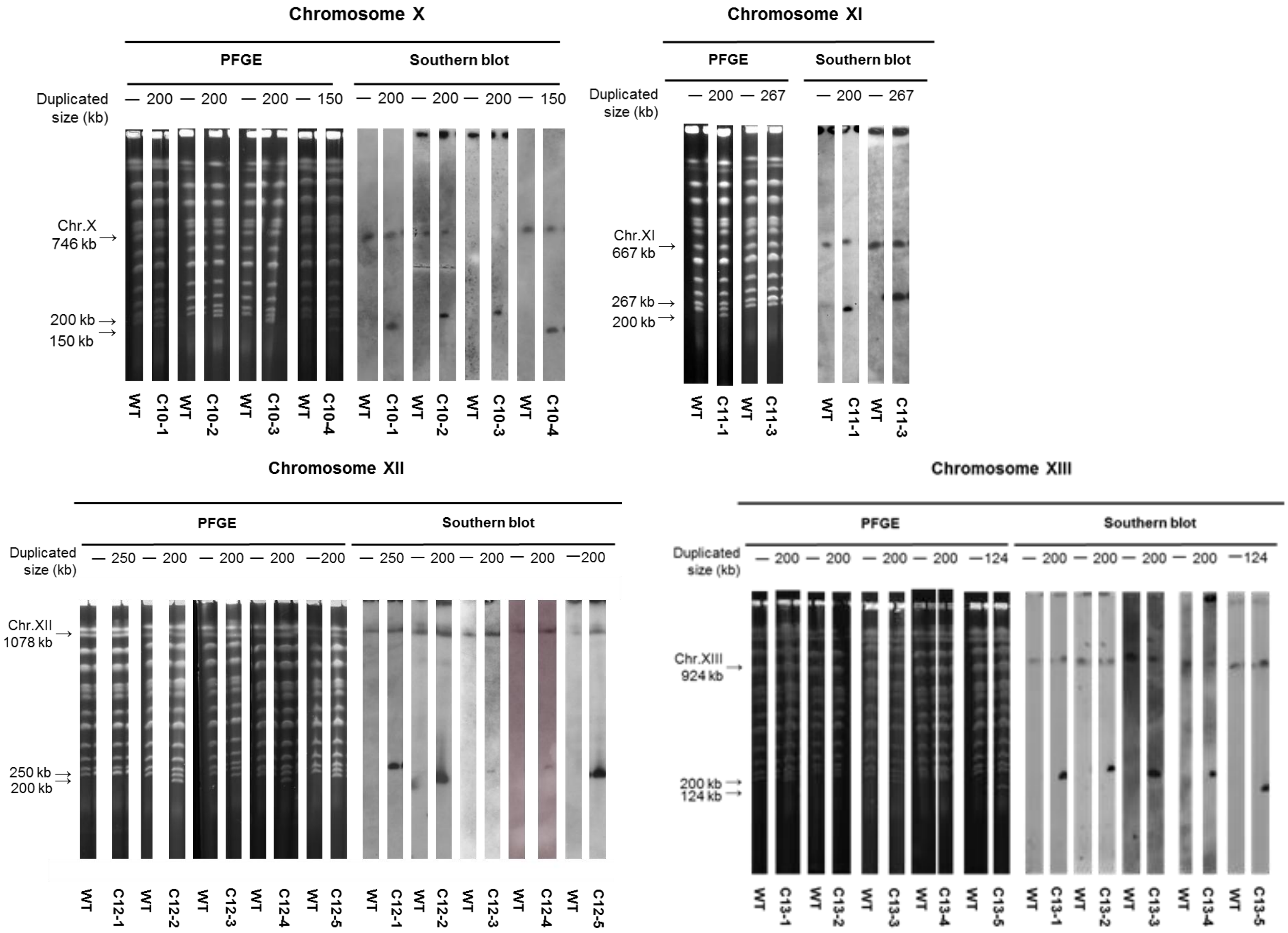
Chromosome X
PFGE
Southern blot
Duplicated size (kb)
— 200 — 200 — 200 — 150
— 200 — 200 — 200 — 150
Chr.X 746 kb
200 kb
150 kb
WT C10-1 WT C10-2 WT C10-3 WT C10-4
WT C10-1 WT C10-2 WT C10-3 WT C10-4
Chromosome XI
PFGE
Southern blot
Duplicated size (kb)
— 200 — 267
— 200 — 267
Chr.XI 667 kb
267 kb
200 kb
WT C11-1 WT C11-3
WT C11-1 WT C11-3
Chromosome XII
PFGE
Southern blot
Duplicated size (kb)
— 250 — 200 — 200 — 200 —200
— 250 — 200 — 200 — 200 —200
Chr.XII 1078 kb
250 kb
200 kb
WT C12-1 WT C12-2 WT C12-3 WT C12-4 WT C12-5
WT C12-1 WT C12-2 WT C12-3 WT C12-4 WT C12-5
Chromosome XIII
PFGE
Southern blot
Duplicated size (kb)
— 200 — 200 — 200 — 200 —124
— 200 — 200 — 200 — 200 —124
Chr.XIII 924 kb
200 kb
124 kb
WT C13-1 WT C13-2 WT C13-3 WT C13-4 WT C13-5
WT C13-1 WT C13-2 WT C13-3 WT C13-4 WT C13-5

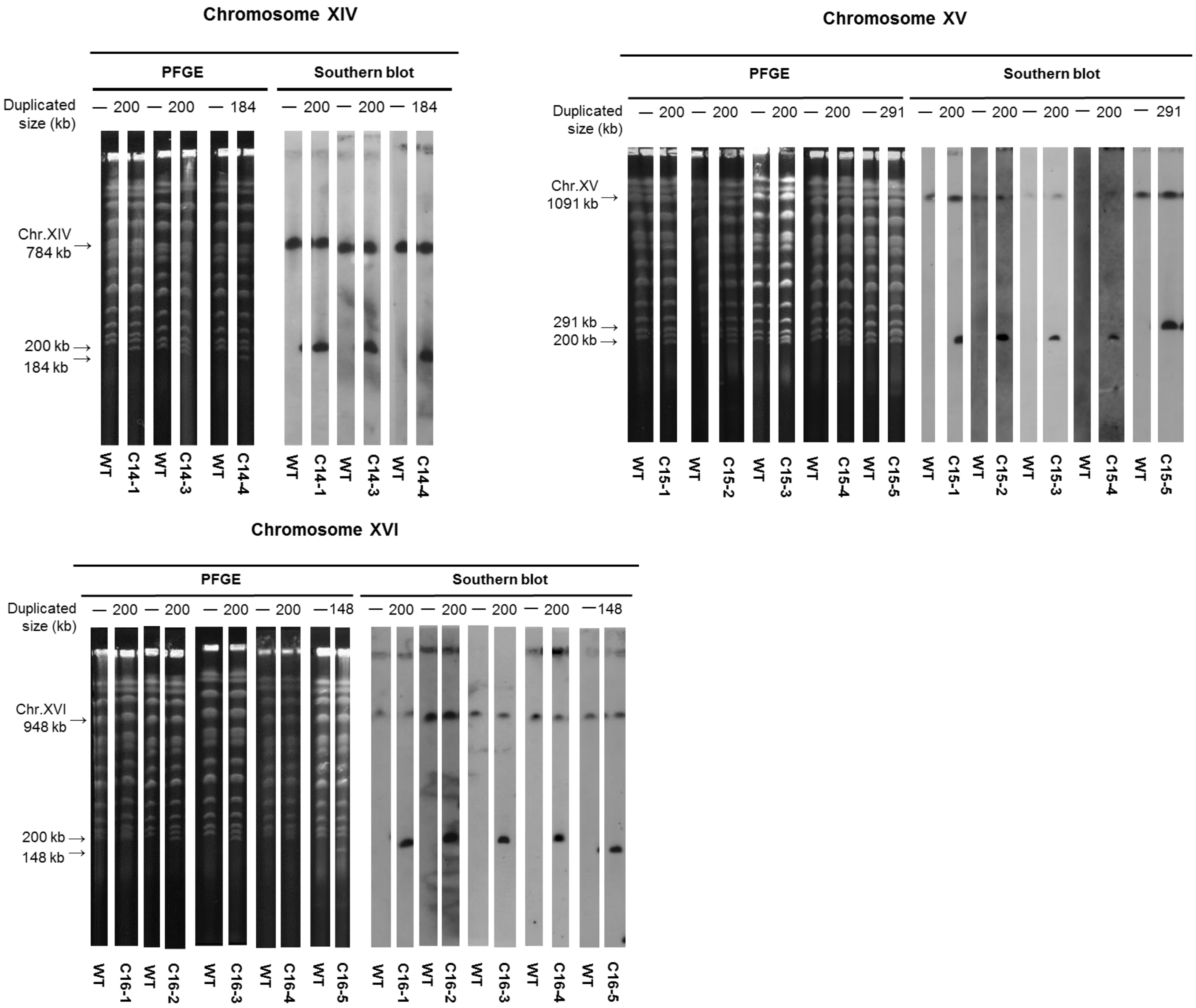
Chromosome XIV
PFGE
Southern blot
Duplicated size (kb)
200
200
184
200
200
184
Chr.XIV 784 kb
200 kb
184 kb
WT
C14-1
WT
C14-3
WT
C14-4
Chromosome XV
PFGE
Southern blot
Duplicated size (kb)
200
200
200
200
291
Chr.XV 1091 kb
291 kb
200 kb
WT
C15-1
WT
C15-2
WT
C15-3
WT
C15-4
WT
C15-5
Chromosome XVI
PFGE
Southern blot
Duplicated size (kb)
200
200
200
200
148
Chr.XVI 948 kb
200 kb
148 kb
WT
C16-1
WT
C16-2
WT
C16-3
WT
C16-4
WT
C16-5

Supplementary Figure 2. Growth profiles of segmental aneuploid strains in SC medium at 30°C for 24 hours. The OD_{660} of 53 segmental aneuploid strains and the parental strain was measured every 2 hours. Three independent replicate cultures were performed.

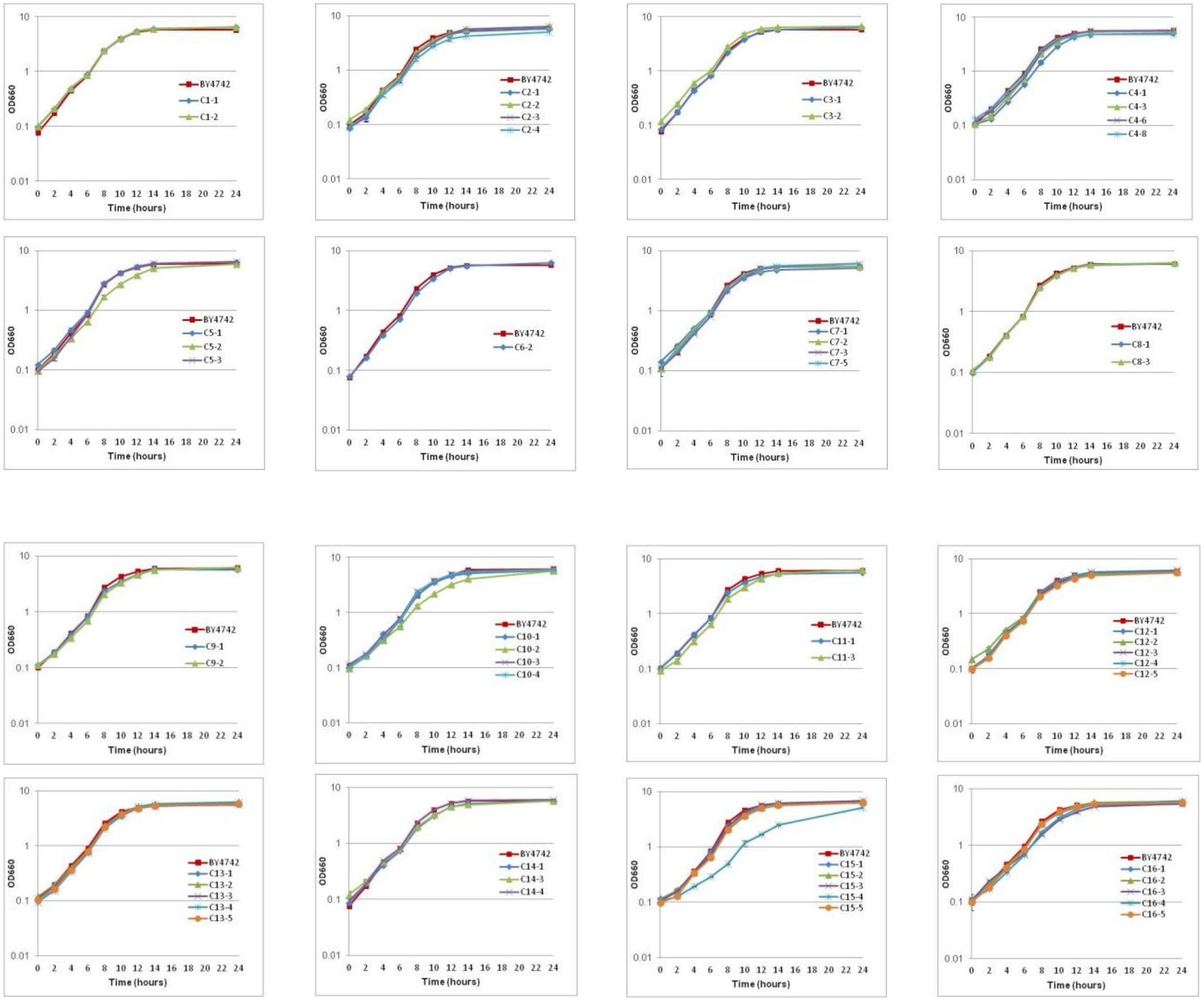

Supplementary Figure 3. **Phenotypic assays of segmental aneuploid strains for chromosomes I to XVI.**

Ten-fold serial dilutions of segmental aneuploid strains of chromosomes I to XVI (a-p, respectively) were spotted on plates and subjected to different stresses including 4% (wt vol^{-1}) lactic acid, 5% (wt vol^{-1}) lactic acid, 6% (wt vol^{-1}) lactic acid, 6% (vol vol^{-1}) ethanol, 8% (vol vol^{-1}) ethanol, 10% (vol vol^{-1}) ethanol, 0.41% (wt vol^{-1}) sulfuric acid (pH 2.4), 0.44% (wt vol^{-1}) sulfuric acid (pH 2.3), 0.47% (wt vol^{-1}) sulfuric acid (pH 2.2), 36 mM formic acid, 1.2 M NaCl, 80 mM acetic acid, YPEG, pH 9, at 13°C, at 39°C, at 40°C, at 41°C. The plates were incubated for 3-4 days before being photographed. Red arrow represents stress resistant phenotype. Blue arrow represents stress sensitive phenotype.

a)

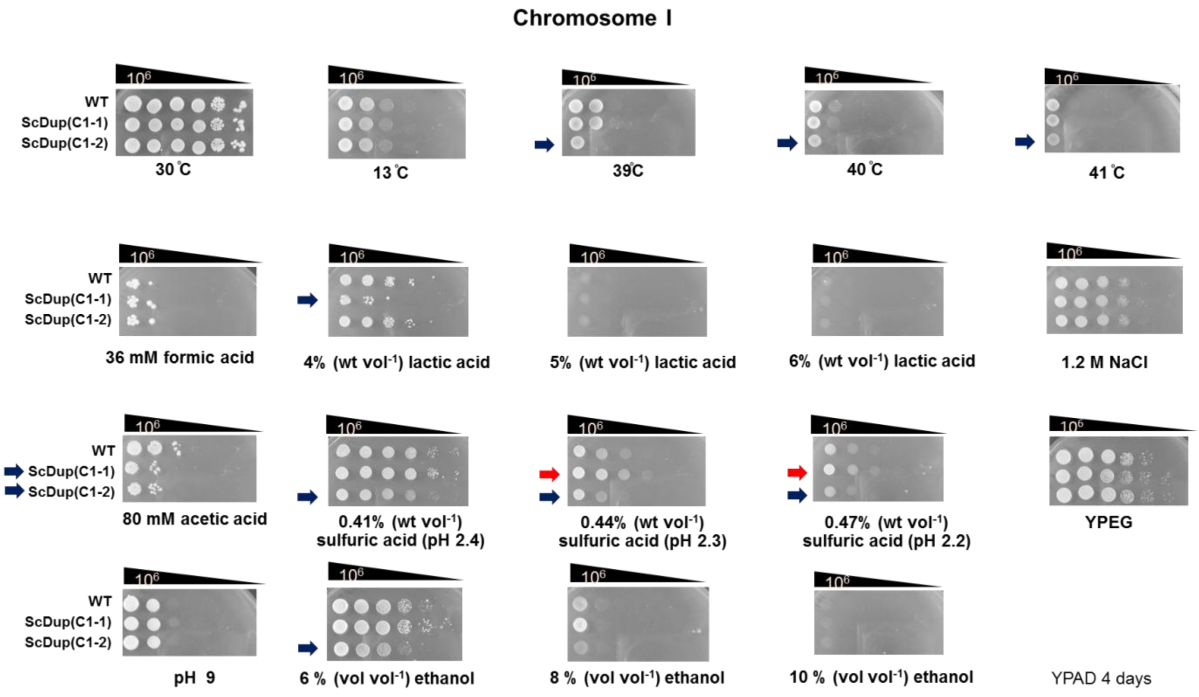

b)

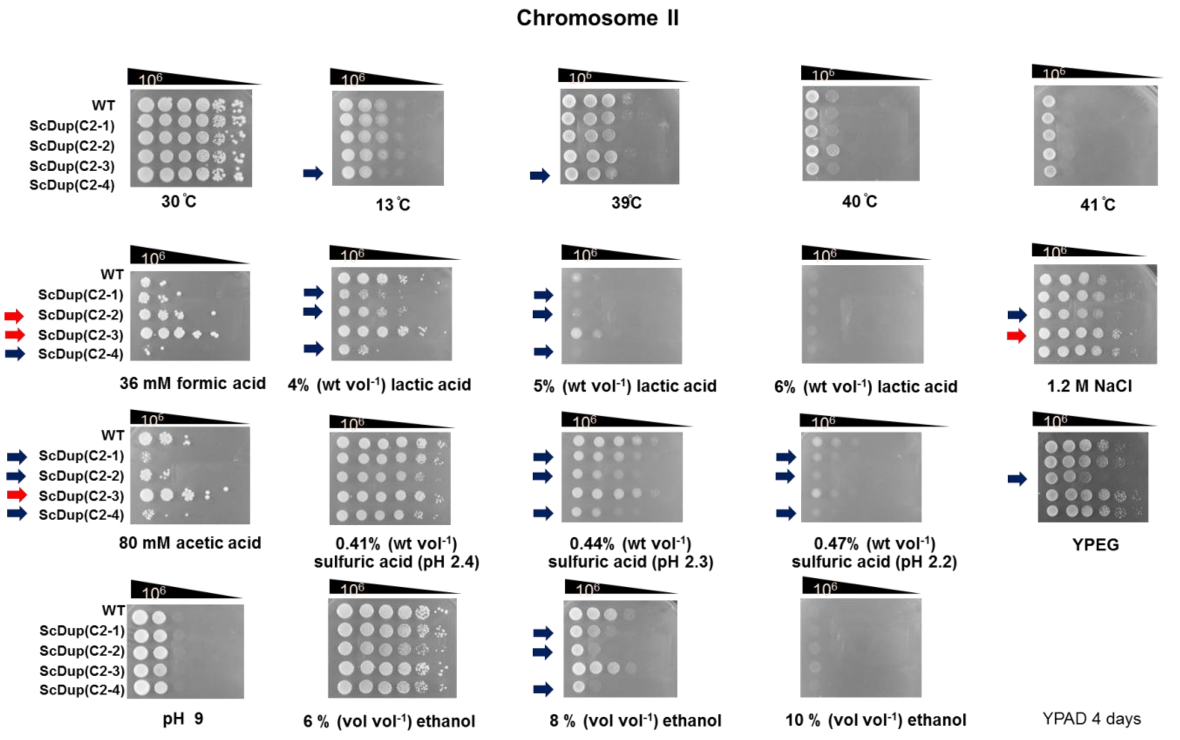

Chromosome II
WT
ScDup(C2-1)
ScDup(C2-2)
ScDup(C2-3)
ScDup(C2-4)
10^6
30 ℃
13 ℃
39℃
40 ℃
41 ℃
36 mM formic acid
4% (wt vol-1) lactic acid
5% (wt vol-1) lactic acid
6% (wt vol-1) lactic acid
1.2 M NaCl
80 mM acetic acid
0.41% (wt vol-1) sulfuric acid (pH 2.4)
0.44% (wt vol-1) sulfuric acid (pH 2.3)
0.47% (wt vol-1) sulfuric acid (pH 2.2)
YPEG
pH 9
6 % (vol vol-1) ethanol
8 % (vol vol-1) ethanol
10 % (vol vol-1) ethanol
YPAD 4 days

c)

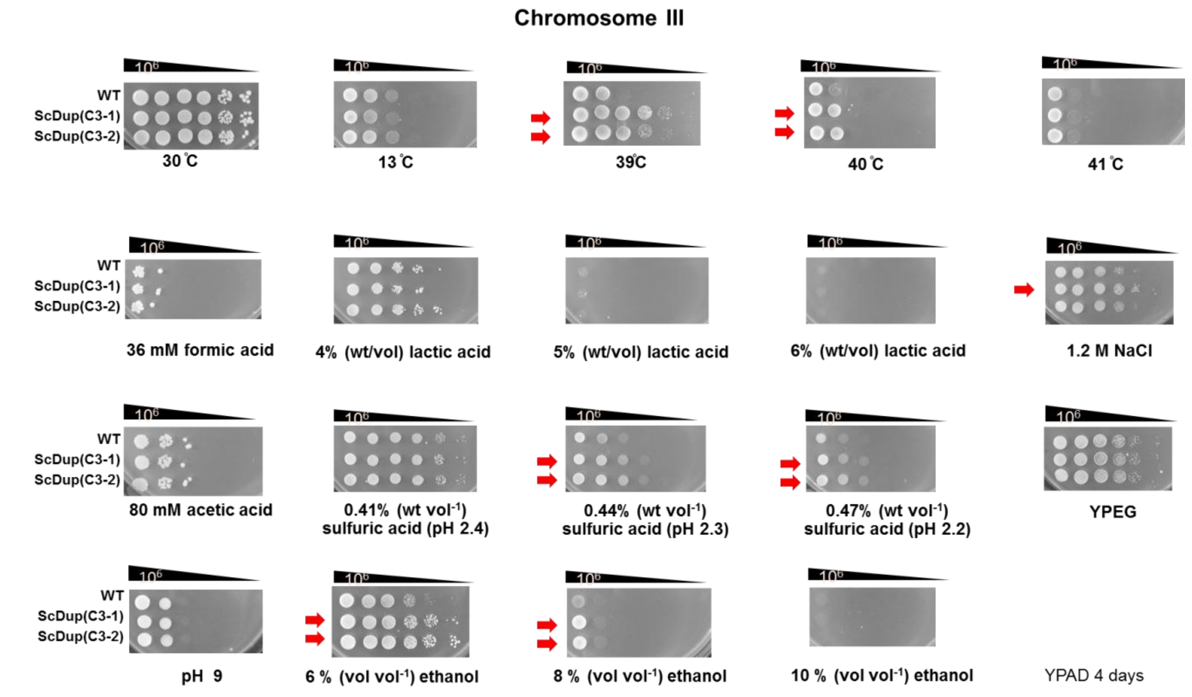

Chromosome III
WT
ScDup(C3-1)
ScDup(C3-2)
10^6
30 ℃
13 ℃
39℃
40 ℃
41 ℃
36 mM formic acid
4% (wt/vol) lactic acid
5% (wt/vol) lactic acid
6% (wt/vol) lactic acid
1.2 M NaCl
80 mM acetic acid
0.41% (wt vol-1) sulfuric acid (pH 2.4)
0.44% (wt vol-1) sulfuric acid (pH 2.3)
0.47% (wt vol-1) sulfuric acid (pH 2.2)
YPEG
pH 9
6 % (vol vol-1) ethanol
8 % (vol vol-1) ethanol
10 % (vol vol-1) ethanol
YPAD 4 days

d)

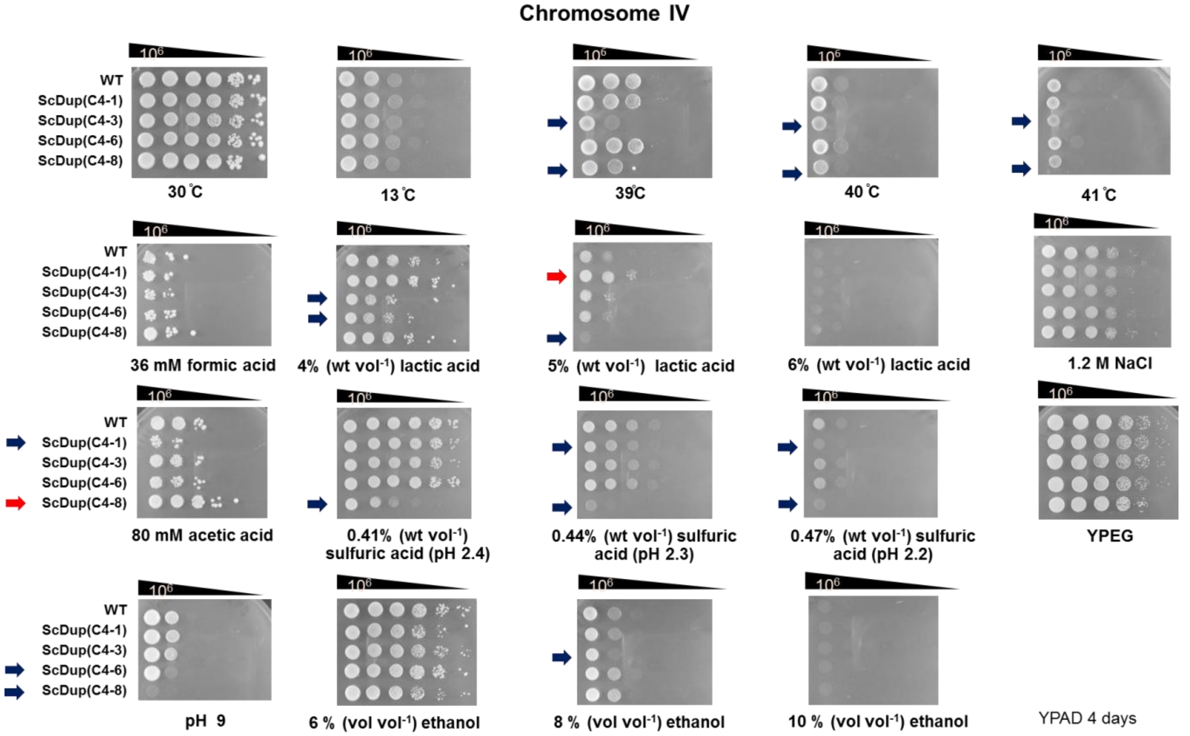
Chromosome IV
10^6
WT
ScDup(C4-1)
ScDup(C4-3)
ScDup(C4-6)
ScDup(C4-8)
30 ℃
13 ℃
39℃
40 ℃
41 ℃
36 mM formic acid
4% (wt vol-1) lactic acid
5% (wt vol-1) lactic acid
6% (wt vol-1) lactic acid
1.2 M NaCl
80 mM acetic acid
0.41% (wt vol-1) sulfuric acid (pH 2.4)
0.44% (wt vol-1) sulfuric acid (pH 2.3)
0.47% (wt vol-1) sulfuric acid (pH 2.2)
YPEG
pH 9
6 % (vol vol-1) ethanol
8 % (vol vol-1) ethanol
10 % (vol vol-1) ethanol
YPAD 4 days

e)

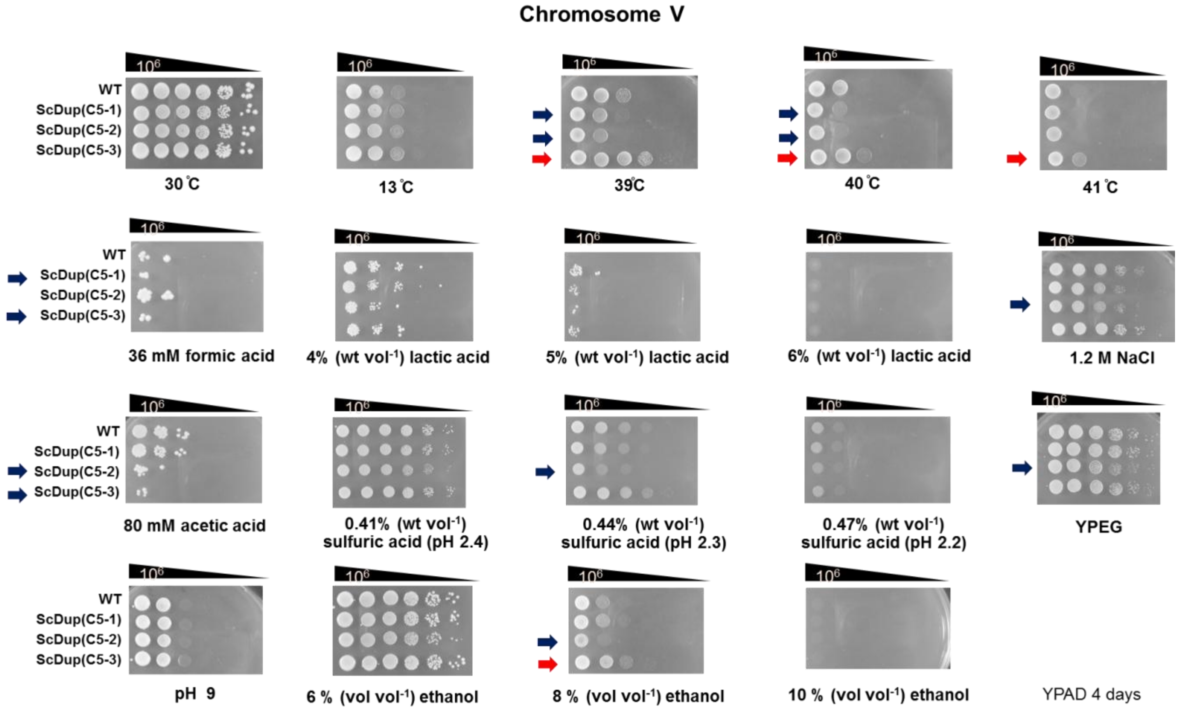
Chromosome V
10^6
WT
ScDup(C5-1)
ScDup(C5-2)
ScDup(C5-3)
30 ℃
13 ℃
39℃
40 ℃
41 ℃
36 mM formic acid
4% (wt vol-1) lactic acid
5% (wt vol-1) lactic acid
6% (wt vol-1) lactic acid
1.2 M NaCl
80 mM acetic acid
0.41% (wt vol-1) sulfuric acid (pH 2.4)
0.44% (wt vol-1) sulfuric acid (pH 2.3)
0.47% (wt vol-1) sulfuric acid (pH 2.2)
YPEG
pH 9
6 % (vol vol-1) ethanol
8 % (vol vol-1) ethanol
10 % (vol vol-1) ethanol
YPAD 4 days

f)

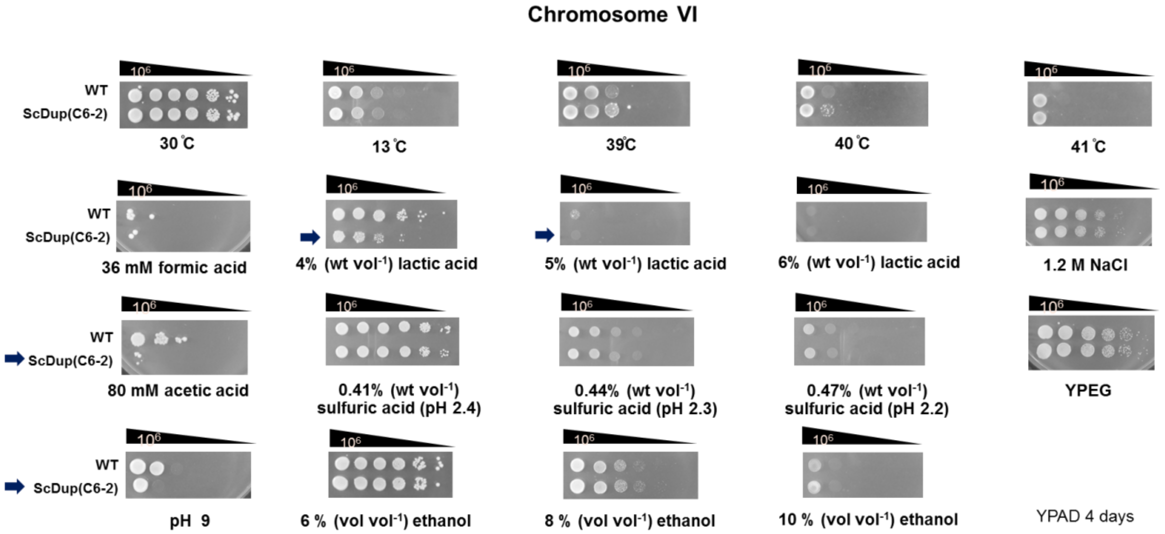
Chromosome VI
WT
ScDup(C6-2)
10^6
30 ℃
13 ℃
39℃
40 ℃
41 ℃
36 mM formic acid
4% (wt vol-1) lactic acid
5% (wt vol-1) lactic acid
6% (wt vol-1) lactic acid
1.2 M NaCl
80 mM acetic acid
0.41% (wt vol-1) sulfuric acid (pH 2.4)
0.44% (wt vol-1) sulfuric acid (pH 2.3)
0.47% (wt vol-1) sulfuric acid (pH 2.2)
YPEG
pH 9
6 % (vol vol-1) ethanol
8 % (vol vol-1) ethanol
10 % (vol vol-1) ethanol
YPAD 4 days

g)

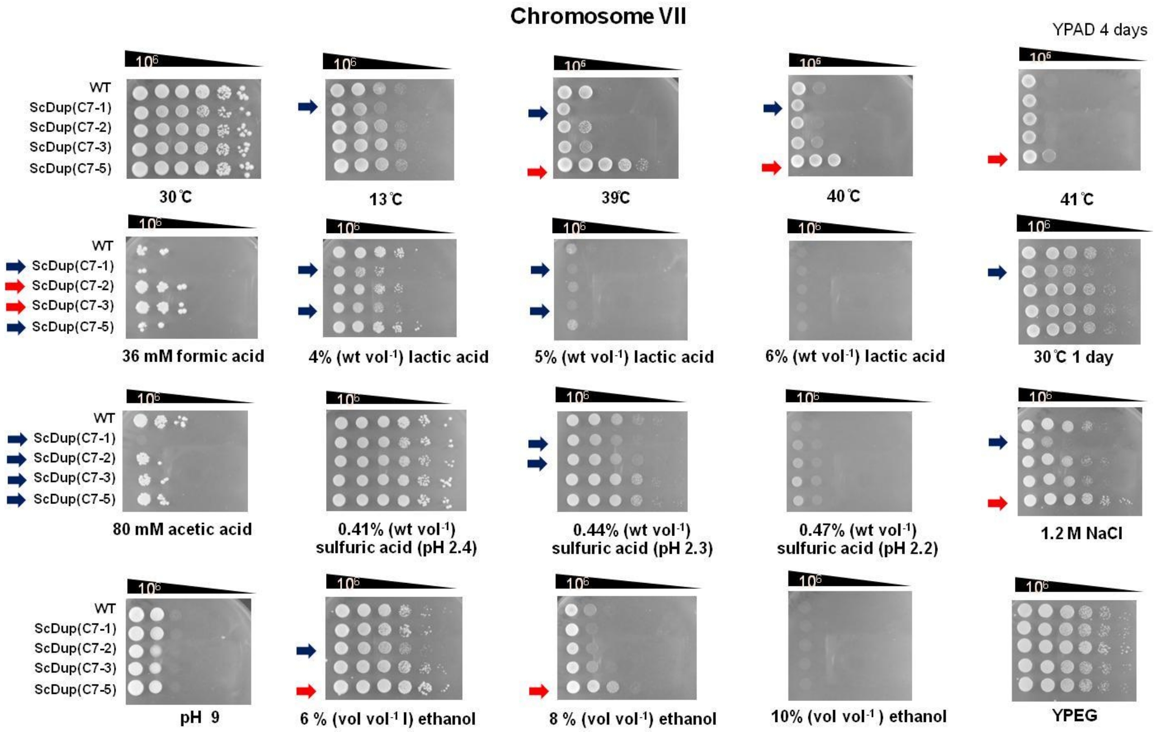
Chromosome VII
YPAD 4 days
WT
ScDup(C7-1)
ScDup(C7-2)
ScDup(C7-3)
ScDup(C7-5)
10^6
30 ℃
13 ℃
39℃
40 ℃
41 ℃
36 mM formic acid
4% (wt vol-1) lactic acid
5% (wt vol-1) lactic acid
6% (wt vol-1) lactic acid
30 ℃ 1 day
80 mM acetic acid
0.41% (wt vol-1) sulfuric acid (pH 2.4)
0.44% (wt vol-1) sulfuric acid (pH 2.3)
0.47% (wt vol-1) sulfuric acid (pH 2.2)
1.2 M NaCl
pH 9
6 % (vol vol-1 l) ethanol
8 % (vol vol-1) ethanol
10% (vol vol-1) ethanol
YPEG

h)

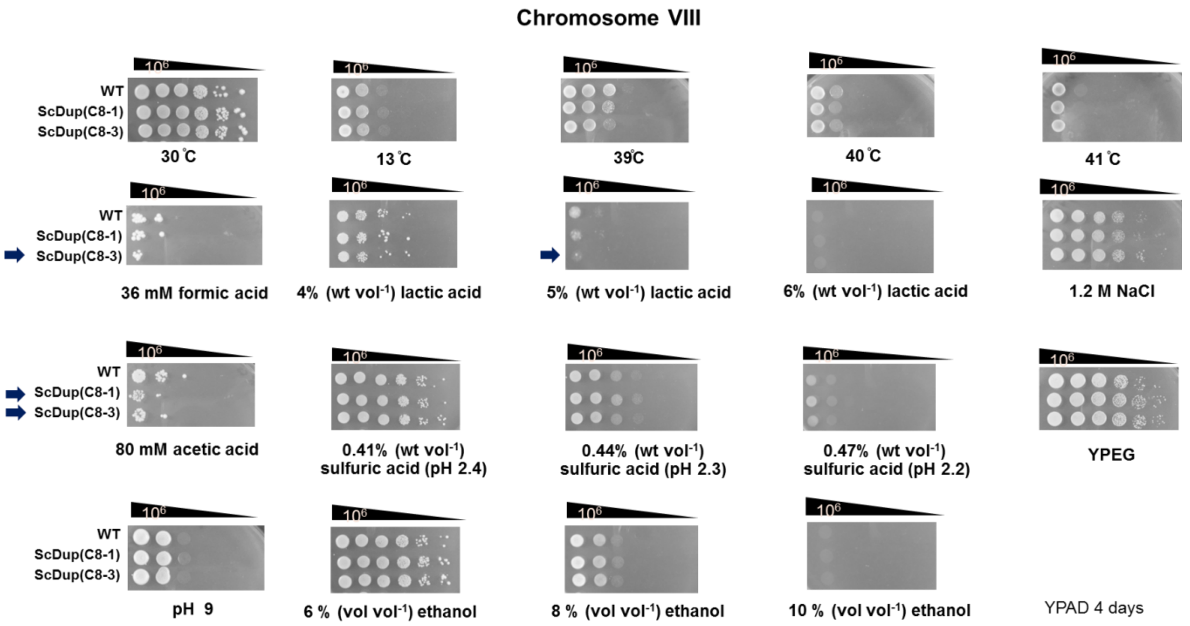
Chromosome VIII
WT
ScDup(C8-1)
ScDup(C8-3)
30 ℃
13 ℃
39℃
40 ℃
41 ℃
36 mM formic acid
4% (wt vol-1) lactic acid
5% (wt vol-1) lactic acid
6% (wt vol-1) lactic acid
1.2 M NaCl
80 mM acetic acid
0.41% (wt vol-1) sulfuric acid (pH 2.4)
0.44% (wt vol-1) sulfuric acid (pH 2.3)
0.47% (wt vol-1) sulfuric acid (pH 2.2)
YPEG
pH 9
6 % (vol vol-1) ethanol
8 % (vol vol-1) ethanol
10 % (vol vol-1) ethanol
YPAD 4 days

i)

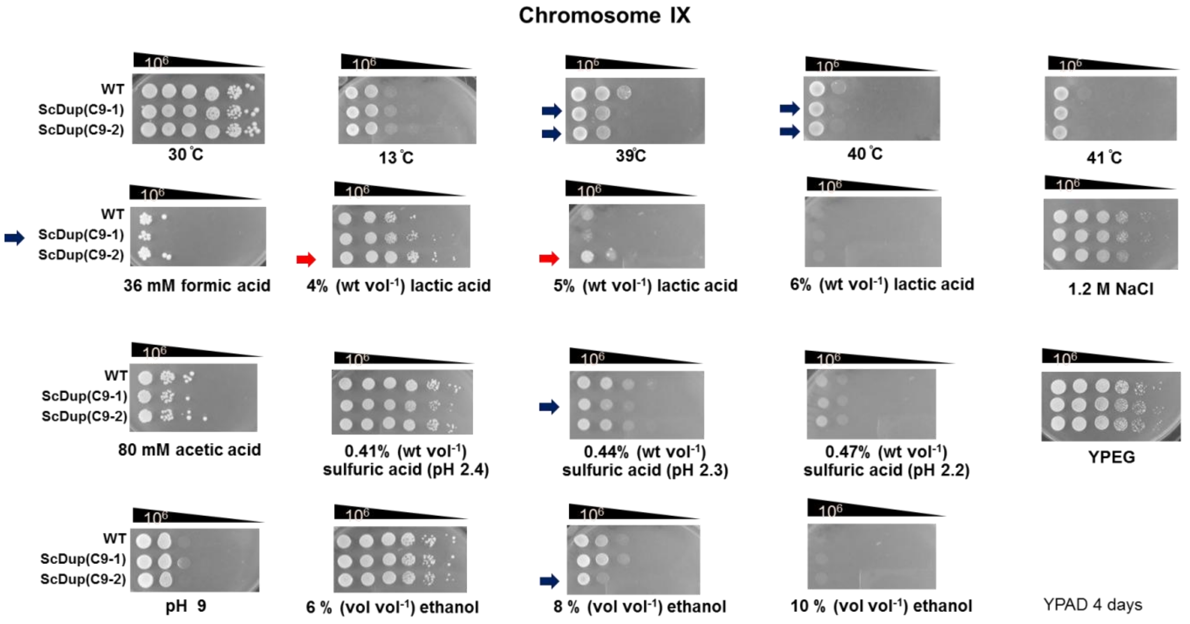
Chromosome IX
WT
ScDup(C9-1)
ScDup(C9-2)
30 ℃
13 ℃
39℃
40 ℃
41 ℃
36 mM formic acid
4% (wt vol-1) lactic acid
5% (wt vol-1) lactic acid
6% (wt vol-1) lactic acid
1.2 M NaCl
80 mM acetic acid
0.41% (wt vol-1) sulfuric acid (pH 2.4)
0.44% (wt vol-1) sulfuric acid (pH 2.3)
0.47% (wt vol-1) sulfuric acid (pH 2.2)
YPEG
pH 9
6 % (vol vol-1) ethanol
8 % (vol vol-1) ethanol
10 % (vol vol-1) ethanol
YPAD 4 days

j)

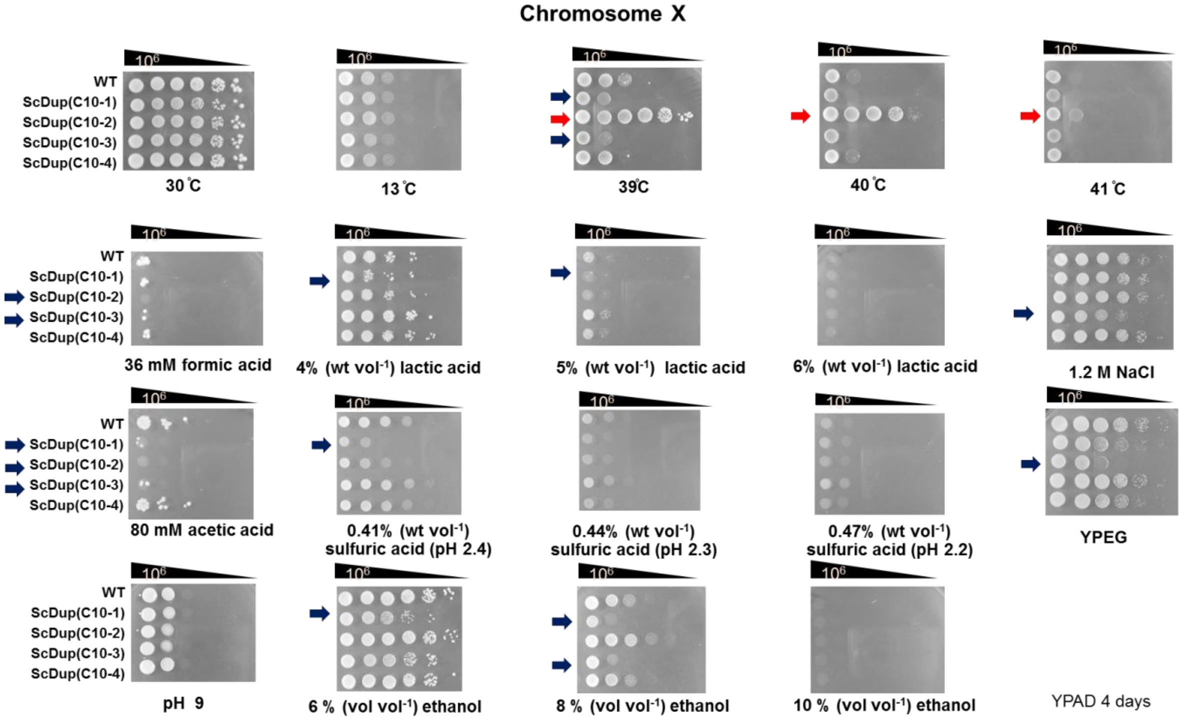
Chromosome X
WT
ScDup(C10-1)
ScDup(C10-2)
ScDup(C10-3)
ScDup(C10-4)
30 ℃
13 ℃
39℃
40 ℃
41 ℃
36 mM formic acid
4% (wt vol-1) lactic acid
5% (wt vol-1) lactic acid
6% (wt vol-1) lactic acid
1.2 M NaCl
80 mM acetic acid
0.41% (wt vol-1) sulfuric acid (pH 2.4)
0.44% (wt vol-1) sulfuric acid (pH 2.3)
0.47% (wt vol-1) sulfuric acid (pH 2.2)
YPEG
pH 9
6 % (vol vol-1) ethanol
8 % (vol vol-1) ethanol
10 % (vol vol-1) ethanol
YPAD 4 days

k)

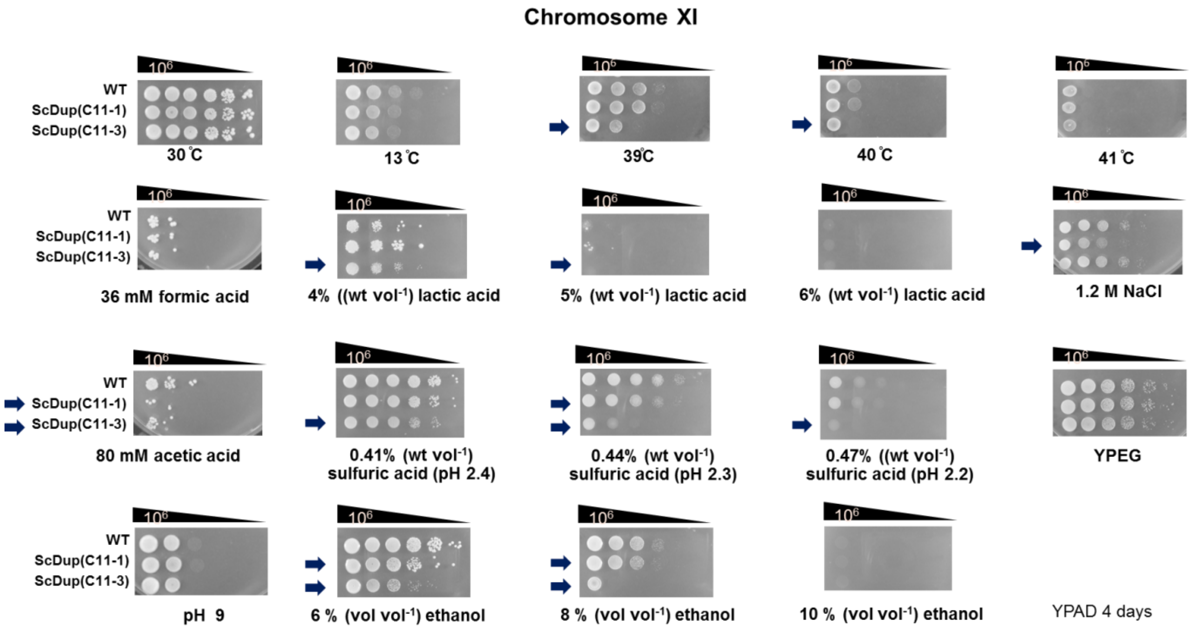
Chromosome XI
WT
ScDup(C11-1)
ScDup(C11-3)
30 ℃
13 ℃
39℃
40 ℃
41 ℃
36 mM formic acid
4% ((wt vol-1) lactic acid
5% (wt vol-1) lactic acid
6% (wt vol-1) lactic acid
1.2 M NaCl
80 mM acetic acid
0.41% (wt vol-1) sulfuric acid (pH 2.4)
0.44% (wt vol-1) sulfuric acid (pH 2.3)
0.47% ((wt vol-1) sulfuric acid (pH 2.2)
YPEG
pH 9
6 % (vol vol-1) ethanol
8 % (vol vol-1) ethanol
10 % (vol vol-1) ethanol
YPAD 4 days

l)

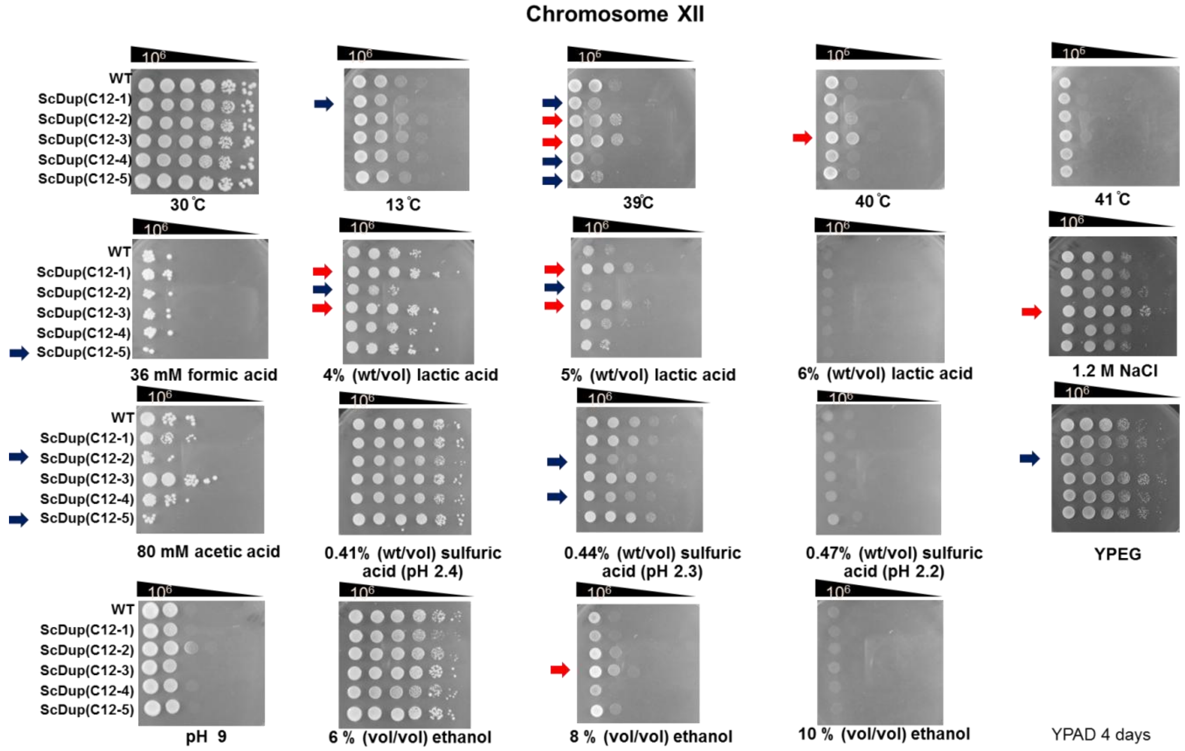
Chromosome XII
10^6
WT
ScDup(C12-1)
ScDup(C12-2)
ScDup(C12-3)
ScDup(C12-4)
ScDup(C12-5)
30 ℃
13 ℃
39℃
40 ℃
41 ℃
36 mM formic acid
4% (wt/vol) lactic acid
5% (wt/vol) lactic acid
6% (wt/vol) lactic acid
1.2 M NaCl
80 mM acetic acid
0.41% (wt/vol) sulfuric acid (pH 2.4)
0.44% (wt/vol) sulfuric acid (pH 2.3)
0.47% (wt/vol) sulfuric acid (pH 2.2)
YPEG
pH 9
6 % (vol/vol) ethanol
8 % (vol/vol) ethanol
10 % (vol/vol) ethanol
YPAD 4 days

m)

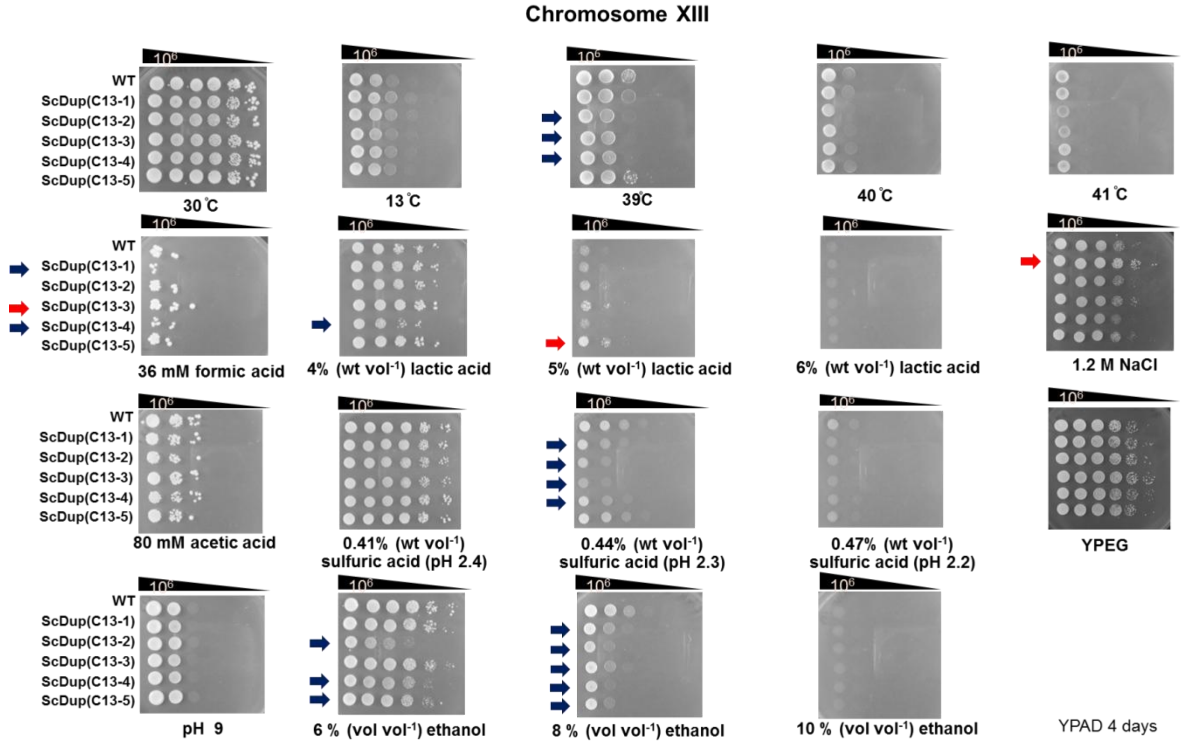
Chromosome XIII
10^6
WT
ScDup(C13-1)
ScDup(C13-2)
ScDup(C13-3)
ScDup(C13-4)
ScDup(C13-5)
30 ℃
13 ℃
39℃
40 ℃
41 ℃
36 mM formic acid
4% (wt vol^-1) lactic acid
5% (wt vol^-1) lactic acid
6% (wt vol^-1) lactic acid
1.2 M NaCl
80 mM acetic acid
0.41% (wt vol^-1) sulfuric acid (pH 2.4)
0.44% (wt vol^-1) sulfuric acid (pH 2.3)
0.47% (wt vol^-1) sulfuric acid (pH 2.2)
YPEG
pH 9
6 % (vol vol^-1) ethanol
8 % (vol vol^-1) ethanol
10 % (vol vol^-1) ethanol
YPAD 4 days

n)

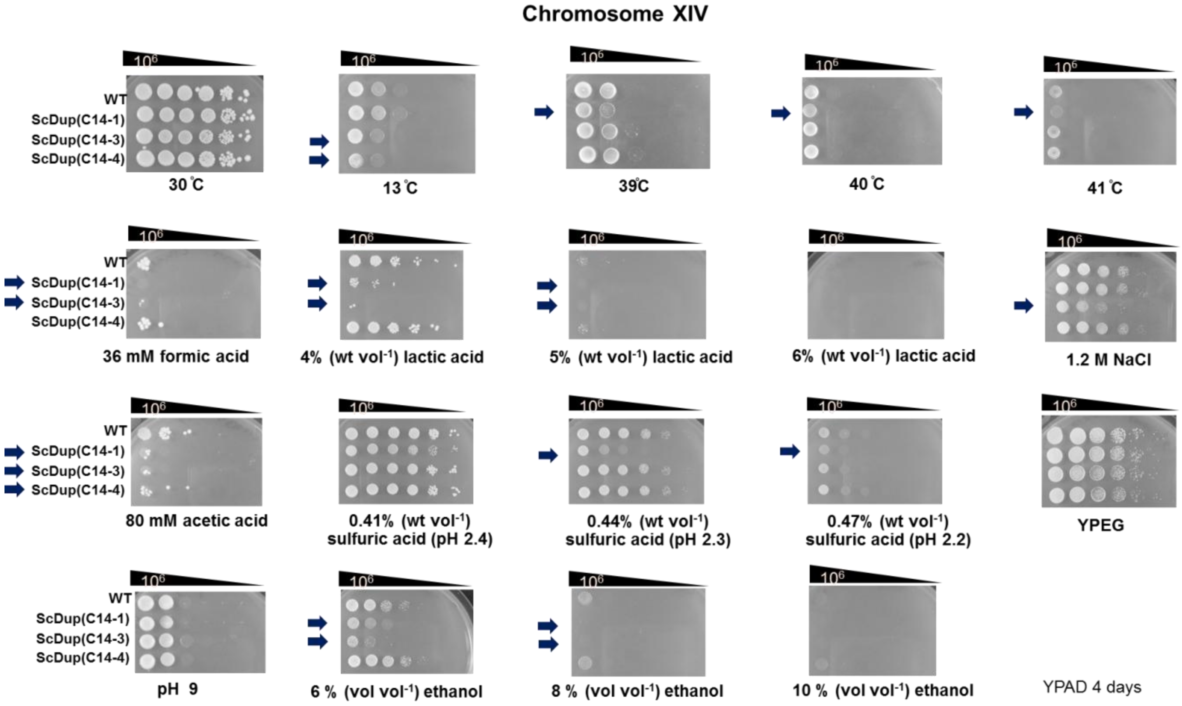
Chromosome XIV
WT
ScDup(C14-1)
ScDup(C14-3)
ScDup(C14-4)
30 ℃
13 ℃
39℃
40 ℃
41 ℃
36 mM formic acid
4% (wt vol-1) lactic acid
5% (wt vol-1) lactic acid
6% (wt vol-1) lactic acid
1.2 M NaCl
80 mM acetic acid
0.41% (wt vol-1) sulfuric acid (pH 2.4)
0.44% (wt vol-1) sulfuric acid (pH 2.3)
0.47% (wt vol-1) sulfuric acid (pH 2.2)
YPEG
pH 9
6 % (vol vol-1) ethanol
8 % (vol vol-1) ethanol
10 % (vol vol-1) ethanol
YPAD 4 days

o)

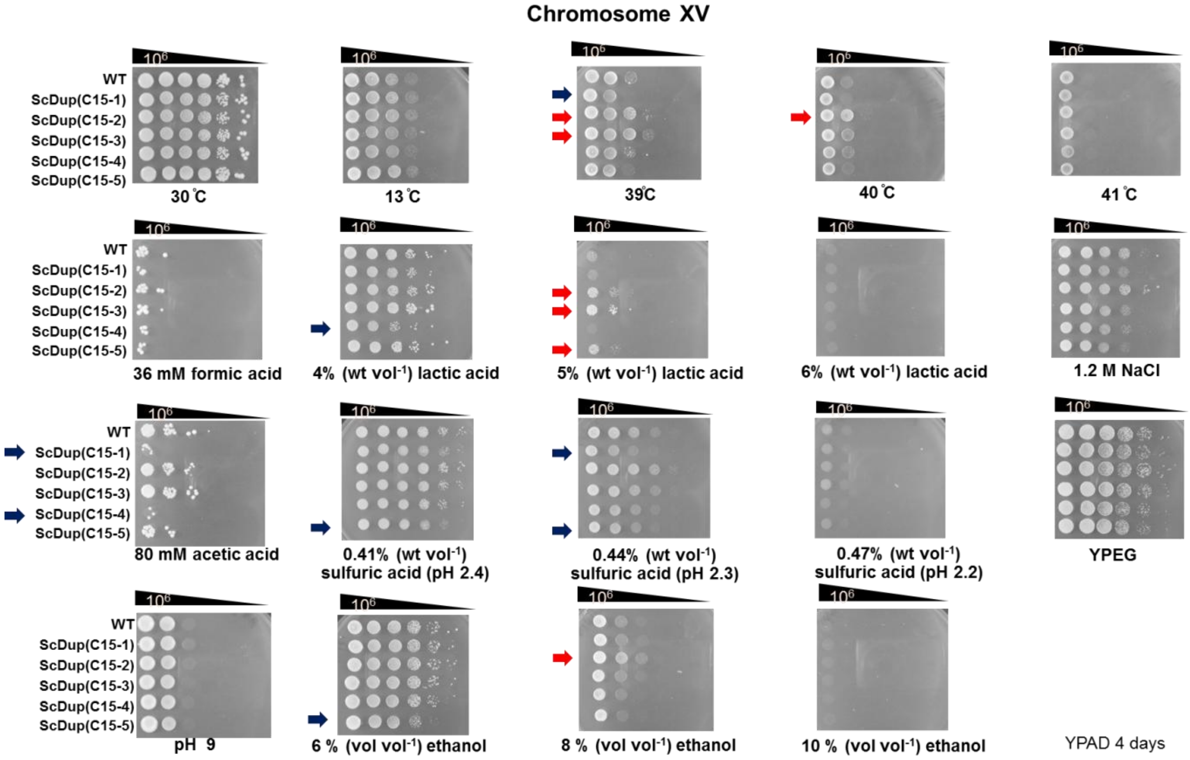
Chromosome XV
WT
ScDup(C15-1)
ScDup(C15-2)
ScDup(C15-3)
ScDup(C15-4)
ScDup(C15-5)
30 ℃
13 ℃
39℃
40 ℃
41 ℃
36 mM formic acid
4% (wt vol-1) lactic acid
5% (wt vol-1) lactic acid
6% (wt vol-1) lactic acid
1.2 M NaCl
80 mM acetic acid
0.41% (wt vol-1) sulfuric acid (pH 2.4)
0.44% (wt vol-1) sulfuric acid (pH 2.3)
0.47% (wt vol-1) sulfuric acid (pH 2.2)
YPEG
pH 9
6 % (vol vol-1) ethanol
8 % (vol vol-1) ethanol
10 % (vol vol-1) ethanol
YPAD 4 days

p)

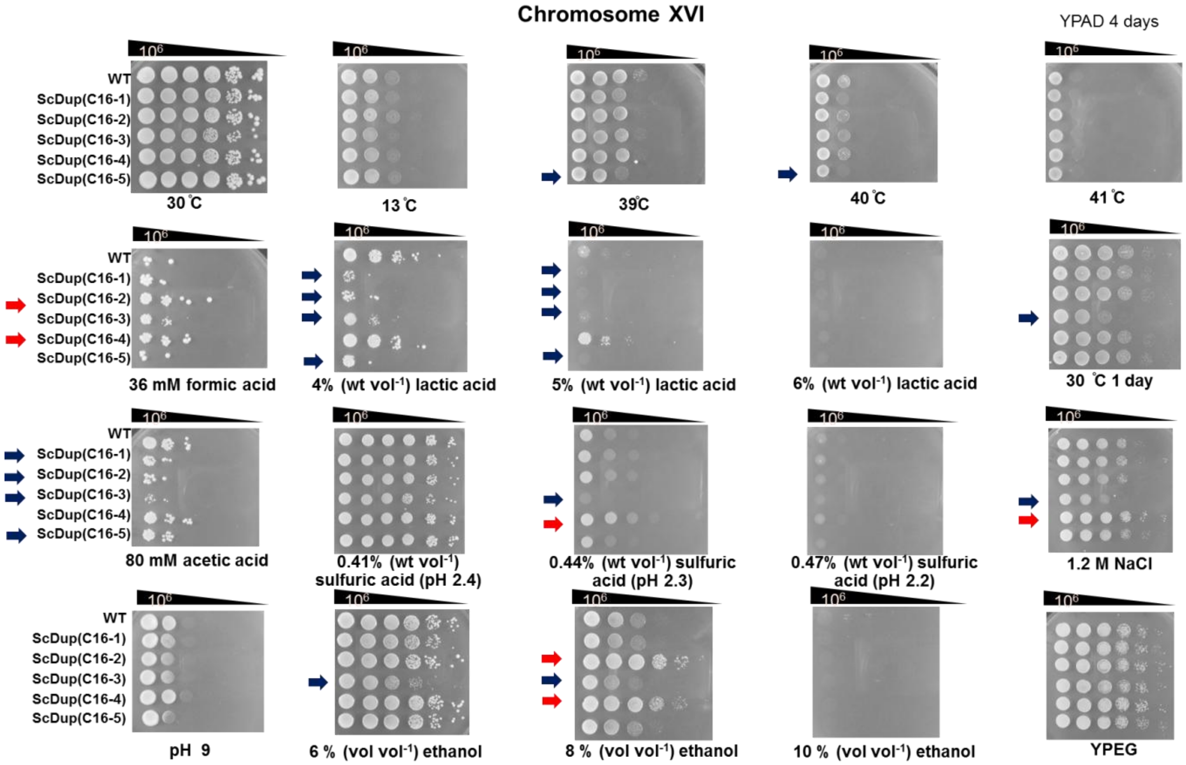
Chromosome XVI
YPAD 4 days
10^6
WT
ScDup(C16-1)
ScDup(C16-2)
ScDup(C16-3)
ScDup(C16-4)
ScDup(C16-5)
30 ℃
13 ℃
39 ℃
40 ℃
41 ℃
36 mM formic acid
4% (wt vol-1) lactic acid
5% (wt vol-1) lactic acid
6% (wt vol-1) lactic acid
30 ℃ 1 day
80 mM acetic acid
0.41% (wt vol-1) sulfuric acid (pH 2.4)
0.44% (wt vol-1) sulfuric acid (pH 2.3)
0.47% (wt vol-1) sulfuric acid (pH 2.2)
1.2 M NaCl
pH 9
6 % (vol vol-1) ethanol
8 % (vol vol-1) ethanol
10 % (vol vol-1) ethanol
YPEG

Supplementary Figure 4. Analysis of the relationship between segmental duplication and phenotype using a chromosome loss strategy.

Segmental aneuploid strains were induced to lose their additional chromosome and were then examined phenotypically. ΔCx-y indicates a derivative strains of ScDup(Cx-y) which has lost the duplicated chromosome. x represents chromosome number and y represents chromosome region. "+" and "-" means resistant phenotype and sensitive phenotype, respectively.

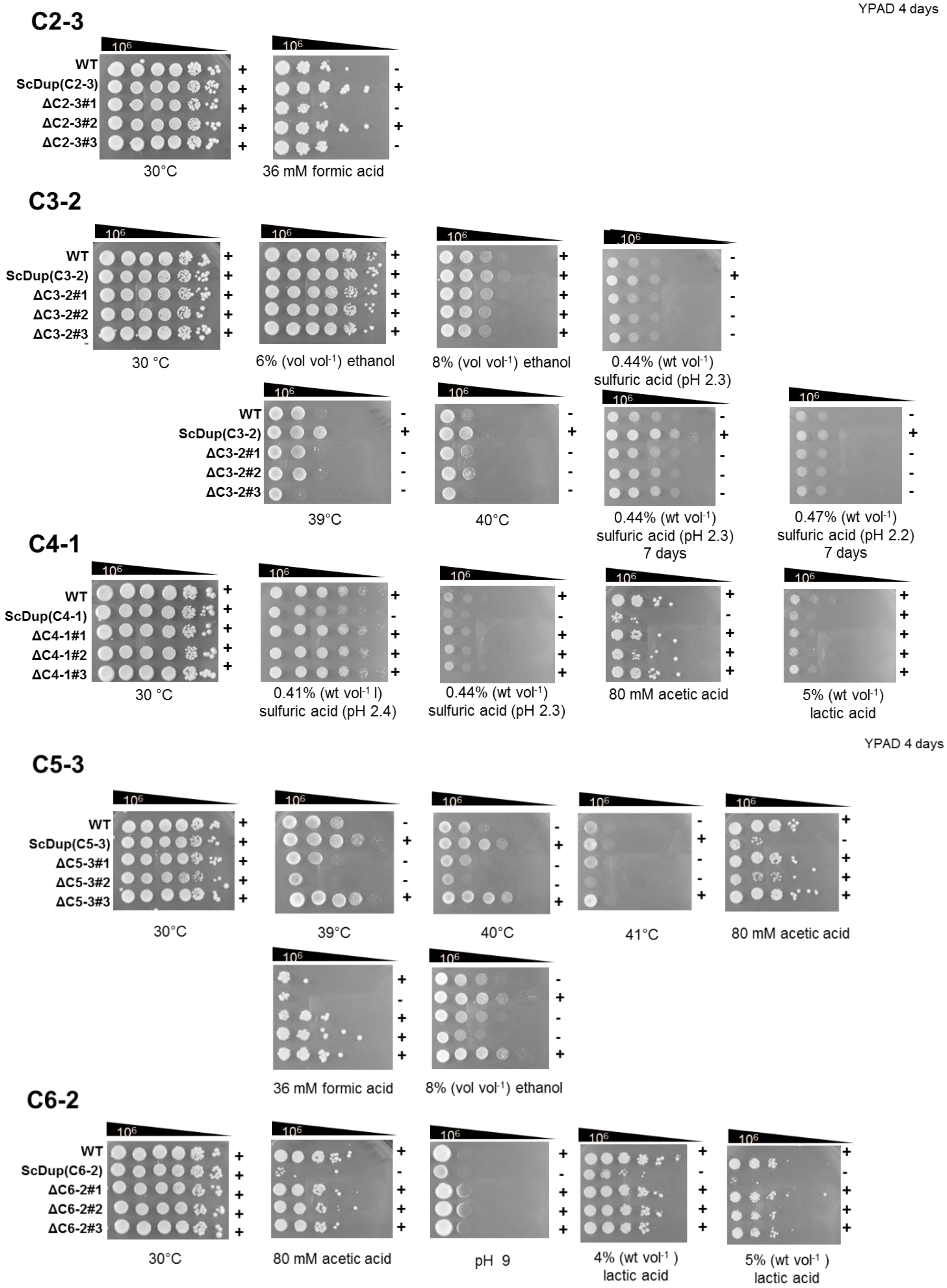
YPAD 4 days
C2-3
10^6
WT
ScDup(C2-3)
ΔC2-3#1
ΔC2-3#2
ΔC2-3#3
+ + + + +
- + - + -
30°C
36 mM formic acid
C3-2
WT
ScDup(C3-2)
ΔC3-2#1
ΔC3-2#2
ΔC3-2#3
30 °C
6% (vol vol-1) ethanol
8% (vol vol-1) ethanol
0.44% (wt vol-1) sulfuric acid (pH 2.3)
39°C
40°C
0.44% (wt vol-1) sulfuric acid (pH 2.3) 7 days
0.47% (wt vol-1) sulfuric acid (pH 2.2) 7 days
C4-1
WT
ScDup(C4-1)
ΔC4-1#1
ΔC4-1#2
ΔC4-1#3
30 °C
0.41% (wt vol-1 l) sulfuric acid (pH 2.4)
0.44% (wt vol-1) sulfuric acid (pH 2.3)
80 mM acetic acid
5% (wt vol-1) lactic acid
YPAD 4 days
C5-3
WT
ScDup(C5-3)
ΔC5-3#1
ΔC5-3#2
ΔC5-3#3
30°C
39°C
40°C
41°C
80 mM acetic acid
36 mM formic acid
8% (vol vol-1) ethanol
C6-2
WT
ScDup(C6-2)
ΔC6-2#1
ΔC6-2#2
ΔC6-2#3
30°C
80 mM acetic acid
pH 9
4% (wt vol-1) lactic acid
5% (wt vol-1) lactic acid

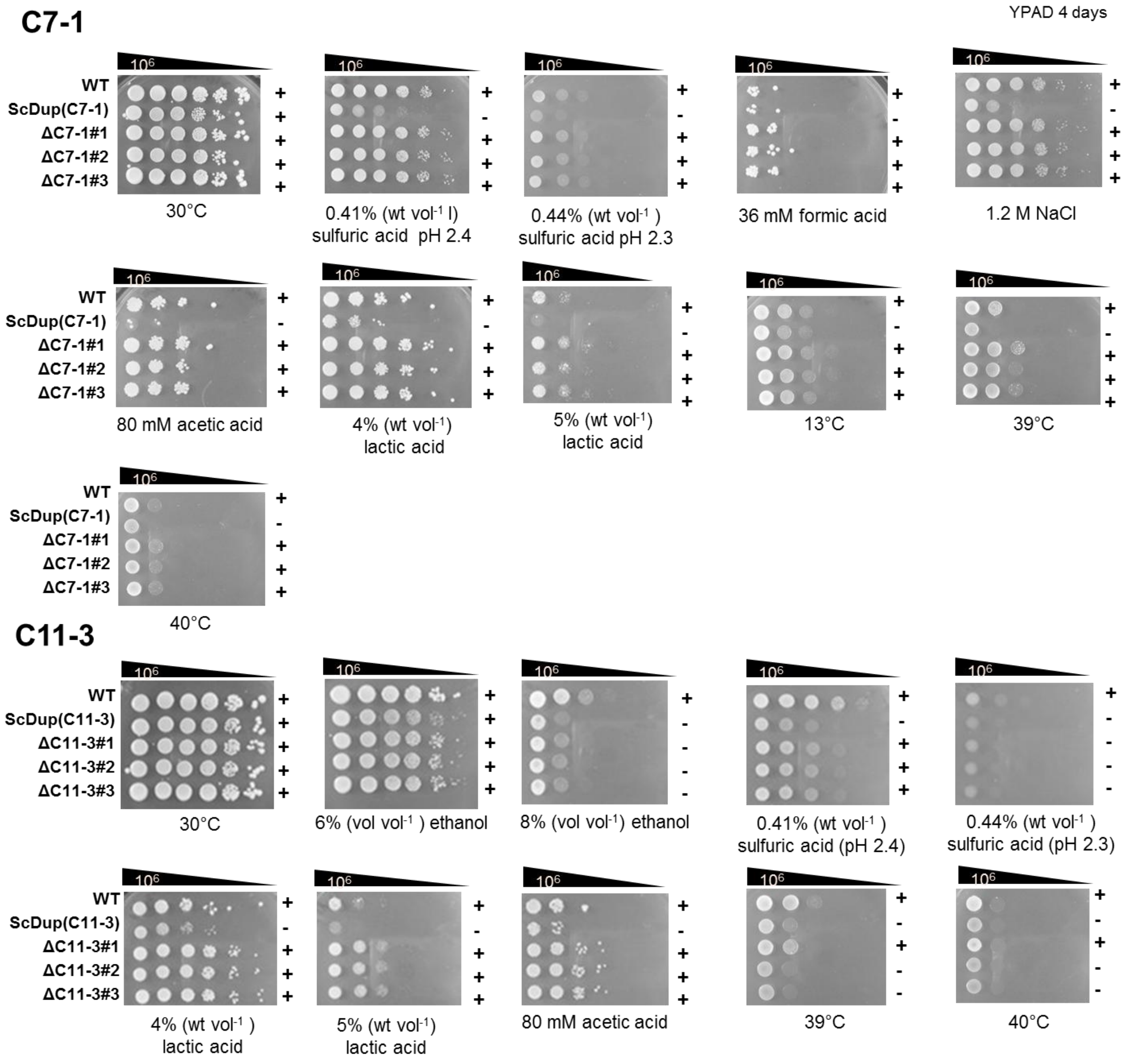
C7-1
YPAD 4 days
10^6
WT
ScDup(C7-1)
ΔC7-1#1
ΔC7-1#2
ΔC7-1#3
30°C
0.41% (wt vol^{-1} l) sulfuric acid pH 2.4
0.44% (wt vol^{-1}) sulfuric acid pH 2.3
36 mM formic acid
1.2 M NaCl
80 mM acetic acid
4% (wt vol^{-1}) lactic acid
5% (wt vol^{-1}) lactic acid
13°C
39°C
40°C
C11-3
WT
ScDup(C11-3)
ΔC11-3#1
ΔC11-3#2
ΔC11-3#3
30°C
6% (vol vol^{-1}) ethanol
8% (vol vol^{-1}) ethanol
0.41% (wt vol^{-1}) sulfuric acid (pH 2.4)
0.44% (wt vol^{-1}) sulfuric acid (pH 2.3)
4% (wt vol^{-1}) lactic acid
5% (wt vol^{-1}) lactic acid
80 mM acetic acid
39°C
40°C

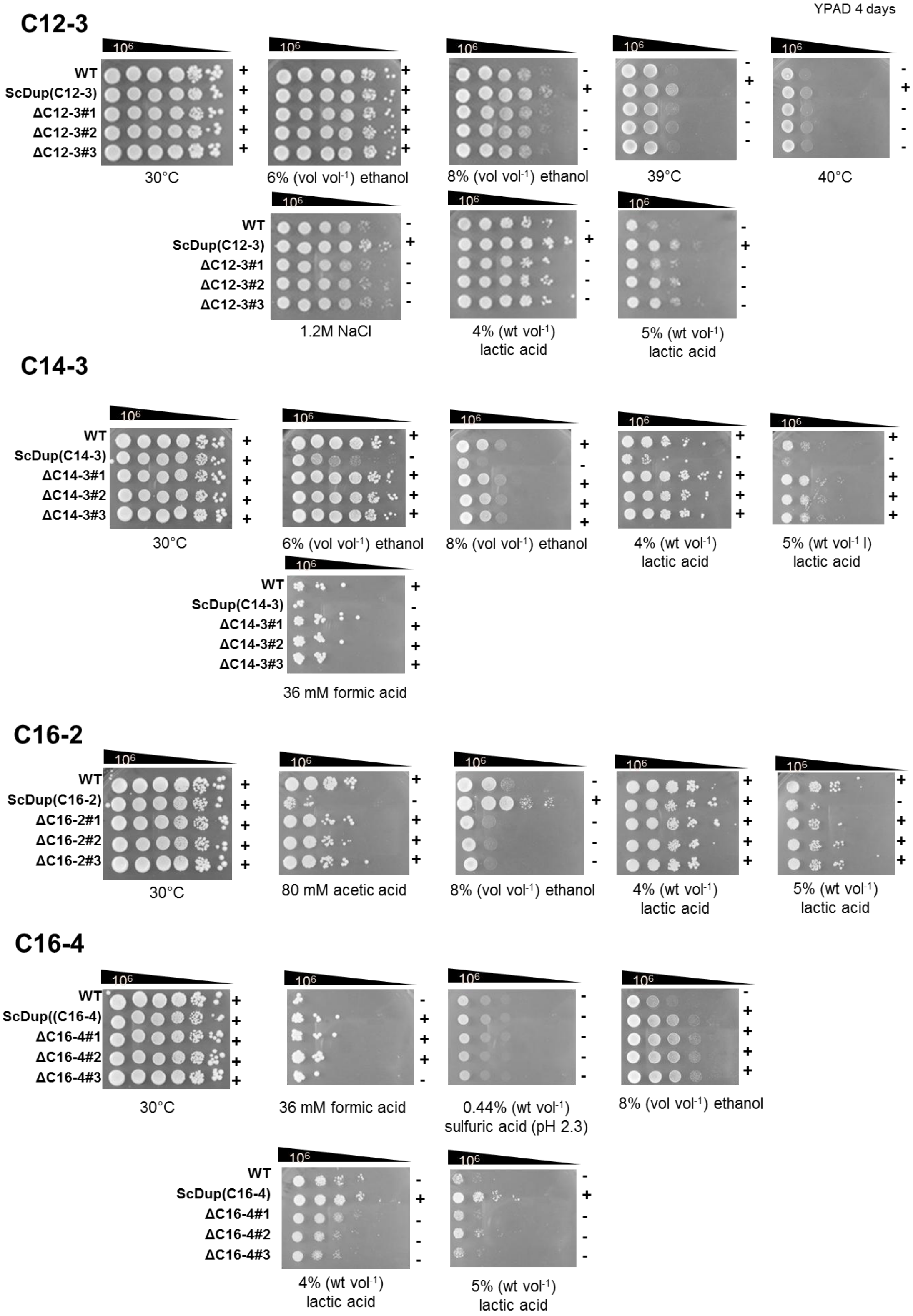
YPAD 4 days
C12-3
10^6
WT
ScDup(C12-3)
ΔC12-3#1
ΔC12-3#2
ΔC12-3#3
30°C
6% (vol vol-1) ethanol
8% (vol vol-1) ethanol
39°C
40°C
1.2M NaCl
4% (wt vol-1) lactic acid
5% (wt vol-1) lactic acid
C14-3
WT
ScDup(C14-3)
ΔC14-3#1
ΔC14-3#2
ΔC14-3#3
30°C
6% (vol vol-1) ethanol
8% (vol vol-1) ethanol
4% (wt vol-1) lactic acid
5% (wt vol-1 l) lactic acid
36 mM formic acid
C16-2
WT
ScDup(C16-2)
ΔC16-2#1
ΔC16-2#2
ΔC16-2#3
30°C
80 mM acetic acid
8% (vol vol-1) ethanol
4% (wt vol-1) lactic acid
5% (wt vol-1) lactic acid
C16-4
WT
ScDup((C16-4)
ΔC16-4#1
ΔC16-4#2
ΔC16-4#3
30°C
36 mM formic acid
0.44% (wt vol-1) sulfuric acid (pH 2.3)
8% (vol vol-1) ethanol
ScDup(C16-4)
4% (wt vol-1) lactic acid
5% (wt vol-1) lactic acid

Supplementary Figure 5. PFGE analysis of segmental aneuploid strains and derivative strains that had lost the duplicated chromosome

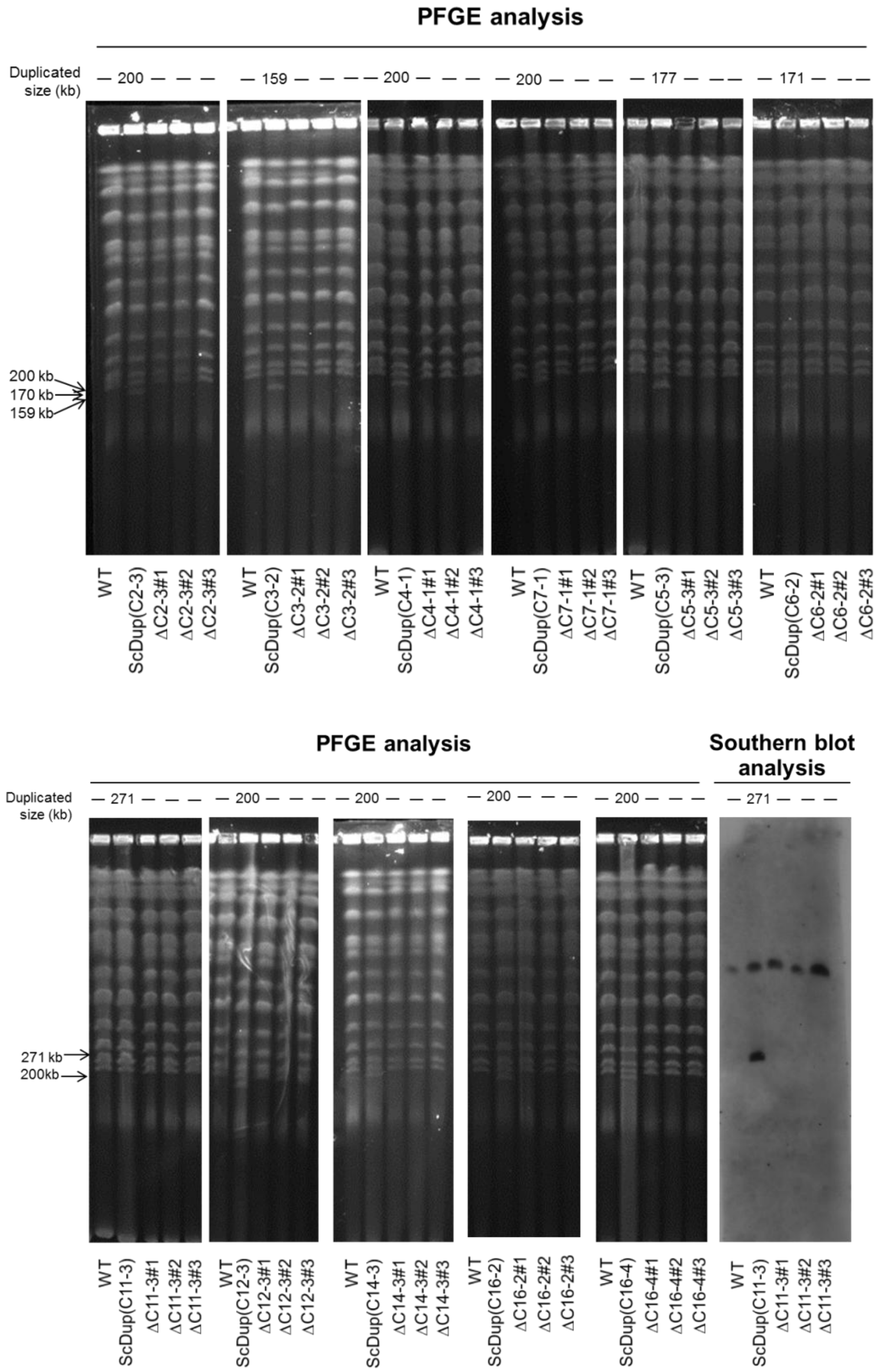

Supplementary Table 1. Growth of segmental aneuploids for chromosomes I to XVI under various stresses

Chr.	Chromosome region[a]	Duplication length (kb)	Number of genes	Phenotype[b]																										
				YPAD				YPAD				YPAD				YPAD					YPAD		YPAD		YPAD		YPAD	YP+ 3% glycerol	YPAD	pH 9
				0% lactic acid	4% lactic acid	5% lactic acid	6% lactic acid	0% ethanol	6% ethanol	8% ethanol	10% ethanol	0% sulfuric acid	0.41% sulfuric acid pH 2.4	0.44% sulfuric acid pH 2.3	0.47% sulfuric acid pH 2.2	30°C	13°C	39°C	40°C	41°C	0 mM acetic acid	80 mM acetic acid	0 mM formic acid	36 mM formic acid	0 M NaCl	1.2 M NaCl				
BY4742		0	-	++	+	-	-	++	+	+	-	++	+	+	+	++	+	+	+	+	++	+	++	+	++	+	++	++	++	+
I	C1-1	100	65	++	-	-	-	++	+	+	-	++	+	++	+	++	+	+	+	+	++	-	++	+	++	+	++	++	++	+
	C1-2	130	85	++	+	-	-	++	-	+	-	++	-	-	-	++	+	-	-	-	++	-	++	+	++	+	++	++	++	+
II	C2-1	200	137	++	--	--	-	++	+	-	-	++	+	-	-	++	+	+	+	+	++	-	++	+	++	+	++	++	++	+
	C2-2	200	128	++	-	--	-	++	+	--	-	++	+	-	-	++	+	+	+	+	++	-	++	++	++	+	++	-	++	+
	C2-3	200	124	++	+	+	-	++	+	+	-	++	+	+	+	++	+	+	+	+	++	++	++	+++	++	++	++	++	++	+
	C2-4	213	142	++	--	--	-	++	+	--	-	++	+	-	-	++	-	-	+	+	++	-	++	-	++	+	++	++	++	+
III	C3-1	158	139	++	+	-	-	++	++	++	-	++	+	++	++	++	+	++	+	+	++	+	++	+	++	++	++	++	++	+
	C3-2	159	110	++	+	-	-	++	++	++	-	++	+	++	++	++	+	++	+	+	++	+	++	+	++	+	++	++	++	+
IV	C4-1	200	119	++	+	++	-	++	+	+	-	++	+	-	-	++	+	+	+	+	++	-	++	+	++	+	++	++	++	+
	C4-3	200	140	++	-	-	-	++	+	-	-	++	+	+	+	++	+	-	-	-	++	+	++	+	++	+	++	++	++	+
	C4-6	200	121	++	-	-	-	++	+	+	-	++	+	+	+	++	+	+	+	+	++	+	++	+	++	+	++	++	++	+
	C4-8	130	89	++	+	--	-	++	+	+	-	++	-	--	-	++	+	-	-	-	++	++	++	+	++	+	++	++	++	-
V	C5-1	200	146	++	+	-	-	++	+	+	-	++	+	+	+	++	+	-	-	+	++	+	++	-	++	+	++	++	++	-
	C5-2	200	143	++	+	-	-	++	+	-	-	++	+	-	+	++	+	-	-	+	++	-	++	+	++	-	++	-	++	+
	C5-3	177	127	++	+	-	-	++	+	++	-	++	+	+	+	++	+	++	++	++	++	-	++	-	++	+	++	++	++	+
VI	C6-2	171	128	++	-	-	-	++	+	+	-	++	+	+	+	++	+	+	+	+	++	-	++	+	++	+	++	++	++	-
VII	C7-1	200	125	++	--	-	-	++	+	+	-	++	+	-	+	++	-	-	-	+	++	--	++	-	++	-	++	++	++	+
	C7-2	200	128	++	+	-	-	++	-	+	-	++	+	-	+	++	+	+	+	+	++	-	++	++	++	+	++	++	++	+
	C7-3	200	154	++	-	-	-	++	+	+	-	++	+	+	+	++	+	+	+	+	++	-	++	++	++	+	++	++	++	+
	C7-5	290	181	++	+	-	-	++	++	++	-	++	+	+	+	++	+	++	++	++	++	-	++	-	++	+	++	++	++	+
VIII	C8-1	200	146	++	+	-	-	++	+	+	-	++	+	+	+	++	+	+	+	+	++	-	++	+	++	+	++	++	++	+
	C8-3	160	99	++	+	--	-	++	+	+	-	++	+	+	+	++	+	+	+	+	++	-	++	-	++	+	++	++	++	+

Chr.	Chromosome region[a]	Duplication length (kb)	Number of genes	Phenotype[b]																										
				YPAD				YPAD				YPAD				YPAD					YPAD		YPAD		YPAD					
				0% lactic acid	4% lactic acid	5% lactic acid	6% lactic acid	0% ethanol	6% ethanol	8% ethanol	10% ethanol	0% sulfuric acid	0.41% sulfuric acid pH 2.4	0.44% sulfuric acid pH 2.3	0.47% sulfuric acid pH 2.2	30°C	13°C	39°C	40°C	41°C	0 mM acetic acid	80 mM acetic acid	0 mM formic acid	36 mM formic acid	0 M NaCl	1.2 M NaCl	YPAD	YP+ 3% glycerol	YPAD	pH 9
IX	C9-1	200	116	++	+	-	-	++	+	+	-	++	+	-	+	++	+	-	-	+	++	+	++	-	++	+	++	++	++	+
	C9-2	240	175	++	++	++	-	++	+	-	-	++	+	+	+	++	+	-	-	+	++	+	++	+	++	+	++	++	++	+
X	C10-1	200	131	++	-	--	-	++	-	--	-	++	--	+	+	++	+	-	+	+	++	-	++	+	++	+	++	++	++	+
	C10-2	200	130	++	+	-	-	++	+	+	-	++	-	+	+	++	+	++	++	++	++	--	++	-	++	+	++	-	++	+
	C10-3	200	142	++	+	-	-	++	+	-	-	++	+	+	+	++	+	-	+	+	++	-	++	-	++	-	++	++	++	+
	C10-4	150	87	++	+	-	-	++	+	+	-	++	+	+	+	++	+	+	+	+	++	+	++	+	++	+	++	++	++	+
XI	C11-1	200	116	++	+	-	-	++	-	+	-	++	+	+	+	++	+	+	+	+	++	-	++	+	++	-	++	++	++	+
	C11-3	267	153	++	-	--	-	++	--	--	-	++	-	--	-	++	+	-	-	+	++	-	++	+	++	+	++	++	++	+
XII	C12-1	250	146	++	++	++	-	++	+	+	-	++	+	+	+	++	+	-	+	+	++	-	++	+	++	+	++	++	++	+
	C12-2	200	117	++	-	--	-	++	+	+	-	++	+	-	+	++	+	++	+	+	++	-	++	+	++	+	++	-	++	+
	C12-3	200	140	++	++	++	-	++	+	++	-	++	+	+	+	++	+	++	++	+	++	+	++	+	++	++	++	++	++	+
	C12-4	200	139	++	+	-	-	++	+	+	-	++	+	-	+	++	+	-	+	+	++	+	++	+	++	+	++	++	++	+
	C12-5	200	115	++	+	-	-	++	+	+	-	++	+	+	+	++	+	-	+	+	++	-	++	-	++	+	++	++	++	+
XIII	C13-1	200	130	++	+	-	-	++	+	-	-	++	+	-	+	++	+	+	+	+	++	+	++	-	++	++	++	++	++	+
	C13-2	200	141	++	+	-	-	++	--	-	-	++	+	-	+	++	+	-	+	+	++	+	++	+	++	+	++	++	++	+
	C13-3	200	133	++	+	-	-	++	+	-	-	++	+	-	+	++	+	-	+	+	++	+	++	++	++	+	++	++	++	+
	C13-4	200	120	++	+	-	-	++	-	-	-	++	+	-	+	++	+	-	+	+	++	+	++	-	++	+	++	++	++	+
	C13-5	120	83	++	+	+	-	++	-	-	-	++	+	+	+	++	+	+	+	+	++	+	++	+	++	+	++	++	++	+
XIV	C14-1	200	122	++	--	--	-	++	--	--	-	++	+	-	-	++	+	-	-	-	++	-	++	-	++	+	++	++	++	+
	C14-3	200	130	++	----	--	-	++	---	--	-	++	+	+	+	++	+	+	+	+	++	-	++	-	++	-	++	++	++	+
	C14-4	184	118	++	+	-	-	++	+	+	-	++	+	+	+	++	-	+	+	+	++	-	++	+	++	+	++	++	++	+
XV	C15-1	200	125	++	+	-	-	++	+	+	-	++	+	-	+	++	+	-	+	+	++	-	++	+	++	+	++	++	++	+
	C15-2	200	135	++	+	+	-	++	+	++	-	++	+	+	+	++	+	++	++	+	++	+	++	+	++	+	++	++	++	+
	C15-3	200	128	++	+	+	-	++	+	+	-	++	+	+	+	++	+	++	+	+	++	+	++	+	++	+	++	++	++	+
	C15-4	200	134	++	-	-	-	++	+	+	-	++	+	+	+	++	+	+	+	+	++	-	++	+	++	+	++	++	++	+

Chr.	Chromosome region[a]	Duplication length (kb)	Number of genes	Phenotype[b]																										
				YPAD				YPAD				YPAD				YPAD					YPAD		YPAD		YPAD					
				0% lactic acid	4% lactic acid	5% lactic acid	6% lactic acid	0% ethanol	6% ethanol	8% ethanol	10% ethanol	0% sulfuric acid	0.41% sulfuric acid pH 2.4	0.44% sulfuric acid pH 2.3	0.47% sulfuric acid pH 2.2	30°C	13°C	39°C	40°C	41°C	0 mM acetic acid	80 mM acetic acid	0 mM formic acid	36 mM formic acid	0 M NaCl	1.2 M NaCl	YPAD	YP+ 3% glycerol	YPAD	pH 9
	C15-5	290	176	++	+	+	-	++	-	+	-	++	+	+	+	++	+	+	+	+	++	+	++	+	++	+	++	++	++	+
XVI	C16-1	200	124	++	---	--	-	++	+	+	-	++	+	+	+	++	+	+	+	+	++	-	++	+	++	+	++	++	++	+
	C16-2	200	116	++	---	--	-	++	+	++<br>+	-	++	+	+	+	++	+	+	+	+	++	-	++	++	++	+	++	++	++	+
	C16-3	200	124	++	--	--	-	++	-	+	-	++	+	--	+	++	+	+	+	+	++	-	++	+	++	-	++	++	++	+
	C16-4	200	136	++	+	+	-	++	+	++<br>+	-	++	+	++	+	++	+	+	+	+	++	+	++	++	++	++	++	++	++	+
	C16-5	148	112	++	---	--	-	++	+	+	-	++	+	+	+	++	+	-	-	+	++	-	++	+	++	+	++	++	++	+

*a: Chr.N x-y : Chr. N represents chromosome number, x represents first nucleotide number of chromosomal region and y represents last nucleotide number of chromosomal region.

b: + means resistant phenotype and - means sensitive phenotype

Supplementary Table 2. Stress sensitive and resistant phenotypes of segmental aneuploids for chromosomes I to XVI

Strain name	Sensitive phenotype[a]	Resistance phenotype[a]
ScDup(C1-1)	4%L, A	S pH 2.3, S pH 2.2
ScDup(C1-2)	S pH 2.3, S pH 2.2, 6%E, A , 39°C, 40°C, 41°C	-
ScDup(C2-1)	4%L, 5%L, S pH 2.3, S pH 2.2, 8%E, A	-
ScDup(C2-2)	4%L, 5%L, S pH 2.3, S pH 2.2, 8%E, G, N, A	F
ScDup(C2-3)	-	F, N, A
ScDup(C2-4)	13 °C, 4%L, 5%L, S pH 2.4, S pH 2.3, S pH 2.2, 8%E, F, A, 39°C	-
ScDup(C3-1)	-	S pH 2.3, S pH 2.2, 6%E, 8%E, N 39°C, 40°C
ScDup(C3-2)	-	S pH 2.3, S pH 2.2, 6%E, 8%E, 39°C, 40°C
ScDup(C4-1)	S pH 2.3, S pH 2.2, A	5%L
ScDup(C4-2)	-	-
ScDup(C4-3)	4%L, 39°C, 40°C, 41°C	-
ScDup(C4-4)	-	-
ScDup(C4-5)	-	-
ScDup(C4-6)	4%L, pH 9	-
ScDup(C4-7)	-	-
ScDup(C4-8)	5%L, S pH 2.3, S pH 2.2, pH 9, 39°C, 40°C, 41°C	A
ScDup(C5-1)	F, 39°C, 40°C	-
ScDup(C5-2)	S pH 2.3, 8%E, G, N, A, 39°C, 40°C	-
ScDup(C5-3)	F, A	39°C, 40°C, 41 °C, 8%E
ScDup(C6-1)	-	-
ScDup(C6-2)	4%L, 5%L, A, pH 9	-
ScDup(C7-1)	13 °C, 4%L, 5%L, S pH 2.3, F, N, A, 39°C, 40°C	-
ScDup(C7-2)	S pH 2.3, 6%E, A	F
ScDup(C7-3)	4%L, 5%L, A	F
ScDup(C7-4)	-	-
ScDup(C7-5)	F, A	6%E, 8%E, N, 39°C, 40°C, 41 °C
ScDup(C8-1)	A	-
ScDup(C8-2)	-	-
ScDup(C8-3)	5%L, F, A	-
ScDup(C9-1)	S pH 2.3, F, 39°C, 40°C	-
ScDup(C9-2)	8%E, 39°C, 40°C	4%L, 5%L

Strain name	Sensitive phenotype[a]	Resistance phenotype[a]
ScDup(C10-1)	4%L, 5%L, S pH 2.4, S pH 2.3, 6%E, 8%E, A, 39°C	-
ScDup(C10-2)	F, G, A	39°C, 40°C, 41°C
ScDup(C10-3)	8%E, F, N, A, 39°C	-
ScDup(C10-4)	-	-
ScDup(C11-1)	S pH 2.3, 6%E, 8%E, N, A	-
ScDup(C11-2)	-	-
ScDup(C11-3)	4%L, 5%L, S pH 2.4, S pH 2.3, S pH 2.2, 6%E,8%E, A, 39°C, 40°C	-
ScDup(C12-1)	13 °C, A, 39°C	4%L, 5%L
ScDup(C12-2)	4%L, 5%L, S pH 2.3, G, A	39°C
ScDup(C12-3)	-	4%L, 5%L, 8%E, N, 39°C, 40°C
ScDup(C12-4)	S pH 2.3, 39°C	-
ScDup(C12-5)	F, A, 39°C	-
ScDup(C13-1)	S pH 2.3, 8%E, F	N
ScDup(C13-2)	S pH 2.3, 6%E, 8%E, 39°C	-
ScDup(C13-3)	S pH 2.3, 8%E, 39°C	F
ScDup(C13-4)	4%L, S pH 2.3, 6%E, 8%E, F, 39°C	-
ScDup(C13-5)	6%E, 8%E	5%L
ScDup(C14-1)	4%L, 5%L, S pH 2.3, S pH 2.2, 6%E,8%E, F, A, 39°C, 40°C, 41°C	-
ScDup(C14-2)	-	-
ScDup(C14-3)	13 °C, 4%L, 5%L, 6%E, 8%E, F, N, A	-
ScDup(C14-4)	13 °C, A	-
ScDup(C15-1)	S pH 2.3, A, 39°C	-
ScDup(C15-2)	-	5%L, 8%E, 39°C, 40°C
ScDup(C15-3)	-	5%L, 39°C
ScDup(C15-4)	4%L, A	-
ScDup(C15-5)	6%E	5%L
ScDup(C16-1)	4%L, 5%L, A	-
ScDup(C16-2)	4%L, 5%L, A	8%E, F
ScDup(C16-3)	41 °C,4%L, 5%L, S pH 2.3, 6%E, 8%E, N, A	-
ScDup(C16-4)	-	5%L, S pH 2.3, 8%E, F, N
ScDup(C16-5)	4%L, 5%L, A, 39°C, 40°C	-

a: 4%L; 4% (wt vol^{-1}) lactic acid, 5%L; 5% (wt vol^{-1}) lactic acid, S pH 2.4; 0.41% (wt vol^{-1}) sulfuric acid pH 2.4, S pH 2.3; 0.44% (wt vol^{-1}) sulfuric acid pH 2.3, S pH 2.2; 0.47% (wt vol^{-1}) sulfuric acid pH 2.2, 6%E; 6% (vol vol^{-1}) ethanol, 8%E; 8% (vol vol^{-1}) ethanol, F; 36 mM formic acid, N; 1.2 M NaCl, G; YPEG, A; 80 mM acetic acid

Supplementary Table 3. Genes located in 50 kb unduplicated sub-regions whose overexpression is associated with cell lethality or abnormalities in cell cycle progression or the actin skeleton

Chromosome region	Subregion	Gene	Gene Systematic Name	Chromosome location	Phenotype	Strain Background	Reference
C4-4	S2	*BMH2*	*YDR099W*	Chr.IV 653,607-654,428	actin cytoskeleton morphology: abnormal	Other	Roth *et al.*[57]
C4-4	S2	*PDS1*	*YDR113C*	Chr.IV 680,617-680,496	fitness defect	S288C	Douglas *et al.*[47]
C4-4	S2	*PDS1*	*YDR113C*	Chr.IV 680,617-680,496	cell cycle progression: abnormal	W303	Stevenson *et al.*[49]
C4-4	S2	*PDS1*	*YDR113C*	Chr.IV 681,617-680,496	inviable	S288C	Sopko *et al.*[45]
C4-4	S2	*KIN1*	*YDR122W*	Chr.IV 694,700 – 697,894	Toxic gene		
C4-4	S2	*INO2*	*YDR123C*	Chr.IV 699,468-698,554	inviable	S288C	Sopko *et al.*[45]
C4-7	S4	*SPP41*	*YDR464W*	Chr.IV 1,388,872 – 1,393,179	fitness defect	S288C	Douglas *et al.*[47]
C4-7	S4	*STP1*	*YDR463W*	ChrIV 1,386,816 – 1,388,375	fitness defect	S288C	Douglas *et al.*[47]
C4-7	S4	*TLG1*	*YDR468C*	Chr.IV 1,398,700-1,398,026	inviable	S288C	Sopko *et al.*[45]
C4-7	S4	*UGO1*	*YDR470C*	Chr.IV 1,401,214-1,399,706	inviable	S288C	Sopko *et al.*[45]
C6-1	S2	*ACT1*	*YFL039C*	Chr.VI 54,696-53,260	cell cycle progression: abnormal	S288C	Niu *et al.*[48]
C6-1	S2	*ACT1*	*YFL039C*	Chr.VI 54,696-53,260	cell cycle progression: abnormal	W303	Stevenson *et al.*[49]
C6-1	S2	*ACT1*	*YFL039C*	Chr.VI 54,696-53,260	inviable	Other	Liu *et al.*[46]
C6-1	S2	*TUB2*	*YFL037W*	Chr.VI 56,336-57,709	fitness defect	S288C	Douglas *et al.*[47]
C6-1	S2	*TUB2*	*YFL037W*	Chr.VI 56,336-57,709	inviable	Other	Liu *et al.*[46]
C6-1	S2	*TUB2*	*YFL037W*	Chr.VI 56,336-57,709	cell cycle progression: abnormal	S288C	Niu *et al.*[48]

Chromosome region	Subregion	Gene	Gene Systematic Name	Chromosome location	Phenotype	Strain Background	Reference
C6-1	S2	*HAC1*	*YFL031W*	Chr.VI 75,179-76,147	cell cycle progression: abnormal	W303	Stevenson *et al.*[49]
C6-1	S2	*HAC1*	*YFL031W*	Chr.VI 75,179-76,147	actin cytoskeleton morphology: abnormal	S288C	Sopko *et al.*[45]
C6-1	S2	*FRS2*	*YFL022C*	Chr.VI 95,010-93,499	cell cycle progression: abnormal	S288C	Niu *et al.*[48]
C8-2	S3	*DMA1*	*YHR115C*	Chr.VIII 340,109 – 341,359	fitness defect	S288C	Douglas *et al.*[47]
C11-2	S2	*HSL1*	*YKL101W*	Chr.XI 248,920-253,476	inviable	S288C	Sopko *et al.*[45]
C11-2	S2	*HSL1*	*YKL101W*	Chr.XI 248,920-253,476	cell cycle progression: abnormal	S288C	Sopko *et al.*[45]
C11-2	S2	*YKL100C*	*YKL100C*	Chr.XI 253,697 – 255,460	fitness defect	S288C	Douglas *et al.*[47]
C11-2	S2	*MIF2*	*YKL089W*	Chr.XI 273,394 – 275,043	fitness defect	S288C	Douglas *et al.*[47]
C11-2	S2	*RRP14*	*YKL082C*	Chr.XI 281,025 – 282,329	fitness defect	S288C	Douglas *et al.*[47]
C11-2	S2	*DHR2*	*YKL078W*	Chr.XI 288,845-291,052	cell cycle progression: abnormal	S288C	Niu *et al.*[48]
C14-2	S4	*TOM22*	*YNL131W*	Chr.XIV 378,767-379,225	cell cycle progression: abnormal	W303	Stevenson *et al.*[49]
C14-2	S4	*SPC98*	*YNL126W*	Chr.XIV 387,227-389,767	cell cycle progression: abnormal	W303	Stevenson *et al.*[49]

Supplementary Table 4. Genes whose upper copy number limit is less than 3 (Makanae *et al*.[50])

Chromosome region	Locus name	Gene name	Chromosome location	Copy number limit
C4-1	*YDL192W*	*ARF1*	Chr.IV 116,321-116,866	1.0
C4-7	*YDR129C*	*SAC6*	Chr.IV 715,379-713,340	2.0
C5-2	*YER040W*	*GLN3*	Chr.V 229,795-231,987	1.5
C6-2	*YFL010C*	*WWM1*	Chr.VI 115,743-115,108	0.6
C6-1	*YFL037W*	*TUB2*	Chr.VI 56,336-57,709	2.7
C6-1	*YFL039C*	*ACT1*	Chr.VI 54,696-53,260	1.2
C6-2	*YFR028C*	*CDC14*	Chr.VI 210,068-208,413	0.9
C7-2	*YGL071W*	*AFT1*	Chr.VII 372,012-374,084	2.9
C7-5	*YGR159C*	*NSR1*	Chr.VII 807,656-806,412	1.7
C9-1	*YIL095W*	*PRK1*	Chr.IX 183,937-186,369	2.1
C10-1	*YJL164C*	*TPK1*	Chr.X 111,159-109,966	0.9
C11-2	*YKL042W*	*SPC42*	Chr.XI 358,475-359,566	1.8
C11-1	*YKL166C*	*TPK3*	Chr.XI 135,705-134,509	0.6
C13-2	*YML016C*	*PPZ1*	Chr.XIII 241,536-239,458	0.3
C14-4	*YNL016W*	*PUB1*	Chr.XIV 602,907-604,268	2.6
C15-2	*YOR008C*	*SLG1*	Chr.XV 342,414-341,278	2.6
C16-2	*YPL145C*	*KES1*	Chr.XVI 279,699-278,395	2.3
C16-2	*YPL154C*	*PEP4*	Chr.XVI 260,931-259,714	0.8
C16-1	*YPL203W*	*TPK2*	Chr.XVI 166,256-167,398	2.1
C16-3	*YPR008W*	*HAA1*	Chr.XVI 573,018-575,102	2.3
C16-4	*YPR080W*	*TEF1*	Chr.XVI 700,594-701,970	0.6
C16-5	*YPR173C*	*VPS4*	Chr.XVI 887,837-886,524	0.7

Supplementary Table 5. Genes located in duplicated chromosome regions whose overexpression cause sensitive or resistant phenotypes

Chromosome region	Observed phenotype in this study	Gene	Gene Systematic Name	Chromosome location	Phenotype in previous study	Reference
C3-1	resistance to 1.2M NaCl	*SAT4*	*YCR008W*	ChrIII 128,470-130,281	resistance to sodium chloride: increased	Mulet *et al.*[34]
C3-1	tolerance to 39°C and 40°C	*LRE1*	*YCL051W*	ChrIII 35,865-37,616	innate thermotolerance: increased	Versele & Thevelein[37]
C3-2	tolerance to 39°C and 40°C	*HCM1*	*YCR065W*	ChrIII 229,310-231,004	innate thermotolerance: increased	Maoz N, *et al.*[38]
C5-3	resistance to 8% (vol vol^{-1}) ethanol	*SPT15*	*YER148W*	ChrV 465,303-466,025	resistance to ethanol: increased	Yang *et al.*[35]
C12-3	resistance to 8% (vol vol^{-1}) ethanol	*RSA3*	*YLR221C*	ChrXII 579,024 -578,362	resistance to ethanol: increased	Anderson *et al.*[33]
C12-3	resistance to 1.2M NaCl	*RSA3*	*YLR221C*	ChrXII 579,024 -578,362	osmotic stress resistance: increased	Anderson *et al.*[33]
C15-3	tolerance to 39°C	*LSP1*	*YPL004C*	ChrXVI 551,657 - 550,632	innate thermotolerance: increased	Zhang *et al.*[36]

Supplementary Table 6. Plasmids used in this study.

Plasmid	Description	Duplicating module	Remarks
p3008	The *loxP-CgLEU2-loxP* module containing plasmid constructed by modifying pUG6	A fragment containing the 5'-$(C_4A_2)_6$-3' telomere seed sequence and the *CgLEU2* cassette	Sugiyama *et al.*[56]
p3009	The *loxP-CgHIS3-loxP* module containing plasmid constructed by modifying pUG6	A fragment containing the 5'-$(C_4A_2)_6$-3' telomere seed sequence and the *CgHIS3* cassette	Sugiyama *et al.*[56]
p3122	The *loxP-CgLEU2-CEN4-loxP* module containing plasmid constructed by modifying pUG6	A fragment containing the 5'-$(C_4A_2)_6$-3' telomere seed sequence	Sugiyama *et al.*[58]
p3276	*URA3* containing plasmid constructed by modifying pUG6	A fragment containing the 5'-$(C_4A_2)_6$-3' telomere seed sequence and the *URA3* cassette	Sugiyama *et al.*[58]
p3279	The *loxP-CgHIS3-H4ARS-loxP* module containing plasmid constructed by modifying pUG6	A fragment containing 5'-$(C_4A_2)_6$-3' telomere seed sequence, *CgHIS3* and the *H4ARS* cassette	NBRP, YGRC, Japan
YCp50	*URA3* centromeric plasmid whose length is 7.8 kb	-	Rose *et al.*[59]

Supplementary Table 7. Primers used for construction of segmental chromosome duplications of chromosomes I to XVI

Region name	Primer name	Nucleotide sequences (5'-3')
C1-1	C1-1-R-f	GGCACTAGTTCCCTTCTTAC
	C1-1-R-r	CTGCAGCGTACGAAGCTTCAGCTGGCGGCCCAGGGAGAGAAAGGCATTGG
C1-2	C1-2-L-f	CTGCAGCGTACGAAGCTTCAGCTGGCGGCCGAATGAGAAGTCGTGTCGTC
	C1-2-L-r	CCTTTAGTAGCTGTTGGGCT
C2-1	C2-1-R-f	TTACATGCGACACCAAGCAG
	C2-1-R-r	CTGCAGCGTACGAAGCTTCAGCTGGCGGCCGTCCTCCGAGGCAGGCCCTC
C2-2	C2-2-L-f	CTGCAGCGTACGAAGCTTCAGCTGGCGGCCTGAATGCAATTCGATACTCG
	C2-2-L-r	CAATCCAGTGATACCCGTGG
	C2-2-R-f	TATAAACGCGCTTGCGATCG
	C2-2-R-r	CTGCAGCGTACGAAGCTTCAGCTGGCGGCCTGGAGTTTTGAGTTCATCTG
C2-3	C2-3-L-f	CTGCAGCGTACGAAGCTTCAGCTGGCGGCCCACAGCATTTGATCTTGGTC
	C2-3-L-r	CGTGCAAGCAAAAGCATTTG
	C2-3-R-f	TCTCTGAGGGTTATCAAATG
	C2-3-R-r	CTGCAGCGTACGAAGCTTCAGCTGGCGGCCCGTGTGATGTGGACTGTTGC
C2-4	C2-4-L-f	CTGCAGCGTACGAAGCTTCAGCTGGCGGCCCTAACCCTTTGATGTCCGAC
	C2-4-L-r	CTTTTCTTCCCTCCAAGATC
C3-1	C3-1-R-f	CTGAGAGAATCCTCCTACGG
	C3-1-R-r	CTGCAGCGTACGAAGCTTCAGCTGGCGGCCATATCACGTTGTGAGCAGCC
C3-2	C3-2-L-f	CTGCAGCGTACGAAGCTTCAGCTGGCGGCCGGGATCGGGATATGGCTTTG
	C3-2-L-r	CGTGATACCGGGGGTTGAAG
C4-1	C4-1-R-f	AGGGCATCCATCCAACCATC
	C4-1-R-r	CTGCAGCGTACGAAGCTTCAGCTGGCGGCCGGCTTTGGAGGAGATATTTG
C4-2	C4-2-L-f	CTGCAGCGTACGAAGCTTCAGCTGGCGGCCGTGCTCTTCTTGTTAACCCC
	C4-2-L-r	GGCCGCAATTGACGACACAC
	C4-2-R-f	TCTTTTCATTATTGCTAGTA
	C4-2-R-r	CTGCAGCGTACGAAGCTTCAGCTGGCGGCCAAAGTAGTTCATGATGCGGG
C4-3	C4-3-L-f	CTGCAGCGTACGAAGCTTCAGCTGGCGGCCCCACTTAACAAGAAGATTAG
	C4-3-L-r	CATACTTGAACCACCTGAAA
	C4-3-R-f	TCGAGGACAAAAAGGCATAT
	C4-3-R-r	CTGCAGCGTACGAAGCTTCAGCTGGCGGCCGAGAATAAAATAGGTCAGGT
C4-4	C4-4-L-f	CTGCAGCGTACGAAGCTTCAGCTGGCGGCCAGGATTTTAATCTGTTGGAG
	C4-4-L-r	CCAACCAATATTACTGCTTT
	C4-4-R-f	CCGACCGAGTATTACTCAGT
	C4-4-R-r	CTGCAGCGTACGAAGCTTCAGCTGGCGGCCGAGTCATCCATATTGCAAAC
C4-5	C4-5-L-f	CTGCAGCGTACGAAGCTTCAGCTGGCGGCCCCAAAAGTTGCCTGTCCAAA
	C4-5-L-r	GAAGGCAAGGCTTACAGGCT
	C4-5-R-f	TTACGGTGGTTGCAAAGGGA
	C4-5-R-r	CTGCAGCGTACGAAGCTTCAGCTGGCGGCCAAAGAAGACTTCAATAAGTT
C4-6	C4-6-L-f	CTGCAGCGTACGAAGCTTCAGCTGGCGGCCGTTGACTTGACATACACTAA
	C4-6-L-r	AGGTTAGGACAGGGTACCAT

Region name	Primer name	Nucleotide sequences (5'-3')
C4-6	C4-6-R-f	ATGAAATCGATCATAGCGAT
	C4-6-R-r	CTGCAGCGTACGAAGCTTCAGCTGGCGGCCTCATCGTTTTCATCATAGGT
C4-7	C4-7-L-f	CTGCAGCGTACGAAGCTTCAGCTGGCGGCCCTTTCAATCTTGTCTCTTGC
	C4-7-L-r	GGAGAAACGCATCTAAGAAA
	C4-7-R-f	AAGGGGACTTTCAGGTGCAT
	C4-7-R-r	CTGCAGCGTACGAAGCTTCAGCTGGCGGCCCCTCATCGTGTGGCTTAACG
C4-8	C4-8-L-f	CTGCAGCGTACGAAGCTTCAGCTGGCGGCCTGAAGATTTTAAACTCACCT
	C4-8-L-r	CGGCCTTATTATGATCCCGA
C5-1	C5-1-R-f	CCCAATCATCTTAAGACAGC
	C5-1-R-r	CTGCAGCGTACGAAGCTTCAGCTGGCGGCCTGTAGACTCTTTAACACTCG
C5-2	C5-2-L-f	CTGCAGCGTACGAAGCTTCAGCTGGCGGCCGCGAACGAGTACATTATTGC
	C5-2-L-r	TGTATTCTACAGTTTGCTCC
	C5-2-R-f	AATAGAAGTGGAGCCTGTGG
	C5-2-R-r	CTGCAGCGTACGAAGCTTCAGCTGGCGGCCTATCATGCTGTACCCGCAAG
C5-3	C5-3-L-f	CTGCAGCGTACGAAGCTTCAGCTGGCGGCCTGGTAAGGCGTTGTGTTCCT
	C5-3-L-r	CATCTGCATCCACCAATGAA
C6-1	C6-1-R-f	ACGGTGCGCTCCAACGGATG
	C6-1-R-r	CTGCAGCGTACGAAGCTTCAGCTGGCGGCCTCCTTCCGATTCTGAAGGTG
C6-2	C6-2-L-f	CTGCAGCGTACGAAGCTTCAGCTGGCGGCCCCACAAGAAGTAATTACAGG
	C6-2-L-r	TGCAGAGAGTGCCGTAATCC
C7-1	C7-1-R-f	CGGTTGTATGATATAGATCC
	C7-1-R-r	CTGCAGCGTACGAAGCTTCAGCTGGCGGCCGGCCCAATCGAGCAAATAAG
C7-2	C7-2-L-f	CTGCAGCGTACGAAGCTTCAGCTGGCGGCCGCCGAACGTGTACCCGTAAA
	C7-2-L-r	CGCACCATTACAGGGTCAAA
	C7-2-R-f	AGGTTCTCTTCGCATAGTCG
	C7-2-R-r	CTGCAGCGTACGAAGCTTCAGCTGGCGGCCTCCAGAAGTTGGCATCTTTG
C7-3	C7-3-L-f	CTGCAGCGTACGAAGCTTCAGCTGGCGGCCGGTGGGTCTTGCTGAAAAGA
	C7-3-L-r	GCTTCAGAAAAGAGCCATAG
	C7-3-R-f	CCTACTTGGCGGTGAATTTC
	C7-3-R-r	CTGCAGCGTACGAAGCTTCAGCTGGCGGCCGATAAGCCCAATACACGACA
C7-4	C7-4-L-f	CTGCAGCGTACGAAGCTTCAGCTGGCGGCCAAGAAACTTCTCCAGAGGAG
	C7-4-L-r	CCGCCAAGAAGAGACGTAAA
	C7-4-R-f	TAATTACTTCGGTCGTGGCC
	C7-4-R-r	CTGCAGCGTACGAAGCTTCAGCTGGCGGCCGCTTTACTTAGTATGTCGGG
C7-5	C7-5-L-f	CTGCAGCGTACGAAGCTTCAGCTGGCGGCCCAATTTGTGGGATGATGACG
	C7-5-L-r	CAAGTCAGATAGCTTTGAGT
C8-1	C8-1-R-f	CGTCGGTTATGCTTGCCTAT
	C8-1-R-r	CTGCAGCGTACGAAGCTTCAGCTGGCGGCCGAGGAGGAGAAACGCATAAG
C8-2	C8-2-L-f	CTGCAGCGTACGAAGCTTCAGCTGGCGGCCCTCGTACATTGACTCAAACC
	C8-2-L-r	AGATTATGCACCTATCGGCG
	C8-2-R-f	AATCACCAGAAGCAGCAGCA
	C8-2-R-r	CTGCAGCGTACGAAGCTTCAGCTGGCGGCCGGCAGCAAGGTTGCCTTTAA
C8-3	C8-3-L-f	CTGCAGCGTACGAAGCTTCAGCTGGCGGCCGTAAAGCAGTTAGAACGTCG
	C8-3-L-r	GACACGGTATGTGGATACTC

Region name	Primer name	Nucleotide sequences (5'-3')
C9-1	C9-1-R-f	TTGTTGTTACCTCTCGTGTC
	C9-1-R-r	CTGCAGCGTACGAAGCTTCAGCTGGCGGCCGAGATCTAGAGTTAGTCAGG
C9-2	C9-2-L-f	CTGCAGCGTACGAAGCTTCAGCTGGCGGCCGGACAGTCCAGTACAGTTCA
	C9-2-L-r	GTGGTTCAAATATCCGTACG
C10-1	C10-1-R-f	GCATAATCGGCCCTCACAGA
	C10-1-R-r	CTGCAGCGTACGAAGCTTCAGCTGGCGGCCAGTAGTGAGGACAGGCTTAA
C10-2	C10-2-L-f	CTGCAGCGTACGAAGCTTCAGCTGGCGGCCTTGCTCGATCTTCTATCCTC
	C10-2-L-r	ACCCCAATAAAGGAAACGAA
	C10-2-R-f	GATTAGCCTACGAGCCATCA
	C10-2-R-r	CTGCAGCGTACGAAGCTTCAGCTGGCGGCCCACGGTTGTCATCAAAAAAG
C10-3	C10-3-L-f	CTGCAGCGTACGAAGCTTCAGCTGGCGGCCTAAACATAGATAAGCGAGCC
	C10-3-L-r	TTACGTCTGTTGAAGACGCC
	C10-3-R-f	GTAGAGGTCGATCACCTTCT
	C10-3-R-r	CTGCAGCGTACGAAGCTTCAGCTGGCGGCCTTGAGGTGACTGTGTTAAAC
C10-4	C10-4-L-f	CTGCAGCGTACGAAGCTTCAGCTGGCGGCCCGCACTAGCATTTGAAGACC
	C10-4-L-r	CATCAGTGCCAAGTTACACC
C11-1	C11-1-R-f	CAACCATTTCTCAAAGTGCT
	C11-1-R-r	CTGCAGCGTACGAAGCTTCAGCTGGCGGCCCATTGGCAATATGTACCAGA
C11-2	C11-2-L-f	CTGCAGCGTACGAAGCTTCAGCTGGCGGCCTGACTCTAAAACGGCATTTG
	C11-2-L-r	AAAGGGTTAAAGCAATCTCG
	C11-2-R-f	TGGCTTTGAAGAGAAGTCCT
	C11-2-R-r	CTGCAGCGTACGAAGCTTCAGCTGGCGGCCTATCGCTAAACAGTTCTTCC
C11-3	C11-3-L-f	CTGCAGCGTACGAAGCTTCAGCTGGCGGCCCCAACAGAAAGGTATTCCTT
	C11-3-L-r	CAGCATCAGAAGACCACAAA
C12-1	C12-1-R-f	ATGGATAGGTTTCGAGGGCA
	C12-1-R-r	CTGCAGCGTACGAAGCTTCAGCTGGCGGCCCGGTAACGTCAACAGTGGTA
C12-2	C12-2-L-f	CTGCAGCGTACGAAGCTTCAGCTGGCGGCCAATCCAAGAAGGAACCTGCG
	C12-2-L-r	CATAACGGTGCAAATACGTA
	C12-2-R-f	CCTGCTCTTATATCCGTTAT
	C12-2-R-r	CTGCAGCGTACGAAGCTTCAGCTGGCGGCCAGGCACCTATCGTCATTGTC
C12-3	C12-3-L-f	CTGCAGCGTACGAAGCTTCAGCTGGCGGCCCCTTCCCTATGATAAACTTC
	C12-3-L-r	TTCCCTTATAGCAGCAAGGG
	C-12-3-R-f	CCTAACGACGATGATAATAC
	C12-3-R-r	CTGCAGCGTACGAAGCTTCAGCTGGCGGCCTCTTGGAGACGTGTTCAGAA
C12-4	C12-4-L-f	CTGCAGCGTACGAAGCTTCAGCTGGCGGCCATGTCTCCTCTTCACCAAAG
	C12-4-L-r	TCCTCAACAACCTCTAATTC
	C12-4-R-f	GGAAAACGAAGAGCAGCAGC
	C12-4-R-r	CTGCAGCGTACGAAGCTTCAGCTGGCGGCCGCTATTATCCAGATGAAGGA
C12-5	C12-5-L-f	CTGCAGCGTACGAAGCTTCAGCTGGCGGCCCGATAAGCTAAGCCATTTTC
	C12-5-L-r	AGAAGACAACCCGTGGCTTG
C13-1	C13-1-R-f	GCCTCTATAGGCTTTTCGGA
	C13-1-R-r	CTGCAGCGTACGAAGCTTCAGCTGGCGGCCTGTAGCACCTACTTCTCATC
C13-2	C13-2-L-f	CTGCAGCGTACGAAGCTTCAGCTGGCGGCCCCAGCATTTTGTTATTGGCG
	C13-2-L-r	CCAGTATGTTCCCTTGACAA

Region name	Primer name	Nucleotide sequences (5'-3')
C13-2	C13-2-R-f	CCAGGAAACGTTCATTCAAT
	C13-2-R-r	CTGCAGCGTACGAAGCTTCAGCTGGCGGCCGAGCAAGTTGGCTGAATGTG
C13-3	C13-3-L-f	CTGCAGCGTACGAAGCTTCAGCTGGCGGCCGGACGACAGCCTGAATAATT
	C13-3-L-r	CTCTGATTTCAATGTCGTCT
	C13-3-R-f	TCAGAGGTCTGGAACATGTC
	C13-3-R-r	CTGCAGCGTACGAAGCTTCAGCTGGCGGCCACGGGAAGTACTAAGGTTGG
C13-4	C13-4-L-f	CTGCAGCGTACGAAGCTTCAGCTGGCGGCCGGTCTAAAGTCATCCACATG
	C13-4-L-r	AACAGTACTGGGATAGAAGG
	C13-4-R-f	GGGCAAAGGGACAAAATGAA
	C13-4-R-r	CTGCAGCGTACGAAGCTTCAGCTGGCGGCCTCATGGTTACCGTTACTGGC
C13-5	C13-5-L-f	CTGCAGCGTACGAAGCTTCAGCTGGCGGCCGAACTTACTTTCCTCTCTGC
	C13-5-L-r	AGAATTTCGAAGGAAAGGGG
C14-1	C14-1-R-f	TCCTCTTCCATCGATATCAG
	C14-1-R-r	CTGCAGCGTACGAAGCTTCAGCTGGCGGCCACAAGTATTGCACGAGACGT
C14-2	C14-2-L-f	CTGCAGCGTACGAAGCTTCAGCTGGCGGCCTGCACTGGAAATGCTTTTGG
	C14-2-L-r	AGTGCTCTACTGTCCGAGTC
	C14-2-R-f	GAGTCAACATTATAGGGCTG
	C14-2-R-r	CTGCAGCGTACGAAGCTTCAGCTGGCGGCCACGAAACTGTCGGGTTATCA
C14-3	C14-3-L-f	CTGCAGCGTACGAAGCTTCAGCTGGCGGCCTAATGCCATAATGTGGGGAC
	C14-3-L-r	TGCGGTTCTTAAAACTGTCG
	C14-3-R-f	AATACTATGGAGACCTTGGC
	C14-3-R-r	CTGCAGCGTACGAAGCTTCAGCTGGCGGCCATACGATAGAAGTACTGGGC
C14-4	C14-4-L-f	CTGCAGCGTACGAAGCTTCAGCTGGCGGCCTTAGAATGTGGGTCAGGTGG
	C14-4-L-r	GCATAGCCCTCTTTCGCCTC
C15-1	C15-1-R-f	CACCAGGTATTTGCCAATGG
	C15-1-R-r	CTGCAGCGTACGAAGCTTCAGCTGGCGGCCCCACTTTGCGTAACGCCAAA
C15-2	C15-2-L-f	CTGCAGCGTACGAAGCTTCAGCTGGCGGCCCAAATGGAATCGTTGCTGGG
	C15-2-L-r	CGGTTAAGTCGTCTAACGTC
	C15-2-R-f	GTGAGGGATGTCAGTTACTC
	C15-2-R-r	CTGCAGCGTACGAAGCTTCAGCTGGCGGCCCGTCTGAAGCCAATTGAGTG
C15-3	C15-3-L-f	CTGCAGCGTACGAAGCTTCAGCTGGCGGCCGCTTACTTAGTCCTTTGGTC
	C15-3-L-r	GCTTTTCCAATAAAGACGCA
	C15-3-R-f	GAAGGGATTGATCTCCGCTT
	C15-3-R-r	CTGCAGCGTACGAAGCTTCAGCTGGCGGCCCCAGGATCAAAATCTGGATG
C15-4	C15-4-L-f	CTGCAGCGTACGAAGCTTCAGCTGGCGGCCGCGTTATTGAGTGAACCGTC
	C15-4-L-r	CAGATGGTGCAGCCAATAGA
	C15-4-R-f	GATGTCCTCTGCAAGGATCT
	C15-4-R-r	CTGCAGCGTACGAAGCTTCAGCTGGCGGCCTCACTAGTGGTGCCACACTA
C15-5	C15-5-L-f	CTGCAGCGTACGAAGCTTCAGCTGGCGGCCCCAATTCACAATTTGTCGAT
	C15-5-L-r	TACAGGTCAATGAAAATGCG
C16-1	C16-1-R-f	CACCAAAGGCAAAGAAACTG
	C16-1-R-r	CTGCAGCGTACGAAGCTTCAGCTGGCGGCCATGCCCTTGAACTATGGACC
C16-2	C16-2-L-f	CTGCAGCGTACGAAGCTTCAGCTGGCGGCCAGAACAGGTGAGTCAGAAGA
	C16-2-L-r	GTGGATCTTGTGGTTGTCCG

Region name	Primer name	Nucleotide sequences (5'-3')
C16-2	C16-2-R-f	CATGGATGCTAATCCACTGT
	C16-2-R-r	CTGCAGCGTACGAAGCTTCAGCTGGCGGCCGCTAGACATGGTTGAAAATG
C16-3	C16-3-L-f	CTGCAGCGTACGAAGCTTCAGCTGGCGGCCGTCTTGACTGCTGCTTCTTG
	C16-3-L-r	GTAAAGCCATGTTTGATACC
	C16-3-R-f	TAGCCAGAACTTAAGTCAGG
	C16-3-R-r	CTGCAGCGTACGAAGCTTCAGCTGGCGGCCGTTGGTACCCCAAATTATTC
C16-4	C16-4-L-f	CTGCAGCGTACGAAGCTTCAGCTGGCGGCCACGGGTTTCTAGACAGCGAA
	C16-4-L-r	TGCGGCAAATTTTTCTGTGC
	C16-4-R-f	CATCGATTCTAGTCAAGAAG
	C16-4-R-r	CTGCAGCGTACGAAGCTTCAGCTGGCGGCCATCAGCCGTTTCACTCAGGT
C16-5	C16-5-L-f	CTGCAGCGTACGAAGCTTCAGCTGGCGGCCGAAATTAGACTTGGTACTGG
	C16-5-L-r	CATCCCGACTGATGGTGTAG

Supplementary Table 8. Primers used for construction of segmental chromosome duplications of sub-regions of unduplicated regions

Region name	Primer name	Nucleotide sequences (5'-3')
C4-2-S1	C4-2-L-f	CTGCAGCGTACGAAGCTTCAGCTGGCGGCCGTGCTCTTCTTGTTAACCCC
	C4-2-L-r	GGCCGCAATTGACGACACAC
	4-2-s1-R-f	AGGAACGCTGATCTTGATCT
	4-2-s1-R-r	CTGCAGCGTACGAAGCTTCAGCTGGCGGCCACTCTTGTATCCCACACAGG
C4-2-S2	4-2-s2-L-f	CTGCAGCGTACGAAGCTTCAGCTGGCGGCCTGAAGTCTTTGCATCCGTGG
	4-2-s2-L-r	ACCATCGGAGGGACTTTGA
	4-2-s2-R-f	TTCGTTCCTCAGCGGTGTGT
	4-2-s2-R-r	CTGCAGCGTACGAAGCTTCAGCTGGCGGCCAAGCTGCCAACTACCGTCAG
C4-2-S3	4-2-s3-L-f	CTGCAGCGTACGAAGCTTCAGCTGGCGGCCAGATTTTGTAGTGCTACGGA
	4-2-s3-L-r	AAAGGCTCTACACTCCCAGC
	4-2-s3-R-f	CTACACGGAAGTCAATCTCAC
	4-2-s3-R-r	CTGCAGCGTACGAAGCTTCAGCTGGCGGCCGCGGGTGCAGTCGTGTGCAG
C4-2-S4	4-2-s4-L-f	CTGCAGCGTACGAAGCTTCAGCTGGCGGCCTGGCTCTGAACTAGAAACCG
	4-2-s4-L-r	TTCTTGTCTCTGAGAATCGG
	C4-2-R-f	TCTTTTCATTATTGCTAGTA
	C4-2-R-r	CTGCAGCGTACGAAGCTTCAGCTGGCGGCCAAAGTAGTTCATGATGCGGG
C4-4-S1	C4-4-L-f	CTGCAGCGTACGAAGCTTCAGCTGGCGGCCAGGATTTTAATCTGTTGGAG
	C4-4-L-r	CCAACCAATATTACTGCTTT
	4-4-s1-R-f	CAGAAGACTGAAAGACTGCA
	4-4-s1-R-r	CTGCAGCGTACGAAGCTTCAGCTGGCGGCCCGAATCTTCTCGTCACGGAAG
C4-4-S2	4-4-s2-L-f	CTGCAGCGTACGAAGCTTCAGCTGGCGGCCTCCGTGACGAGAAGATTCGG
	4-4-s2-L-r	AACACTTCACTTTCAAGGCC
	4-4-s2-R-f	GTTGTAGTAATCTCGCGACC
	4-4-s2-R-r	CTGCAGCGTACGAAGCTTCAGCTGGCGGCCACCAATGGATCGAACGTGAG
C4-4-S3	4-4-s3-L-f	CTGCAGCGTACGAAGCTTCAGCTGGCGGCCGGGGAAGTTGCACTAAACGT
	4-4-s3-L-r	CAGATGGAACCAACCTAACC
	4-4-s3-R-f	GAACTGTCTGACTGCCGAAG
	4-4-s3-R-r	CTGCAGCGTACGAAGCTTCAGCTGGCGGCCGGCTTGTCACAATTTGCAGA
C4-4-S4	4-4-s4-L-f	CTGCAGCGTACGAAGCTTCAGCTGGCGGCCTAACTCGAAGGGTCACTGCC
	4-4-s4-L-r	CAATACCTACCATTAGCGAC
	C4-4-R-f	CCGACCGAGTATTACTCAGT
	C4-4-R-r	CTGCAGCGTACGAAGCTTCAGCTGGCGGCCGAGTCATCCATATTGCAAAC
C4-5-S1	C4-5-L-f	CTGCAGCGTACGAAGCTTCAGCTGGCGGCCCCAAAAGTTGCCTGTCCAAA
	C4-5-L-r	GAAGGCAAGGCTTACAGGCT
	4-5-s1-R-f	AGACTATTTTCATTGTTAAT
	4-5-s1-R-r	CTGCAGCGTACGAAGCTTCAGCTGGCGGCCATTCAATACTTTCACGTGTA
C4-5-S2	4-5-s2-L-f	CTGCAGCGTACGAAGCTTCAGCTGGCGGCCAACATTGTGCGCTCATCTAT
	4-5-s2-L-r	TGATCTAGCAATAATATCAA
	4-5-s2-R-f	AACCAGTGTCCTCGTTAATT
	4-5-s2-R-r	CTGCAGCGTACGAAGCTTCAGCTGGCGGCCTCTAATTTAAGAGATCAGAT
C4-5-S3	4-5-s3-L-f	CTGCAGCGTACGAAGCTTCAGCTGGCGGCCTTAGCCAAAAAGATCAATGT
	4-5-s3-L-r	CTAACATGTGACAATGAATG

Region name	Primer name	Nucleotide sequences (5'-3')
C4-5-S3	4-5-s3-R-f	CACAGGAATTTCAAGGTAGT
	4-5-s3-R-r	CTGCAGCGTACGAAGCTTCAGCTGGCGGCCTGATACTGATCTCCATATAC
C4-5-S4	4-5-s4-L-f	CTGCAGCGTACGAAGCTTCAGCTGGCGGCCCTACTCATCTTGATTAGTAT
	4-5-s4-L-r	ATCCTATCGTTTCAACTAGA
	C4-5-R-f	TTACGGTGGTTGCAAAGGGA
	C4-5-R-r	CTGCAGCGTACGAAGCTTCAGCTGGCGGCCAAAGAAGACTTCAATAAGTT
C4-7-S1	C4-7-L-f	CTGCAGCGTACGAAGCTTCAGCTGGCGGCCCTTTCAATCTTGTCTCTTGC
	C4-7-L-r	GGAGAAACGCATCTAAGAAA
	4-7-s1-R-f	CGGTGAATGGAATGCTGACA
	4-7-s1-R-r	CTGCAGCGTACGAAGCTTCAGCTGGCGGCCTGTTGAGCCACTTCCACTTG
C4-7-S2	4-7-s2-L-f	CTGCAGCGTACGAAGCTTCAGCTGGCGGCCAAATGGACCATCGTGGCGAT
	4-7-s2-L-r	GGCTCTATTCTGGCATTTCC
	4-7-s2-R-f	CTGTGTACGAGATTGTGACA
	4-7-s2-R-r	CTGCAGCGTACGAAGCTTCAGCTGGCGGCCTAATGCAAGAGTTGCCAGCG
C4-7-S3	4-7-s3-L-f	CTGCAGCGTACGAAGCTTCAGCTGGCGGCCGCTCTCACATGCTTTTTCTG
	4-7-s3-L-r	CCGAGTGGTTAGCTGCAACT
	4-7-s3-R-f	CTGCGACCGCTTTATTTGAC
	4-7-s3-R-r	CTGCAGCGTACGAAGCTTCAGCTGGCGGCCTAATAACGAGATGTACAGGC
C4-7-S4	4-7-s4-L-f	CTGCAGCGTACGAAGCTTCAGCTGGCGGCCGTATCCTTGCTTGGAAGCAG
	4-7-s4-L-r	ACCGACACCTCCTGCGATAG
	C4-7-R-f	AAGGGGACTTTCAGGTGCAT
	C4-7-R-r	CTGCAGCGTACGAAGCTTCAGCTGGCGGCCCCTCATCGTGTGGCTTAACG
C6-1-S1	SC6-1-R-f	ACGGCACCCTTTGTCAAGAG
	SC6-1-R-r	CTGCAGCGTACGAAGCTTCAGCTGGCGGCCAGGGGTGGATATCAACCTAC
C6-1-S2	SC6-2-L-f	CTGCAGCGTACGAAGCTTCAGCTGGCGGCCGTGTAGTTGCCTTCTTCACC
	SC6-2-L-r	CAAAGTCATGGGCTTCCCAG
	C6-1-R-f	ACGGTGCGCTCCAACGGATG
	C6-1-R-r	CTGCAGCGTACGAAGCTTCAGCTGGCGGCCTCCTTCCGATTCTGAAGGTG
C7-4-S1	C7-4-L-f	CTGCAGCGTACGAAGCTTCAGCTGGCGGCCAAGAAACTTCTCCAGAGGAG
	C7-4-L-r	CCGCCAAGAAGAGACGTAAA
	7-4-s1-R-f	GCGTAATTCACGGCGATAAC
	7-4-s1-R-r	CTGCAGCGTACGAAGCTTCAGCTGGCGGCCCTGATTCATGGGCCTCACGC
C7-4-S2	7-4-s2-L-f	CTGCAGCGTACGAAGCTTCAGCTGGCGGCCCAGTGATGTCGGAAACATCG
	7-4-s2-L-r	TTGTCTCACATCTGCATCTG
	7-4-s2-R-f	GCGTTTACCAATACTGGAATC
	7-4-s2-R-r	CTGCAGCGTACGAAGCTTCAGCTGGCGGCCCGCGCTCCTTTGTAGTGCCG
C7-4-S3	7-4-s3-L-f	CTGCAGCGTACGAAGCTTCAGCTGGCGGCCCTTACTGCGCAAGTGGCTCG
	7-4-s3-L-r	ATTGAACCTGACAGAAGCTG
	7-4-s3-R-f	TGTGCCGGCAGAATGTCGCG
	7-4-s3-R-r	CTGCAGCGTACGAAGCTTCAGCTGGCGGCCGTGTACCTATCAGGCCGCTG
C7-4-S4	7-4-s4-L-f	CTGCAGCGTACGAAGCTTCAGCTGGCGGCCTGGCTCATTGGACACAACCT
	7-4-s4-L-r	ACCTTAAACGGCTGAACAGG
	C7-4-R-f	TAATTACTTCGGTCGTGGCC
	C7-4-R-r	CTGCAGCGTACGAAGCTTCAGCTGGCGGCCGCTTTACTTAGTATGTCGGG
C8-2-S1	C8-2-L-f	CTGCAGCGTACGAAGCTTCAGCTGGCGGCCCTCGTACATTGACTCAAACC
	C8-2-L-r	AGATTATGCACCTATCGGCG

Region name	Primer name	Nucleotide sequences (5'-3')
C8-2-S1	8-2-s1-R-f	TATCACAAAAGCCCTCCATC
	8-2-s1-R-r	CTGCAGCGTACGAAGCTTCAGCTGGCGGCCACCGGCAATATGTCCTGCTTC
C8-2-S2	8-2-s2-L-f	CTGCAGCGTACGAAGCTTCAGCTGGCGGCCGATCCGAGTTTGAAACATCC
	8-2-s2-L-r	ACTGAATAGAAAGCGCTCT
	8-2-s2-R-f	TAAGTGATCACGTGGTCAGA
	8-2-s2-R-r	CTGCAGCGTACGAAGCTTCAGCTGGCGGCCTAGGAACTTCCTTTAGCTGG
C8-2-S3	8-2-s3-L-f	CTGCAGCGTACGAAGCTTCAGCTGGCGGCCCAAAGTAGGAACAGTGCCCG
	8-2-s3-L-r	GCCCTATTGAAGGTGAAGCC
	8-2-s3-R-f	CCCTTCCACCATCATTAC
	8-2-s3-R-r	CTGCAGCGTACGAAGCTTCAGCTGGCGGCCGGAAATTCGATGTTCAGGAG
C8-2-S4	8-2-s4-L-f	CTGCAGCGTACGAAGCTTCAGCTGGCGGCCAGGGTTCAGGAAAATTGCGG
	8-2-s4-L-r	CCTTTCACCAACGTACTCGA
	C8-2-R-f	AATCACCAGAAGCAGCAGCA
	C8-2-R-r	CTGCAGCGTACGAAGCTTCAGCTGGCGGCCGGCAGCAAGGTTGCCTTTAA
C11-2-S1	C11-2-L-f	CTGCAGCGTACGAAGCTTCAGCTGGCGGCCTGACTCTAAAACGGCATTTG
	C11-2-L-r	AAAGGGTTAAAGCAATCTCG
	SC11-1-R-f	CCCACATTGGTGTTCAAATG
	SC11-1-R-r	CTGCAGCGTACGAAGCTTCAGCTGGCGGCCTGCTCGTACCATAGACCTGG
C11-2-S2	SC11-2-L-f	CTGCAGCGTACGAAGCTTCAGCTGGCGGCCAAAGTAACGTCTCTGTTCGG
	SC11-2-L-r	GCAAAGTTACAGAACCGGTG
	SC11-2-R-f	GGGCATTGTTCAACATAGGG
	SC11-2-R-r	CTGCAGCGTACGAAGCTTCAGCTGGCGGCCTTTAACAGCTGAGCTGAACG
C11-2-S3	SC11-3-L-f	CTGCAGCGTACGAAGCTTCAGCTGGCGGCCATTTGAAACCGAGTTTGCGG
	SC11-3-L-r	GTTGATTACTGTCGATTCTG
	SC11-3-R-f	TGTCAAACTGCCAAGACGAC
	SC11-3-R-r	CTGCAGCGTACGAAGCTTCAGCTGGCGGCCACTTCCTTGTCCAGTATGGC
C11-2-S4	SC11-4-L-f	CTGCAGCGTACGAAGCTTCAGCTGGCGGCCCCTCCAGGATTTTTTTGGCA
	SC11-4-L-r	ACTTTAGGCAAGGTTGTTGC
	C11-2-R-f	TGGCTTTGAAGAGAAGTCCT
	C11-2-R-r	CTGCAGCGTACGAAGCTTCAGCTGGCGGCCTATCGCTAAACAGTTCTTCC
C14-2-S1	C14-2-L-f	CTGCAGCGTACGAAGCTTCAGCTGGCGGCCTGCACTGGAAATGCTTTTGG
	C14-2-L-r	AGTGCTCTACTGTCCGAGTC
	SC14-1-R-f	GGATGATCTGCCGATTTAGG
	SC14-1-R-r	CTGCAGCGTACGAAGCTTCAGCTGGCGGCCCCTCCTGGAGCTCTTCTAAT
C14-2-S2	SC14-2-L-f	CTGCAGCGTACGAAGCTTCAGCTGGCGGCCTGATTACGCGTCACAGCTAC
	SC14-2-L-r	ACCCTCAAGTCCTCCCTTGA
	SC14-2-R-f	TCTTCGAGGGGAAAATGTCG
	SC14-2-R-r	CTGCAGCGTACGAAGCTTCAGCTGGCGGCCTAGTTTGAGCCAGCACGATG
C14-2-S3	SC14-3-L-f	CTGCAGCGTACGAAGCTTCAGCTGGCGGCCCGGATAAATATTCTTCGAGGGG
	SC14-3-L-r	AGCACGATGGCAGGCCCTTA
	SC14-3-R-f	AGAAGATCTCGTTCATGACTGC
	SC14-3-R-r	CTGCAGCGTACGAAGCTTCAGCTGGCGGCCGTGTCGCCTTAATAGTCAGC
C14-2-S4	SC14-4-L-f	CTGCAGCGTACGAAGCTTCAGCTGGCGGCCTCGTCTTGCCGTATCTACAT
	SC14-4-L-r	GACCCAGATAGTGATGCTGA

Region name	Primer name	Nucleotide sequences (5'-3')
C14-2-S4	C14-2-R-f	GAGTCAACATTATAGGGCTG
	C14-2-R-r	CTGCAGCGTACGAAGCTTCAGCTGGCGGCCACGAAACTGTCGGGTTATCA
C4-2-S3+S4	4-2-s3-L-f	CTGCAGCGTACGAAGCTTCAGCTGGCGGCCAGATTTTGTAGTGCTACGGA
	4-2-s3-L-r	AAAGGCTCTACACTCCCAGC
	C4-2-R-f	TCTTTTCATTATTGCTAGTA
	C4-2-R-r	CTGCAGCGTACGAAGCTTCAGCTGGCGGCCAAAGTAGTTCATGATGCGGG
C4-4-S2+S3	4-4-s2-L-f	CTGCAGCGTACGAAGCTTCAGCTGGCGGCCTCCGTGACGAGAAGATTCGG
	4-4-s2-L-r	AACACTTCACTTTCAAGGCC
	4-4-s3-R-f	GAACTGTCTGACTGCCGAAG
	4-4-s3-R-r	CTGCAGCGTACGAAGCTTCAGCTGGCGGCCGGCTTGTCACAATTTGCAGA
C4-7-S3+S4	4-7-s3-L-f	CTGCAGCGTACGAAGCTTCAGCTGGCGGCCGCTCTCACATGCTTTTTCTG
	4-7-s3-L-r	CCGAGTGGTTAGCTGCAACT
	C4-7-R-f	AAGGGGACTTTCAGGTGCAT
	C4-7-R-r	CTGCAGCGTACGAAGCTTCAGCTGGCGGCCCCTCATCGTGTGGCTTAACG
C8-2-S3+S4	8-2-s3-L-f	CTGCAGCGTACGAAGCTTCAGCTGGCGGCCCAAAGTAGGAACAGTGCCCG
	8-2-s3-L-r	GCCCTATTGAAGGTGAAGCC
	C8-2-R-f	AATCACCAGAAGCAGCAGCA
	C8-2-R-r	CTGCAGCGTACGAAGCTTCAGCTGGCGGCCGGCAGCAAGGTTGCCTTTAA
C11-2-S1+S2	C11-2-L-f	CTGCAGCGTACGAAGCTTCAGCTGGCGGCCTGACTCTAAAACGGCATTTG
	C11-2-L-r	AAAGGGTTAAAGCAATCTCG
	SC11-2-R-f	GGGCATTGTTCAACATAGGG
	SC11-2-R-r	CTGCAGCGTACGAAGCTTCAGCTGGCGGCCTTTAACAGCTGAGCTGAACG
C14-2-S3+S4	SC14-3-L-f	CTGCAGCGTACGAAGCTTCAGCTGGCGGCCCGGATAAATATTCTTCGAGGGG
	SC14-3-L-r	AGCACGATGGCAGGCCCTTA
	C14-2-R-f	GAGTCAACATTATAGGGCTG
	C14-2-R-r	CTGCAGCGTACGAAGCTTCAGCTGGCGGCCACGAAACTGTCGGGTTATCA

Supplementary Table 9. Primers used for estimation of the maximum length of segmental chromosome duplication

Chromosomal region	Primer name	Nucleotide sequence (5'-3')
-	CA	CCCCAACCCCAACCCCAACCCCAACCCCAACCCCAAAGGCCACTAGTGGATCTGAT
-	loxP-cas	GGCCGCCAGCTGAAGCTTCG
Chr. I	C1-50k-L-f	CTGCAGCGTACGAAGCTTCAGCTGGCGGCCTAGCGTTGGTGAAAGGCACT
37,504 -87,735	C1-50k-L-r	GGTGCATAGTGTTTTAATGC
	C1-50k-R-f	AGAACGACCCCAGAATGTAC
	C1-50k-R-r	CTGCAGCGTACGAAGCTTCAGCTGGCGGCCCAGCAATGGGGACGATGATT
Chr. II	C2-150k-L-f	CTGCAGCGTACGAAGCTTCAGCTGGCGGCCTCTAAGCATCGACCTTAGAG
360,775-505,293	C2-150k-L-r	CAGACAAATCGCCATAGTCG
	C2-150k-R-f	CTGACCAAGAAAGAGCACGC
	C2-150k-R-r	CTGCAGCGTACGAAGCTTCAGCTGGCGGCCGGTGGAACTTGCATATCGTT
Chr. IV	C4-250k-L-f	CTGCAGCGTACGAAGCTTCAGCTGGCGGCCAACCCACAAAACGAGATGGA
148,203-401,638	C4-250k-L-r	TCCTTGTAGCGCTGATACGA
	C4-250k-R-f	TCTTTTCATTATTGCTAGTA
	C4-250k-R-r	CTGCAGCGTACGAAGCTTCAGCTGGCGGCCAAAGTAGTTCATGATGCGGG
Chr. IV	C4-300k-L-f	CTGCAGCGTACGAAGCTTCAGCTGGCGGCCATTCGATTTCCACTGCTTAT
97,475-401,638	C4-300k-L-r	CCTCGCATAAATTGGGAAAT
	C4-300k-R-f	TCTTTTCATTATTGCTAGTA
	C4-300k-R-r	CTGCAGCGTACGAAGCTTCAGCTGGCGGCCAAAGTAGTTCATGATGCGGG
Chr. IV	C4-350k-L-f	CTGCAGCGTACGAAGCTTCAGCTGGCGGCCCAAACAACATTTGTCCAAAA
50,000 - 401,638	C4-350k-L-r	TTCTGCAAACCAAAGAAAGA
	C4-350k-R-f	TCTTTTCATTATTGCTAGTA
	C4-350k-R-r	CTGCAGCGTACGAAGCTTCAGCTGGCGGCCAAAGTAGTTCATGATGCGGG
Chr. IV	C4-400k-L-f	CTGCAGCGTACGAAGCTTCAGCTGGCGGCCGTGCTCTTCTTGTTAACCCC
198,996-600,688	C4-400k-L-r	GGCCGCAATTGACGACACAC
	C4-400k-R-f	TCGAGGACAAAAAGGCATAT
	C4-400k-R-r	CTGCAGCGTACGAAGCTTCAGCTGGCGGCCGAGAATAAAATAGGTCAGGT
Chr. VIII	C8-50k-L-f	CTGCAGCGTACGAAGCTTCAGCTGGCGGCCCTCCTAGATGGTGGGATCCA
294,748- 346,028	C8-50k-L-r	GGCCAAACGGTCAAGATCAA
	C8-50k-R-f	GACTGGTTTTAATGGTATTG
	C8-50k-R-r	CTGCAGCGTACGAAGCTTCAGCTGGCGGCCGACCTCTTATAAAGATTCAA
Chr. VIII	C8-100k-L-f	CTGCAGCGTACGAAGCTTCAGCTGGCGGCCTTTGCGCAACTGTTGCCGTG
247,693-346,028	C8-100k-L-r	TTAACTTTGGGGACCATTGA
	C8-100k-R-f	GACTGGTTTTAATGGTATTG
	C8-100k-R-r	CTGCAGCGTACGAAGCTTCAGCTGGCGGCCGACCTCTTATAAAGATTCAA
Chr. VIII	C8-150k-L-f	CTGCAGCGTACGAAGCTTCAGCTGGCGGCCAGCGTGTCGCGTTCCTCGAA
192,203-346,028	C8-150k-L-r	TGGTATCTACCTGAAGTCTT
	C8-150k-R-f	GACTGGTTTTAATGGTATTG
	C8-150k-R-r	CTGCAGCGTACGAAGCTTCAGCTGGCGGCCGACCTCTTATAAAGATTCAA

Chromosomal region	Primer name	Nucleotide sequence (5'-3')
Chr. VIII	C8-200k-L-f	CTGCAGCGTACGAAGCTTCAGCTGGCGGCCTTCGTAGAAATGACTCCAAG
145,656-346,028	C8-200k-L-r	GAACGACCGAACATACAGTA
	C8-200k-R-f	GACTGGTTTTAATGGTATTG
	C8-200k-R-r	CTGCAGCGTACGAAGCTTCAGCTGGCGGCCGACCTCTTATAAAGATTCAA
Chr. X	C10-100k-L-f	CTGCAGCGTACGAAGCTTCAGCTGGCGGCCACAGACAAGGTCATATCGCG
225,115-326,063	C10-100k-L-r	CTCTCATGGAGGGTGTAATT
	C10-100k-R-f	TTCCATTGACCACCGTCTAC
	C10-100k-R-r	CTGCAGCGTACGAAGCTTCAGCTGGCGGCCGCGAACTCTGTTTCATCAGG

Supplementary Table 10. Primers used to amplify probes for detection of segmental chromosome duplications of chromosomes I to XVI

Region name	Primer name	Nucleotide sequences (5'-3')
C1-1	C1-1-p-f	TTTTCCGGACCCAAACAACC
	C1-1-p-r	TCTGTGGAGACCAATCGAGG
C1-2	C1-2-p-f	GCCAGTGTAACTCCTCACTG
	C1-2-p-f	AGAACCAGGCCTTCCACTTT
C2-1	C2-1-R-f	TTACATGCGACACCAAGCAG
	C2-1-R-r	CTGCAGCGTACGAAGCTTCAGCTGGCGGCCGTCCTCCGAGGCAGGCCCTC
C2-2	C2-2-L-f	CTGCAGCGTACGAAGCTTCAGCTGGCGGCCTGAATGCAATTCGATACTCG
	C2-2-L-r	CAATCCAGTGATACCCGTGG
C2-3	C2-3-R-f	TCTCTGAGGGTTATCAAATG
	C2-3-R-r	CTGCAGCGTACGAAGCTTCAGCTGGCGGCCCGTGTGATGTGGACTGTTGC
C2-4	C2-4-L-f	CTGCAGCGTACGAAGCTTCAGCTGGCGGCCCTAACCCTTTGATGTCCGAC
	C2-4-L-r	CTTTTCTTCCCTCCAAGATC
C3-1	C3-1-p-f	GCAAGACTCTGGTCTCTTCT
	C3-1-p-r	ACACCTGAGTGGGTCATCAC
C3-2	C3-2-p-f	CTCTTAGCGGACCGTTTTGG
	C3-2-p-r	ATCTCTCCGCAGGGGTAAGC
C4-1	C4-1-R-f	AGGGCATCCATCCAACCATC
	C4-1-R-r	CTGCAGCGTACGAAGCTTCAGCTGGCGGCCGGCTTTGGAGGAGATATTTG
C4-2	C4-2-L-f	CTGCAGCGTACGAAGCTTCAGCTGGCGGCCGTGCTCTTCTTGTTAACCCC
	C4-2-L-r	GGCCGCAATTGACGACACAC
C4-3	C4-3-R-f	TCGAGGACAAAAAGGCATAT
	C4-3-R-r	CTGCAGCGTACGAAGCTTCAGCTGGCGGCCGAGAATAAAATAGGTCAGGT
C4-4	C4-4-L-f	CTGCAGCGTACGAAGCTTCAGCTGGCGGCCAGGATTTTAATCTGTTGGAG
	C4-4-L-r	CCAACCAATATTACTGCTTT
C4-5	C4-5-L-f	CTGCAGCGTACGAAGCTTCAGCTGGCGGCCCCAAAAGTTGCCTGTCCAAA
	C4-5-L-r	GAAGGCAAGGCTTACAGGCT
C4-6	C4-6-L-f	CTGCAGCGTACGAAGCTTCAGCTGGCGGCCGTTGACTTGACATACACTAA
	C4-6-L-r	AGGTTAGGACAGGGTACCAT
C4-7	C4-7-L-f	CTGCAGCGTACGAAGCTTCAGCTGGCGGCCCTTTCAATCTTGTCTCTTGC
	C4-7-L-r	GGAGAAACGCATCTAAGAAA
C4-8	C4-8-L-f	CTGCAGCGTACGAAGCTTCAGCTGGCGGCCTGAAGATTTTAAACTCACCT
	C4-8-L-r	CGGCCTTATTATGATCCCGA
C5-1	C5-1P-f	ATAAAGCAGCTGAACTTTCC
	C5-1P-r	CATTTTCGTTGTGGGCACAC
C5-2	C5-2P-f	CATATATCAGAGTCACAGCT
	C5-2P-r	CCACCACCCAAAAGAGTGTC
C5-3	C5-3P-f	TGAAGTGTGGAATCTGTCTC
	C5-3P-r	TGGAGGAGATGATGAAGCAA
C6-1	C6-1-p-f	CCACTCGTTGCCGGAGGCAC
	C6-1-p-r	GAACCCTGGCGACTTTTGGA

Region name	Primer name	Nucleotide sequences (5'-3')
C6-2	C6-2-p-f	ACGAGCCCTTGACTGAGCAG
	C6-2-p-r	AAGACCGCCTCCAGCAGTTG
C7-1	C7-1-R-f	CGGTTGTATGATATAGATCC
	C7-1-R-r	CTGCAGCGTACGAAGCTTCAGCTGGCGGCCGGCCCAATCGAGCAAATAAG
C7-2	C7-2-L-f	CTGCAGCGTACGAAGCTTCAGCTGGCGGCCGCCGAACGTGTACCCGTAAA
	C7-2-L-r	CGCACCATTACAGGGTCAAA
C7-3	C7-3-R-f	CCTACTTGGCGGTGAATTTC
	C7-3-R-r	CTGCAGCGTACGAAGCTTCAGCTGGCGGCCGATAAGCCCAATACACGACA
C7-4	C7-4-L-f	CTGCAGCGTACGAAGCTTCAGCTGGCGGCCAAGAAACTTCTCCAGAGGAG
	C7-4-L-r	CCGCCAAGAAGAGACGTAAA
C7-5	C7-5-L-f	CTGCAGCGTACGAAGCTTCAGCTGGCGGCCCAATTTGTGGGATGATGACG
	C7-5-L-r	CAAGTCAGATAGCTTTGAGT
C8-1	C8-1 probe-f	TGGATGGTGCATTCTTAGAG
	C8-1 probe-r	TGGGTAAGGAAATGAGAGCA
C8-2	C8-2 probe-f	CACAATCACCGAGCGTCTTT
	C8-2 probe-r	ATATGTGACCAATGCGGGAT
C8-3	C8-3 probe-f	CCTACAGAGCGTGAAATGCA
	C8-3 probe-r	CGACTCATCGAAGGTTCATA
C9-1	C9-1 probe-f	GGTGTTGTAAACCCCTCAAG
	C9-1 probe-r	ATAACCTTGCCGTCAATGTC
C9-2	C9-2 probe-f	TCAGCAGATTCGATGGATGC
	C9-2 probe-r	GACGAATTCATCAAGACGCA
C10-1	C10-1 probe-r	GTAAAATCGATGAGTGGGGA
	C10-1 probe-f	CAGCACAACGCTCTAACATA
C10-2	C10-2 probe-r	TGACTGACGAATCGTTAGGC
	C10-2 probe-f	CTTGCGATTTCTTCGTATGC
C10-3	C10-3 probe-f	GGGAAACTGCATGTAGTTGT
	C10-3 probe-r	ATACCCGGAAGACAGAATCG
C10-4	C10-4 probe-f	GTCGTTCGGCGAAACCTTAT
	C10-4 probe-r	CAACAGTCGTAGCTAACGAG
C11-1	C11-1P-f	AGATACAGCCTGTTGACCAA
	C11-1P-r	ACCAAACGCGTTTGGCAATA
C11-2	C11-2P-f	GACGAGAATAACCAAGGGCA
	C11-2P-r	GGAGTTGCTTTGTTTTGTTC
C11-3	C11-3P-f	GGCTACAAGAAACTTCGTGC
	C11-3P-r	TCGACATGTGTCCTCCATGT
C12-1	C12-1-R-f	ATGGATAGGTTTCGAGGGCA
	C12-1-R-r	CTGCAGCGTACGAAGCTTCAGCTGGCGGCCCGGTAACGTCAACAGTGGTA
C12-2	C12-2-L-f	CTGCAGCGTACGAAGCTTCAGCTGGCGGCCAATCCAAGAAGGAACCTGCG
	C12-2-L-r	CATAACGGTGCAAATACGTA
C12-3	C-12-3-R-f	CCTAACGACGATGATAATAC
	C12-3-R-r	CTGCAGCGTACGAAGCTTCAGCTGGCGGCCTCTTGGAGACGTGTTCAGAA
C12-4	C12-4-R-f	GGAAAACGAAGAGCAGCAGC
	C12-4-R-r	CTGCAGCGTACGAAGCTTCAGCTGGCGGCCGCTATTATCCAGATGAAGGA

Region name	Primer name	Nucleotide sequences (5'-3')
C12-5	C12-5-L-f	CTGCAGCGTACGAAGCTTCAGCTGGCGGCCCGATAAGCTAAGCCATTTTC
	C12-5-L-r	AGAAGACAACCCGTGGCTTG
C13-1	C13-1-R-f	GCCTCTATAGGCTTTTCGGA
	C13-1-R-r	CTGCAGCGTACGAAGCTTCAGCTGGCGGCCTGTAGCACCTACTTCTCATC
C13-2	C13-2-L-f	CTGCAGCGTACGAAGCTTCAGCTGGCGGCCCCAGCATTTTGTTATTGGCG
	C13-2-L-r	CCAGTATGTTCCCTTGACAA
C13-3	C13-3-L-f	CTGCAGCGTACGAAGCTTCAGCTGGCGGCCGGACGACAGCCTGAATAATT
	C13-3-L-r	CTCTGATTTCAATGTCGTCT
C13-4	C13-4-L-f	CTGCAGCGTACGAAGCTTCAGCTGGCGGCCGGTCTAAAGTCATCCACATG
	C13-4-L-r	AACAGTACTGGGATAGAAGG
C13-5	C13-5-L-f	CTGCAGCGTACGAAGCTTCAGCTGGCGGCCGAACTTACTTTCCTCTCTGC
	C13-5-L-r	AGAATTTCGAAGGAAAGGGG
C14-1	C14-p1-f	AACAATGGCACTCATGCAGC
	C14-p1-r	CAGCGCTTCCACGGCATACC
C14-2	C14-p2-f	GACACGTAATCGGAGTTTGC
	C14-p2-r	GCAGTAGGTAAAACGTCACT
C14-3	C14-p3-f	TGGTAACTCTGTTGAAGACG
	C14-p3-r	GCCGAAGAACAAGAGAAAGC
C14-4	C14-p4-f	CAAAGTAGCAAGGTAATCGG
	C14-p4-r	ACGATATCATCGGTTCGCTG
C15-1	C15-1-R-f	CACCAGGTATTTGCCAATGG
	C15-1-R-r	CTGCAGCGTACGAAGCTTCAGCTGGCGGCCCCACTTTGCGTAACGCCAAA
C15-2	C15-2-R-f	GTGAGGGATGTCAGTTACTC
	C15-2-R-r	CTGCAGCGTACGAAGCTTCAGCTGGCGGCCCGTCTGAAGCCAATTGAGTG
C15-3	C15-3-L-f	CTGCAGCGTACGAAGCTTCAGCTGGCGGCCGCTTACTTAGTCCTTTGGTC
	C15-3-L-r	GCTTTTCCAATAAAGACGCA
C15-4	C15-4-L-f	CTGCAGCGTACGAAGCTTCAGCTGGCGGCCGCGTTATTGAGTGAACCGTC
	C15-4-L-r	CAGATGGTGCAGCCAATAGA
C15-5	C15-5-L-f	CTGCAGCGTACGAAGCTTCAGCTGGCGGCCCCAATTCACAATTTGTCGAT
	C15-5-L-r	TACAGGTCAATGAAAATGCG
C16-1	C16-1-R-f	CACCAAAGGCAAAGAAACTG
	C16-1-R-r	CTGCAGCGTACGAAGCTTCAGCTGGCGGCCATGCCCTTGAACTATGGACC
C16-2	C16-2-L-f	CTGCAGCGTACGAAGCTTCAGCTGGCGGCCAGAACAGGTGAGTCAGAAGA
	C16-2-L-r	GTGGATCTTGTGGTTGTCCG
C16-3	C16-3-L-f	CTGCAGCGTACGAAGCTTCAGCTGGCGGCCGTCTTGACTGCTGCTTCTTG
	C16-3-L-r	GTAAAGCCATGTTTGATACC
C16-4	C16-4-L-f	CTGCAGCGTACGAAGCTTCAGCTGGCGGCCACGGGTTTCTAGACAGCGAA
	C16-4-L-r	TGCGGCAAATTTTTCTGTGC
C16-5	C16-5-L-f	CTGCAGCGTACGAAGCTTCAGCTGGCGGCCGAAATTAGACTTGGTACTGG
	C16-5-L-r	CATCCCGACTGATGGTGTAG

Supplementary Table 11. Primers used to amplify probes for detection of segmental chromosome duplication of sub-regions

Region name	Primer name	Nucleotide sequences (5'-3')
C4-2-S1	4-2-s1-R-f	AGGAACGCTGATCTTGATCT
	4-2-s1-R-r	CTGCAGCGTACGAAGCTTCAGCTGGCGGCCACTCTTGTATCCCACACAGG
C4-2-S2	4-2-s2-R-f	TTCGTTCCTCAGCGGTGTGT
	4-2-s2-R-r	CTGCAGCGTACGAAGCTTCAGCTGGCGGCCAAGCTGCCAACTACCGTCAG
C4-2-S3	4-2-s3-L-f	CTGCAGCGTACGAAGCTTCAGCTGGCGGCCAGATTTTGTAGTGCTACGGA
	4-2-s3-L-r	AAAGGCTCTACACTCCCAGC
C4-2-S4	4-2-s4-L-f	CTGCAGCGTACGAAGCTTCAGCTGGCGGCCTGGCTCTGAACTAGAAACCG
	4-2-s4-L-r	TTCTTGTCTCTGAGAATCGG
C4-4-S1	C4-4-L-f	CTGCAGCGTACGAAGCTTCAGCTGGCGGCCAGGATTTTAATCTGTTGGAG
	C4-4-L-r	CCAACCAATATTACTGCTTT
C4-4-S2	4-4-s2-R-f	GTTGTAGTAATCTCGCGACC
	4-4-s2-R-r	CTGCAGCGTACGAAGCTTCAGCTGGCGGCCACCAATGGATCGAACGTGAG
C4-4-S3	4-4-s3-R-f	GAACTGTCTGACTGCCGAAG
	4-4-s3-R-r	CTGCAGCGTACGAAGCTTCAGCTGGCGGCCGGCTTGTCACAATTTGCAGA
C4-4-S4	C4-4-R-f	CCGACCGAGTATTACTCAGT
	C4-4-R-r	CTGCAGCGTACGAAGCTTCAGCTGGCGGCCGAGTCATCCATATTGCAAAC
C4-5-S1	4-5-s1-R-f	AGACTATTTTCATTGTTAAT
	4-5-s1-R-r	CTGCAGCGTACGAAGCTTCAGCTGGCGGCCATTCAATACTTTCACGTGTA
C4-5-S2	4-5-s2-L-f	CTGCAGCGTACGAAGCTTCAGCTGGCGGCCAACATTGTGCGCTCATCTAT
	4-5-s2-L-r	TGATCTAGCAATAATATCAA
C4-5-S3	4-5-s3-L-f	CTGCAGCGTACGAAGCTTCAGCTGGCGGCCTTAGCCAAAAAGATCAATGT
	4-5-s3-L-r	CTAACATGTGACAATGAATG
C4-5-S4	4-5-s4-L-f	CTGCAGCGTACGAAGCTTCAGCTGGCGGCCCTACTCATCTTGATTAGTAT
	4-5-s4-L-r	ATCCTATCGTTTCAACTAGA
C4-7-S1	C4-7-S1	CGGTGAATGGAATGCTGACA
	4-7-s1-R-r	CTGCAGCGTACGAAGCTTCAGCTGGCGGCCTGTTGAGCCACTTCCACTTG
C4-7-S2	4-7-s2-L-f	CTGCAGCGTACGAAGCTTCAGCTGGCGGCCAAATGGACCATCGTGGCGAT
	4-7-s2-L-r	GGCTCTATTCTGGCATTTCC
C4-7-S3	4-7-s3-R-f	CTGCGACCGCTTTATTTGAC
	4-7-s3-R-r	CTGCAGCGTACGAAGCTTCAGCTGGCGGCCTAATAACGAGATGTACAGGC
C4-7-S4	4-7-s4-L-f	CTGCAGCGTACGAAGCTTCAGCTGGCGGCCGTATCCTTGCTTGGAAGCAG
	4-7-s4-L-r	ACCGACACCTCCTGCGATAG
C6-1-S1	SC6-1-p-f	GGAAGATGGATGCCCTTGTT
	SC6-1-p-r	ACTTCCAGACAACACAGGGG
C6-1-S2	SC6-2-p-f	ACTTCCAGACAACACAGGGG
	SC6-2-p-r	GAGCAGCTCTTCTGTTTCTC
C7-4-S1	C7-4-L-f	CTGCAGCGTACGAAGCTTCAGCTGGCGGCCAAGAAACTTCTCCAGAGGAG
	C7-4-L-r	CCGCCAAGAAGAGACGTAAA
C7-4-S2	7-4-s2-R-f	GCGTTTACCAATACTGGAATC
	7-4-s2-R-r	CTGCAGCGTACGAAGCTTCAGCTGGCGGCCCGCGCTCCTTTGTAGTGCCG

Region name	Primer name	Nucleotide sequences (5'-3')
C7-4-S3	7-4-s3-L-f	CTGCAGCGTACGAAGCTTCAGCTGGCGGCCCTTACTGCGCAAGTGGCTCG
	7-4-s3-L-r	ATTGAACCTGACAGAAGCTG
C7-4-S4	C7-4-R-f	TAATTACTTCGGTCGTGGCC
	C7-4-R-r	CTGCAGCGTACGAAGCTTCAGCTGGCGGCCGCTTTACTTAGTATGTCGGG
C8-2-S1	8-2-s1-R-f	TATCACAAAAGCCCTCCATC
	8-2-s1-R-r	CTGCAGCGTACGAAGCTTCAGCTGGCGGCCACCGGCAATATGTCCTGCTTC
C8-2-S2	8-2-s2-R-f	TAAGTGATCACGTGGTCAGA
	8-2-s2-R-r	CTGCAGCGTACGAAGCTTCAGCTGGCGGCCTAGGAACTTCCTTTAGCTGG
C8-2-S3	8-2-s3-R-f	CCCTTCCACCATCATTAC
	8-2-s3-R-r	CTGCAGCGTACGAAGCTTCAGCTGGCGGCCGGAAATTCGATGTTCAGGAG
C8-2-S4	8-2-s4-L-f	CTGCAGCGTACGAAGCTTCAGCTGGCGGCCAGGGTTCAGGAAAATTGCGG
	8-2-s4-L-r	CCTTTCACCAACGTACTCGA
C11-2-S1	SC11-1-R-f	CCCACATTGGTGTTCAAATG
	SC11-1-R-r	CTGCAGCGTACGAAGCTTCAGCTGGCGGCCTGCTCGTACCATAGACCTGG
C11-2-S2	SC11-2-L-f	CTGCAGCGTACGAAGCTTCAGCTGGCGGCCAAAGTAACGTCTCTGTTCGG
	SC11-2-L-r	GCAAAGTTACAGAACCGGTG
C11-2-S3	SC11-3-L-f	CTGCAGCGTACGAAGCTTCAGCTGGCGGCCATTTGAAACCGAGTTTGCGG
	SC11-3-L-r	GTTGATTACTGTCGATTCTG
C11-2-S4	SC11-4-L-f	CTGCAGCGTACGAAGCTTCAGCTGGCGGCCCCTCCAGGATTTTTTTGGCA
	SC11-4-L-r	ACTTTAGGCAAGGTTGTTGC
C14-2-S1	SC14-1-p-f	GAATACTGGCCTCTGCCTCA
	SC14-1-p-r	AGCGCTGGATACAGAAACGT
C14-2-S2	SC14-2-p-f	CGGAAGTGGTATCCGAACCA
	SC14-2-p-r	CAGATCGGTAAAGGAGACGG
C14-2-S3	SC14-3-p-f	GACTCCCAATGCGATAAACC
	SC14-3-p-r	GGAGATAACCCAGCGGTCTT
C14-2-S4	SC14-4-p-f	GTTGTGACGAAGTGTGTAGG
	SC14-4-p-r	AGGAAGTCCCTGCGAGATCA

Supplementary Table 12. Primers used to amplify probes for estimation of the maximum length of segmental chromosome duplication

Chromosomal region	Primer name	Nucleotide sequence (5'-3')
Chr. I	C1-50k-R-f	AGAACGACCCCAGAATGTAC
87,336-87,735	C1-50k-R-r	CTGCAGCGTACGAAGCTTCAGCTGGCGGCCCAGCAATGGGGACGATGATT
Chr. II	C2-150k-R-f	CTGACCAAGAAAGAGCACGC
504,894-505,293	C2-150k-R-r	CTGCAGCGTACGAAGCTTCAGCTGGCGGCCGGTGGAACTTGCATATCGTT
Chr. VIII	Chr.8-probe3-f	CAAGTCCGTGCTGTCAAGGA
325,648-326,147	Chr.8-probe3-r	CAATAACGGCCAATGGCTTG
Chr. IV	C4-250k-R-f	TCTTTTCATTATTGCTAGTA
401,239-401,638	C4-250k-R-r	CTGCAGCGTACGAAGCTTCAGCTGGCGGCCAAAGTAGTTCATGATGCGGG
Chr. X	C10-check-f	CTGATGAATGGACAATGCAT
247,685-248,184	C10-check-r	GCTCGATGATGAGCCTCTTA

References

57. Roth, D., Birkenfeld, J. & Betz, H. Dominant-negative alleles of 14-3-3 proteins cause defects in actin organization and vesicle targeting in the yeast *Saccharomyces cerevisiae*. *FEBS Lett.* **460,** 411–416 (1999).

58. Sugiyama, M. *et al.* PCR-mediated one-step deletion of targeted chromosomal regions in haploid *Saccharomyces cerevisiae*. *Appl. Microbiol. Biotechnol.* **80,** 545–53 (2008).

59. Rose, M. D., Novick, P., Thomas, J. H., Botstein, D. & Fink, G. R. A *Saccharomyces cerevisiae* genomic plasmid bank based on a centromere-containing shuttle vector. *Gene* **60,** 237–43 (1987).
